# Supplementary material for: Rattle drum-inspired triboelectric nanogenerator with enhanced output using charge dispatch and magnetic repulsion pendulum
Source: Nat Commun. 2025 Oct 29;16:9539. doi: 10.1038/s41467-025-64575-9 (PMC12572158; doi:10.1038/s41467-025-64575-9)
Supplement: Supplementary file 1 — Supplementary Information [file 41467_2025_64575_MOESM1_ESM.pdf]

Supplementary Information

Rattle Drum Inspired Triboelectric Nanogenerator with Enhanced Output Using Charge Dispatch and Magnetic Repulsion Pendulum

Wei Tang[1,2], Hongfang Li[1,2], Jiawei Li[1,2], Weiyu Zhou[1,2], Jiaqi Duan[1,2], Yongsheng Wen[1,2], Lingyu Wan[1,2]✉ & Guanlin Liu[1,2]✉

[1]Center on Nanoenergy Research, Institute of Science and Technology for Carbon Peak & Neutrality, School of Physical Science & Technology, Guangxi University, Nanning 530004, China. [2]State Key Laboratory of Featured Metal Materials and Life-cycle Safety for Composite Structures, Guangxi University, Nanning 530004, China. ✉ Email: lyw2017@gxu.edu.cn, guanlinliu@gxu.edu.cn

Content

Supplementary Table

Table 1. Comparison of RD-TENG output with traditional FCS structures.
Table 2. Comparison between previously reported three-electrode configurations and the approach presented in this work
Table 3. Comprehensive evaluation metric. Abbreviations: TSD, triboelectric surface density; SSA, specific surface area; VPD, volumetric power density; VCD, volumetric charge density; SCD, surface charge density.
Table 4. Factor-level table.
Table 5. Orthogonal table.
Table 6. Table header design of experiment 1.
Table 7. Experimental setup and data acquisition (see Supplementary Figure 13).
Table 8. Data analysis.
Table 9. Factor-level table.
Table 10. Table header design of combination A for the linear drive of experiment 2.
Table 11. Table header design of combination B for swinging drive of experiment 2.
Table 12. Experimental setup and data acquisition of combination A for the linear drive.
Table 13. Data analysis of combination A for the linear drive.
Table 14. Experimental setup and data acquisition of combination B for the swinging drive.
Table 15. Data analysis of combination B for the swinging drive.
Table 16. Material parameters of Mn65 spring steel (based on Chinese National Standard GB/T 1222-2016, ASTM A689, and the Mechanical Engineering Materials Handbook)
Table 17. Factors affecting the magnetic repulsion constant (A).

Supplementary Note

Note 1. The difficulties arise after the densification of friction layers in traditional FCS-TENG.
1. Charge cancellation issue.
2. Electrostatic shielding issue.
3. Low contact-separation efficiency issue.
Note 2. Inspiration from the rattle drum to the prototype device.
Note 3. From the rattle-drum-model TENG to the three-electrode RD-TENG.
Note 4. Essential differences between the three-electrode RD-TENG and previous works.
Note 5. Quantitative theoretical model.
1. Strategy.
2. For traditional configurations (applicable to Structures 1, 2, and 3):
3. For the RD-TENG configuration:
Note 6. Construction of a comprehensive performance evaluation system for TENG systems
Note 7. Orthogonal experimental design.
Experiment 1: Orthogonal experimental design of RD-TENG under direct linear drive.
Experiment 2: Orthogonal experimental design of RD-TENG under linear drive and swinging drive based on magnetic repulsion pendulum.
Note 8. Discussion on materials, engineering choices, scalability, limitations, and future strategies.

1. Selection of core materials and engineering design strategies:
2. Strategy scalability.
3. Potential limitations and future mitigation strategies.

Supplementary Figures

Figure 1. Charge cancellation phenomenon caused by shared electrodes in stacked FCS models (the electrode is grounded).
Figure 2. Three traditional FCS models were designed and constructed in this work.
Figure 3. Schematic decomposition of the basic unit in the traditional FCS model.
Figure 4. (a) Rattle-drum. (b) rattle-drum-type TENG. (c) RD-TENG
Figure 5. State correspondence diagram from the rattle-drum-type prototype TENG model (a) to the RD-TENG model (b), the electrode is grounded.
Figure 6. Three-electrode configuration of the RD-TENG device.
Figure 7. Decomposition diagram of the fundamental unit of the RD-TENG model.
Figure 8. Equivalent capacitive circuit network models of traditional.
Figure 9. Detailed parameters.
Figure 10. RD-TENG Model Visualization: Relationship between the short-circuit transferred charge and open-circuit voltage output with respect to the displacement x of the vibrator between the electrodes.
Figure 11. Relationship among various evaluation metrics systems. Positive feedback refers to a process where an increase in one quantity promotes the growth of another. In contrast, negative feedback denotes a process where an increase in one quantity suppresses the other.
Figure 12. Comparison of the structural and electrical performance metrics with other works.
Figure 13. The original data acquisition diagram of the orthogonal experimental design of RD-TENG under direct linear drive.
Figure 14. The output variation trend of RD-TENG under direct linear drive concerning each input parameter.
Figure 15. The output variation trend of RD-TENG is based on the frequency-reducing and amplitude-amplifying magnetic repulsion pendulum under a six-degree-of-freedom platform linear drive concerning each input parameter.
Figure 16. The output variation trend of RD-TENG is based on the frequency-reducing and amplitude-amplifying magnetic repulsion pendulum under a six-degree-of-freedom platform swinging drive concerning each input parameter.
Figure 17. Comparison of restoring forces for magnetic repulsion and traditional spring.
Figure 18. Charge dispatch and transfer schematic diagram for each electrode pair of RD-TENG. (a) 1-3 electrode pair. (b) 1-2 electrode pair. (c) 2-3 electrode pair. (d) rectified electrode pair.
Figure 19. (a) Simulation of the electric potential of the vibrating sheet moving from one end to the other in the RD-TENG. (b) Positions of each electrode and their electric potential variation curves as the vibrating sheet oscillates from one end to the other.
Figure 20. Three-electrode rectifier circuit diagram and its physical circuit image.
Figure 21. Vibration sheet style, parameters, model, and physical image.
Figure 22. Vibration simulation of the vibrating steel sheet.

Figure 23. Exploded isometric view and front view of the full-scale structural model of the RD-TENG with all components.
Figure 24. Packaged device model parameters and physical image.
Figure 25. Cross-sectional schematic and orientation diagram of a single structural cycle of the RD-TENG.
Figure 26. Fixing sheet parameters and physical image.
Figure 27. Acrylic ring gasket parameters, model, and physical image.
Figure 28. Structural details of push pin and latch alignment holes.
Figure 29. Push pin and latch parameters.
Figure 30. Bottom sealing plate and its parameters.
Figure 31. The protruding end of the push pin serves as the electrode lead-out terminal.
Figure 32. Electrode conductive sheet parameters, position, model, and physical image.
Figure 33. Packaging barrel parameters and its physical image.
Figure 34. The top sealing plate and its parameters.
Figure 35. Conductive sponge and its installation location.
Figure 36. Assembly steps of the RD-TENG device.
Figure 37. Parameters and physical image of the rotating-clasp device.
Figure 38. (a) Details and physical image of the RD-TENG device array. (b) Connection method of push-pin electrodes in the device array.
Figure 39. Vibrational energy simulation testing platform.
Figure 40. Comparison of the output of RD-TENG (Structure 4) with traditional structures. (a-c) Comparison of output charge, current, and voltage for the 1-2 electrode pair across different structures. (d-f) Comparison of output charge, current, and voltage for the 2-3 electrode pair across different structures. (g-h) Comparison of output charge, current, and voltage for the rectified electrode pair across different structures.
Figure 41. Output characteristics of RD-TENG. (a) Cumulative charge of the rectified electrode pair of RD-TENG under optimal excitation conditions. (b-c) Short-circuit current and open-circuit voltage of each electrode pair. (d) The relationship between charge output of each electrode pair and amplitude under a frequency of 2.4 Hz. (e) The output relationship between the output quantities of the 2-3 electrode pair and the amplitude under a frequency of 2.4 Hz.
Figure 42. Output characteristics of RD-TENG for harvesting vibrational energy. (a) Array device vibration energy simulation testing platform (b-c) Under the optimal excitation conditions of frequency 2.4 Hz and amplitude 70 mm, the output relationship of short-circuit current and open-circuit voltage with the increase in the number of device arrays. (d) The output relationship of the 1-2 electrode pair for three devices within the array under optimal excitation conditions concerning the variation of azimuth angle. (e-i) Vertical test platform and short-circuit transfer charge, open-circuit voltage, short-circuit current of each electrode pair in vertical testing. (j-l) Output voltage, current, and peak power density of RD-TENG with different load resistances for the 1-2, 2-3, and rectified electrode pairs.
Figure 43. Various circuit matching modules used in different tests, adaptable to specific application scenarios.
Figure 44. Application of RD-TENG for vibration energy harvesting. (a) One RD-TENG device

charges a 100 μF capacitor for 100 seconds, drives two parallel calculators with continuous power supply for 15 seconds. (b) Schematic diagram showing the lighting of 32 2 W LED bulbs from dim to bright by adding 1 to 4 devices in sequence in the array under the optimal combination of 2.4 Hz frequency and 70 mm amplitude. (c) RD-TENG illuminates 32 2 W LED bulbs under vertical drive. (d) The rectifier electrode pair of a single RD-TENG device is placed in the bicycle front basket, driving five 2 W commercial LED bulbs as a front light or warning light for the bicycle, providing safety warnings for night rides.

Figure 45. Dimensional parameters and schematic diagram of the components of frequency-reducing and amplitude-amplifying magnetic repulsion pendulum.

Figure 46. RD-TENG based on frequency-reducing and amplitude-amplifying magnetic repulsion pendulum driven by a six-degree-of-freedom platform. (a) linear drive. (b) swinging drive.

Figure 47. Amplitude amplification calculation diagram of frequency-reducing and amplitude-amplifying magnetic repulsion pendulum under linear and swinging drive. (a) linear drive. (b) swinging drive.

Figure 48. Output characteristics of RD-TENG based on frequency-reducing and amplitude-amplifying magnetic repulsion pendulum under linear drive and swing drive on a six-degree-of-freedom platform. (a-c) Short-circuit transferred charge, open-circuit voltage, and short-circuit current of each electrode pair under linear drive. (d-f) Short-circuit transferred charge, open-circuit voltage, and short-circuit current of each electrode pair under swinging drive. (g-h) Relationship between output quantities and frequency (Fixed at an amplitude of 70 mm), amplitude (Fixed at a frequency of 2.4 Hz) under linear drive. (i) Frequency reduction and amplitude amplification characteristics under linear drive. (j-k) Relationship between output quantities and frequency (Fixed at an amplitude 70 mm), amplitude (Fixed at a frequency of 2.4 Hz) under swinging drive. (l) Frequency reduction and amplitude amplification characteristics under swinging drive.

Figure 49. Force analysis diagram of the vibrating sheet under swinging drive, where a larger swing angle allows the rotor to open more effectively.

Figure 50. Comparison of 1-3 and rectified electrode pair lighting up 64 2 W LED bulbs in linear drive and swinging drive mode of RD-TENG based on the frequency-reducing and amplitude-amplifying magnetic repulsion pendulum.

Figure 51. Durability test of the 1-3 electrode pair of RD-TENG based on low-frequency and amplitude-amplifier of magnetic repulsive pendulum under linear excitation.

Figure 52. Diagram of the unbalanced gravity pendulum set on the edge of a buoyant base.

Figure 53. Wave amplitudes and frequencies generated by different modes in a wave pool.

Figure 54. Screenshot of meteorological and sea conditions at the beginning of data acquisition during real-sea testing.

Figure 55. Real-sea output performance of the 1-3 electrode pair, showing no structural failure during the 14-minute testing period.

Figure 56. Comparison of capacitive energy storage efficiency between real-sea condition and optimal condition on six-degree-of-freedom simulation platform.

Figure 57. Comparison of output data under real-sea conditions and laboratory wave tank conditions mode 1

Supplementary Table 1. Comparison of RD-TENG output with traditional FCS structures.

Comparison	Frequency (Hz)	1-3 electrode pair			1-2 electrode pair			2-3 electrode pair			rectified electrode pair.		
		Voltage (V)	Current (μA)	Charge (nC)	Voltage (V)	Current (μA)	Charge (nC)	Voltage (V)	Current (μA)	Charge (nC)	Voltage (V)	Current (μA)	Charge (nC)
Structure 1	0.8	27	0.51	10	39	0.72	10	41	0.58	9	22	1.18	167
	1.2	84	4.46	73	236	13.44	117	196	8.12	88	146	20.14	668
	1.6	272	22.13	137	492	60.65	325	364	34.68	315	256	66.04	1820
	2	440	26.58	150	592	78.63	402	424	76.41	396	328	77.86	2388
	2.4	480	31.92	164	760	83.23	488	792	71.64	378	648	81.75	3058
Structure 2	0.8	63	0.35	10	84	1.34	15	56	1.24	11	44	1.63	271
	1.2	204	3.27	46	96	2.46	30	292	7.74	85	304	22.2	1357
	1.6	416	16.92	168	584	36.14	285	460	63.4	424	448	34	1645
	2	900	22.62	179	640	54.1	316	700	100.46	597	576	71.06	2285
	2.4	1240	30.76	191	720	93.09	523	860	104.93	570	780	107.6	3254
Structure 3	0.8	272	2.75	71	/	/	/	/	/	/	/	/	/
	1.2	544	5.91	144	/	/	/	/	/	/	/	/	/
	1.6	1100	14.43	271	/	/	/	/	/	/	/	/	/
	2	1400	20	284	/	/	/	/	/	/	/	/	/
	2.4	1640	29.79	303	/	/	/	/	/	/	/	/	/
RD-TENG	0.8	264	2.91	77	180	3.14	78	116	2.75	73	124	2.37	433
	1.2	920	32.92	262	560	40.26	227	496	41.37	576.89	660	32.11	1458.4
	1.6	1420	70.22	951	740	96.43	1080	576	115.41	1215.24	840	77.41	2433.8
	2	1700	80.14	1326	940	144.92	1552	960	254.48	2643.62	960	118.2	3226
	2.4	2200	94	1903	1360	182	2595	1220	314.69	3275.2	1080	272	5480

Supplementary Note 1. The difficulties arise after the densification of friction layers in traditional FCS-TENG.

1. Charge cancellation issue.

As shown in Supplementary Figure 1, In the traditional free-standing layer vertical contact-separation mode TENG (FCS-TENG), when the array is stacked beyond two structural cycles, all the intermediate fixed electrodes are shared—except for the first and last fixing sheets—leading to severe charge cancellation issues, For the fixing sheet 3, when the mover on the vibrating sheet 2 approaches it, the mover on the vibrating sheet 4 moves away, and vice versa. This means that at any given moment, the fixing sheet 3 always has one mover approaching and one mover moving away.

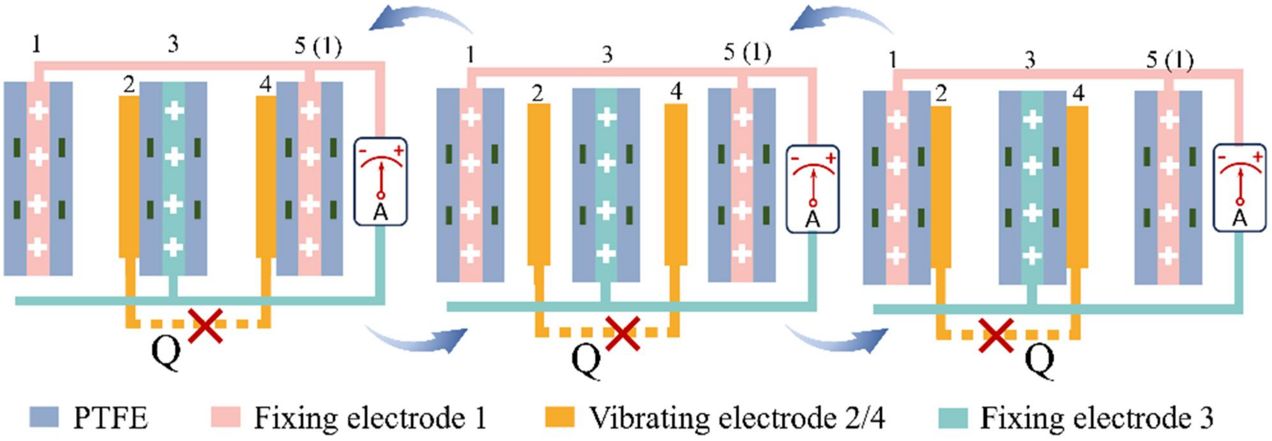

Supplementary Figure 1. Charge cancellation phenomenon caused by shared electrodes in stacked FCS models (the electrode is grounded).

However, because there is no mechanism for charge compensation and migration between the movers on vibrating sheets 2 and 4, caused by the completely symmetric film coating and arrangement in the dense stacking of fixing sheets 1 and 3, the electric potential between movers 2 and 4 remains unchanged. As a result, the electric potential on fixing sheet 3 also remains almost constant, and the same applies to other fixing sheets. In summary, the absence of a charge migration pathway between movers 2 and 4 prevents the creation of a high potential difference between the output channels of layers 1 and 3, resulting in minimal charge transfer between electrodes 1 and 3 and almost no current flow in the circuit.

2. Electrostatic shielding issue.

The performance degradation of traditional single-electrode mode TENGs (SE-TENGs) primarily stems from the electrostatic shielding effect of the primary electrode.[31] The amount of short-circuit

transferred charge Q_{sc} slowly approaches its saturation value, which is only half that of contact-separation TENG (CS-TENG) or free-standing slide TENG (FS-TENG). When the free-moving layer separates from the electrode, the triboelectric charges are evenly distributed between the single electrode and the reference (or ground) electrode, causing both sides to maintain the same electric potential. This limits the maximum energy conversion efficiency to merely 50%.[31] In conventional dense-layer FCS-TENGs, we designed three representative models (Supplementary Figure 2): Structure 1 (vibrating sheet fully coated, fixing sheet uncoated), Structure 2 (vibrating sheet uncoated, fixing sheet fully coated), and Structure 3 (cross-coated, without short-circuiting between vibrating sheets). All three models can be analytically decomposed into superimposed SE-TENG units; taking Structure 3 as an example, its decomposition is illustrated in Supplementary Figure 3. Structures 1 and 2 follow the same principle. From this analysis, we find that in all three models, each independent layer unit can be split into two SE-TENGs, confirming that their basic functional units are inherently SE configurations rather than vertical CS types. Due to the intrinsic electrostatic shielding effect of the SE mode, these FCS dense-stacked models inherently suffer from internal shielding, especially in high-density multilayer configurations. As the layer density increases, the separation distance x of the free-moving layer decreases, enhancing the influence of the triboelectric field on adjacent electrodes. Consequently, the electrostatic shielding effect becomes increasingly significant, further constraining the output performance.

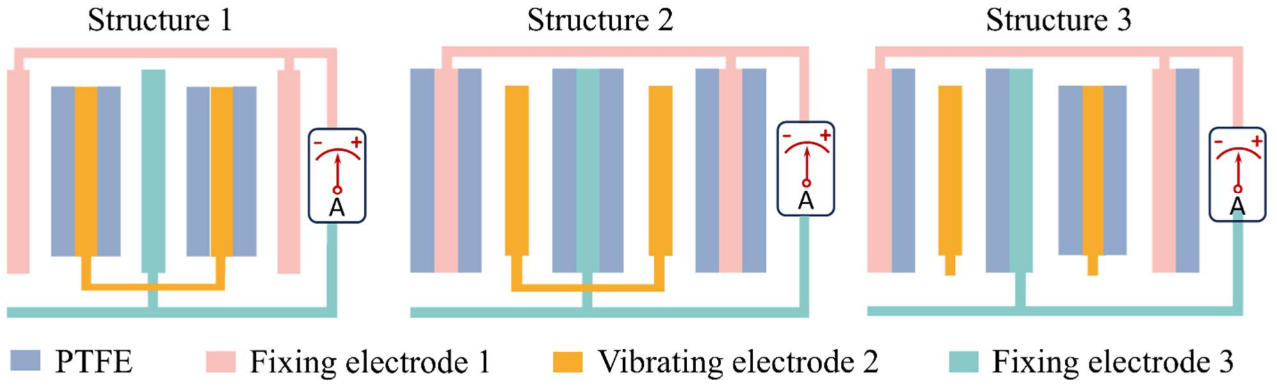

Supplementary Figure 2. Three traditional FCS models were designed and constructed in this work.

3. Low contact-separation efficiency issue.

In high-density stacked TENGs, the motion space of each individual layer is limited, which restricts the full contact and separation behavior during vibration. This results in reduced charge generation and transfer efficiency. As the stack becomes denser, the available displacement decreases, leading to weaker contact forces and incomplete separation—ultimately lowering the overall output performance.

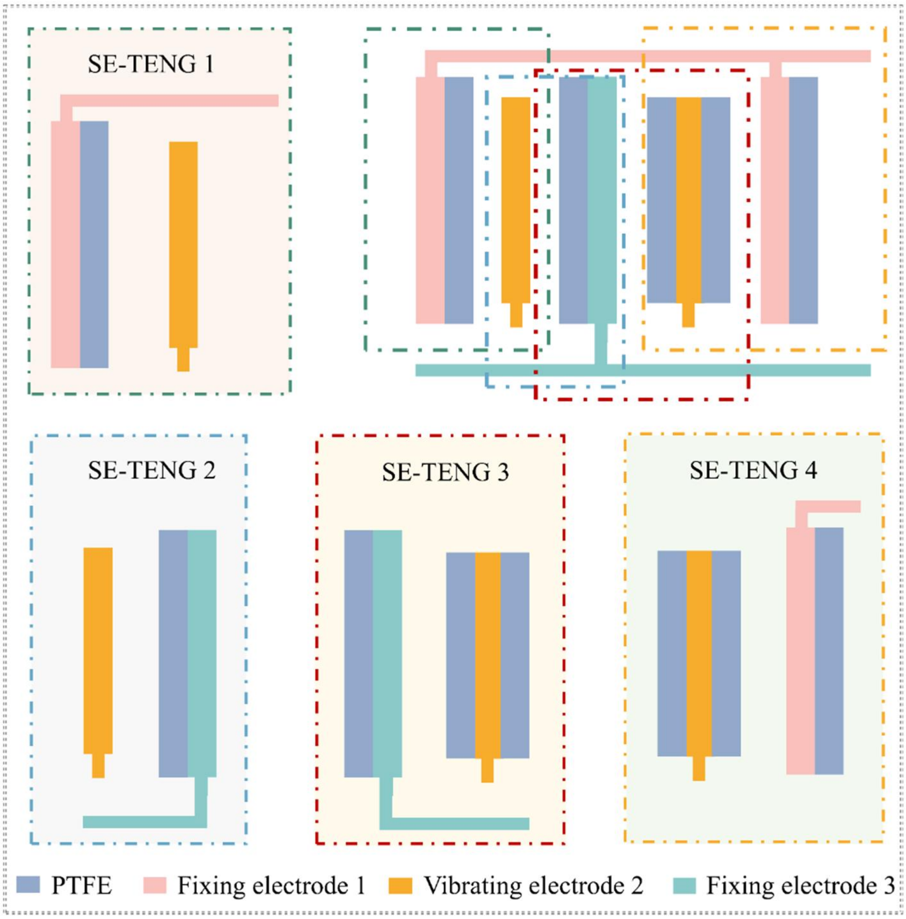

Supplementary Figure 3. Schematic decomposition of the basic unit in the traditional FCS model.

Supplementary Note 2. Inspiration from the rattle drum to the prototype device.

As illustrated in Supplementary Figure 4a and b, the prototype TENG device is inspired by the mechanical and dynamical features of a traditional rattle drum. During the shaking process, the pendulums on both sides maintain conservation of angular momentum and tension equilibrium, swing in the same direction, ultimately generating sound waves through impacts with the drum surface. Structurally, the design borrows the combined rigid-flexible architecture of the drum body and the cycloidal motion of its pendulums, forming a layered configuration of fixed electrode–short-circuited vibrating electrode–fixed electrode (Supplementary Figure 4c). In terms of dynamic response, elastic vibrating sheets are introduced to emulate the synchronous striking motion of the pendulums, achieving dual-sided symmetric triboelectric excitation and charge transfer. Electrically, a central short-circuit pathway is innovatively incorporated to enable dynamic redistribution of charges across dual loops, promoting field equilibrium and output balance. This also enables the middle electrode to participate actively in the energy harvesting process, thereby transforming the conventional single-electrode unit into a vertical contact-separation mode. As a result, the inherent electrostatic shielding effect of single-electrode structures—especially problematic in high-density stacks—is significantly mitigated. The charge transport topology is thus optimized, enhancing the overall output. This design exemplifies a cross-domain transition from mechanical inspiration to electrodynamic coupling, offering a new paradigm for integrated structural and mechanistic design in TENG systems.

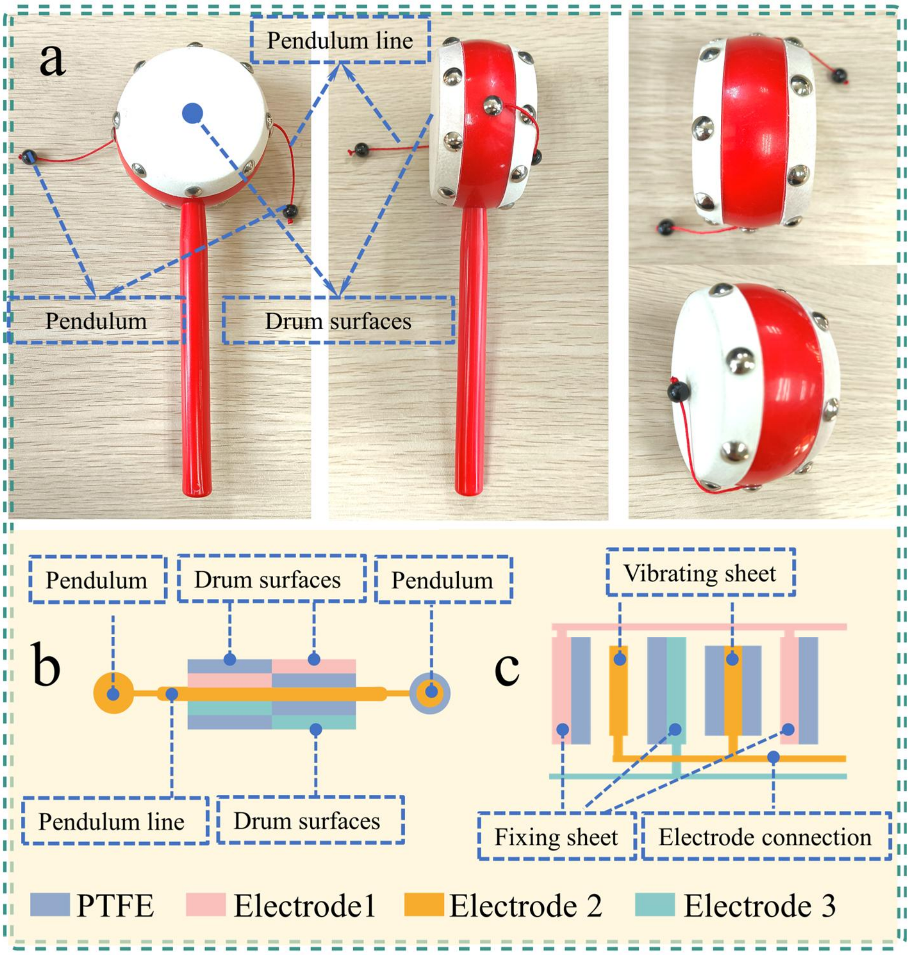

Supplementary Figure 4. (a) Rattle-drum. (b) rattle-drum-type TENG. (c) RD-TENG

Supplementary Note 3. From the rattle-drum-model TENG to the RD-TENG.

Supplementary Figure 5a and b illustrate the correspondence of motion states throughout a full operating cycle from the rattle-drum-model prototype TENG model (a) to the RD-TENG model (b). At multiple levels—including structural prototype, vibration mode, mechanical response, and charge path design—the RD-TENG deeply draws inspiration from the rattle-drum mechanism, particularly its characteristics of symmetric excitation, periodic impact, and multi-channel output. Based on this, a three-electrode RD-TENG dynamic charge dispatch mechanism system is developed, featuring a short-circuited dual-state vibrating sheet, dual output pathways. Constructs internal and external charge circulation paths via a dual-TENG configuration and alternate film coatings. The arrangement of the three electrodes on the device is shown in Supplementary Figure 6.

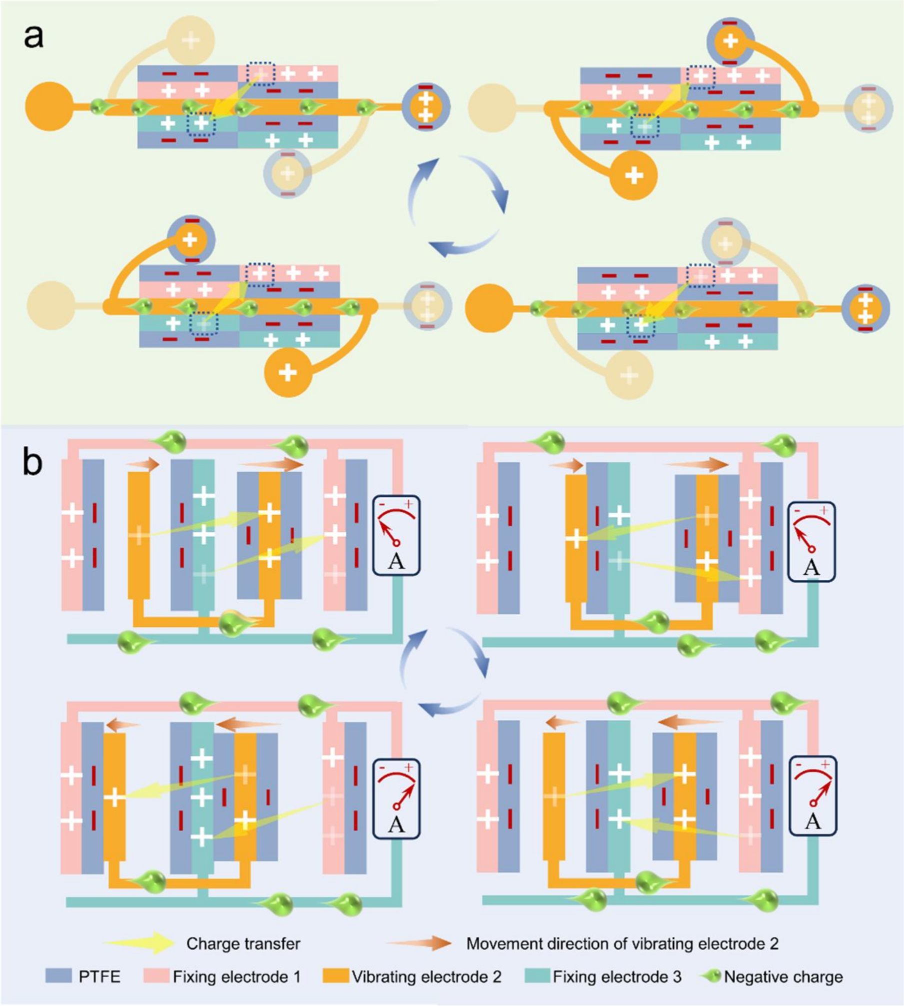

Supplementary Figure 5. State correspondence diagram from the rattle-drum-type prototype TENG model (a) to the RD-TENG model (b). The electrode is grounded.

Based on the principle of the rattle-drum-type charge dispatch strategy, we optimized the film-coating method for electrodes (Supplementary Figure 5b and Supplementary Figure 6): single-sided

film coating on the fixed sheets, non-coated vibrating sheets, and full-coated vibrating sheets. These are arranged in the following order (from left to right): right-coated fixed sheet, non-coated vibrating sheet, left-coated fixed sheet, full-coated vibrating sheet, and right-coated fixed sheet (with another structure cycle starting), and sequence forms three electrodes with alternating coatings. Electrode 1 consists entirely of fixed sheets with right-side coatings, serving as a primary charge collection component. Electrode 2 is composed of two types of vibrating sheets—non-coated and fully coated—which are electrically short-circuited together. These vibrating sheets function similarly to the two pendulums in a rattle drum, facilitating charge redistribution during operation. Electrode 3 is made up of fixed sheets with left-side coatings, acting as another key charge collection component. Notably, the fixed sheets in electrodes 1 and 3 correspond to the two opposite sides of a rattle drum, further reinforcing the mechanical-electrical analogy embedded in the RD-TENG design.

As illustrated in Supplementary Figure 5b, the charge transfer mechanism of the RD-TENG model is. On the microscopic level, the internal short-circuit electrode's charge circulation promotes the external electrode's charge outer circulation. The charge transfer in the internal loop formed by the short-circuit TENG electrode pair is always opposite to and does not interfere with the external loop, eliminating the charge cancellation caused by reverse movement along the same path due to shared electrodes between output electrodes. On the macroscopic level, the introduction of internal loop electrodes ensures that every neighboring fixed electrode of the external electrodes is a short-circuit electrode (vibrating electrode 2). In a vibration cycle, the charge transfer of the short-circuit electrode undergoes two cycles, making each external electrode no longer behave as a single-electrode mode but as a contact-separation mode. In other words, a potential electrode pair is established between the adjacent short-circuited electrode and the fixed electrode.

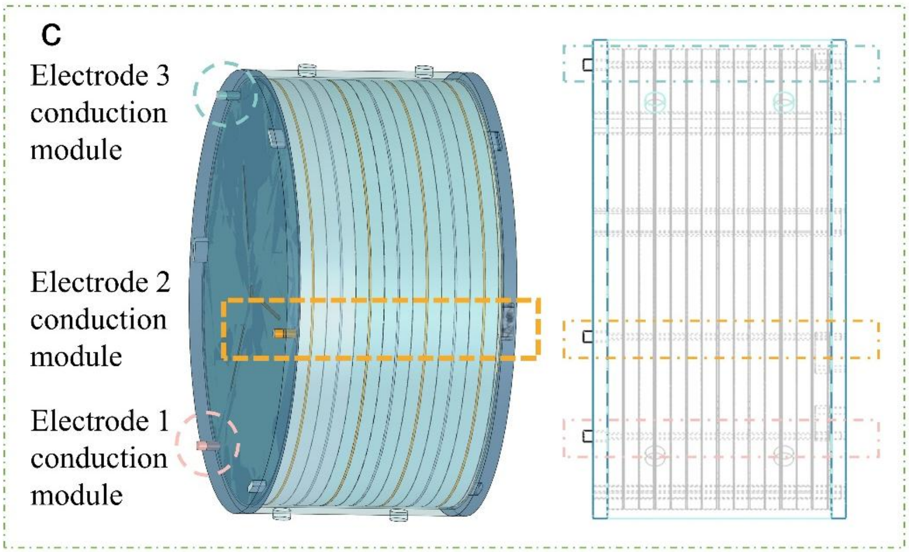

Supplementary Figure 6. Three-electrode configuration of the RD-TENG device

Supplementary Note 4. Essential differences between the three-electrode RD-TENG and previous works.

In prior literature, most TENGs described as “three-electrode structures” typically feature a third electrode functioning as an auxiliary signal extraction layer or sensing terminal, primarily aimed at enhancing directional sensitivity, expanding the detection area, or increasing the number of output channels. However, although such designs appear to incorporate three electrodes geometrically, they do not establish a functional mechanism for charge path reconstruction. Their decomposed fundamental units remain dominated by two-electrode configurations, lacking an intrinsically coupled charge dispatch framework (see comparison in Supplementary Table 2). In contrast, the three-electrode charge dispatch structure proposed in this work introduces a short-circuited intermediate electrode (Electrode 2) composed of vibrating sheets into the conventional two-electrode TENG system. Rather than serving as a passive signal layer, this intermediate electrode forms an active internal circulation channel for dynamic charge transfer. Coupled with the alternate film coating strategy, the system collectively forms two external output paths from the fixed electrodes and one internal charge guidance path governed by the short-circuited electrode, operating in a coordinated manner. Under this architecture, the multi-layer structure no longer behaves as a simple superposition of independent layers. Instead, it evolves into a synergistic charge dispatch network with internal diversion and external enhancement. During the contact-separation cycle, the short-circuited electrode actively guides the charge flow and redistribution, effectively mitigating charge cancellation caused by shared electrodes and electrostatic shielding effects inherent in single-electrode elementary unit configurations. This significantly boosts the overall charge output and structural integration potential of the system. Therefore, from both the device’s mechanical motion mode and the electrode architecture perspective, the RD-TENG represents a fundamentally new configuration or model of TENG.

Supplementary Table 2. Comparison between previously reported three-electrode configurations and the approach presented in this work

No.	Yr.	newly illustrated schematic	Mode	Function of the Three-Electrode Configuration	Essence	Significance	Assessment	Re.
1	2018	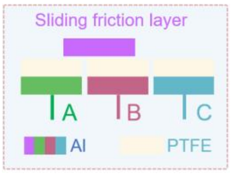	FS	Enhances energy harvesting density and angle coverage	2-Elect.	Alternating arrangement forms electrodes A, B, and C to collect energy from multiple directions	Merely a geometric addition of electrodes	51
2	2023	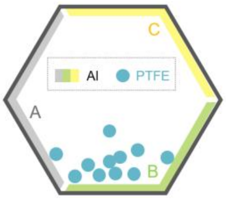	SE	Enhances energy harvesting density and angle coverage	1-Elect.	Three independent electrode groups (A, B, C) are formed by dividing the inner aluminum foils symmetrically at 120°, connected in a three-phase circuit	A geometric partitioning to collect outputs from different orientations simultaneously	52
3	2023	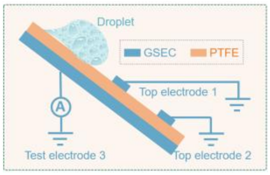	SL	Functions like a grating electrode for electrical signal monitoring	2-Elect.	Uses time delays between multiple electrodes to monitor droplet velocity, offering an integrated solution for fluid energy harvesting and intelligent sensing	Adds electrodes in-plane to establish signal–space relationships	53
4	2023	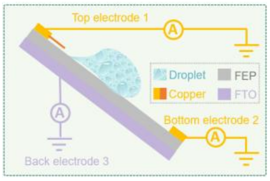	SL	Collects positive and negative charges within droplets	2-Elect.	The top and bottom electrodes separately collect different polar charges; the rear electrode is grounded to improve charge extraction efficiency and output stability compared to conventional droplet-based TENGs	Uses spatially distributed electrodes to extract bipolar charges from droplets	54
6	This work	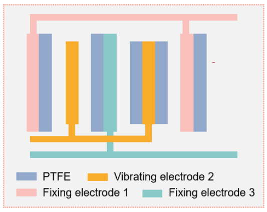 RD-TENG	Stacked FCS	Enables charge dispatch, mode conversion, and output participation	3-Elect.	The three-electrode design constructs dual-loop circuits to mitigate charge cancellation caused by shared electrodes in conventional models, and uses a short-circuit channel to convert traditional CE-type units into CS-type units, addressing intrinsic electrostatic shielding problems	The internal electrode actively participates in both charge dispatch and output, with an irreplaceable role beyond mere geometric addition	This work

Caption: The meanings of the relevant abbreviations are as follows:
SL: freestanding sliding mode;
SE: single-electrode mode;
SL: solid–liquid contact mode;
Stacked FCS: freestanding contact-separation high-density stacked mode.
Elect.: electrode

Supplementary Note 5. Quantitative theoretical model.

1. Strategy

Specific pathways by which the charge dispatch strategy and structural innovation strategies overcome the limitations of traditional models can be summarized as follows:

1) Path diversion: By constructing dual-loop circuits, the charge dispatch strategy mitigates charge cancellation issues. Coordinated output between the internal short-circuited TENG and the external TENG forms an internal-external charge loop system, ensuring charge consistency within each loop throughout all operating states and enabling effective charge collection.

2) Mode transformation: The strategy overcomes inherent electrostatic shielding by converting the basic working unit from a single-electrode (SE) mode to a vertical contact-separation (CS) mode. This is achieved by configuring the free layer into two alternating states connected via a short-circuit and implementing an alternate film coating design, thus eliminating the electrostatic shielding intrinsic to SE modes (see Supplementary Figure 3 and Supplementary Figure 7).

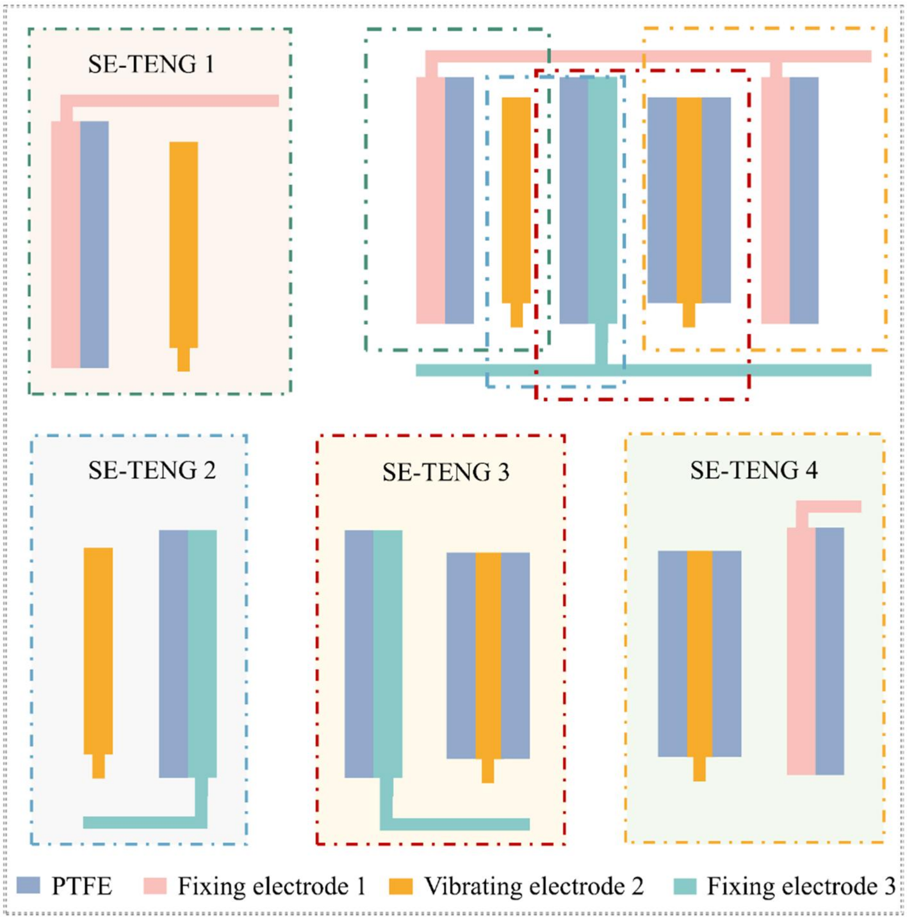

Supplementary Note 1 Supplementary Figure 3. Schematic decomposition of the basic unit in the traditional FCS model.

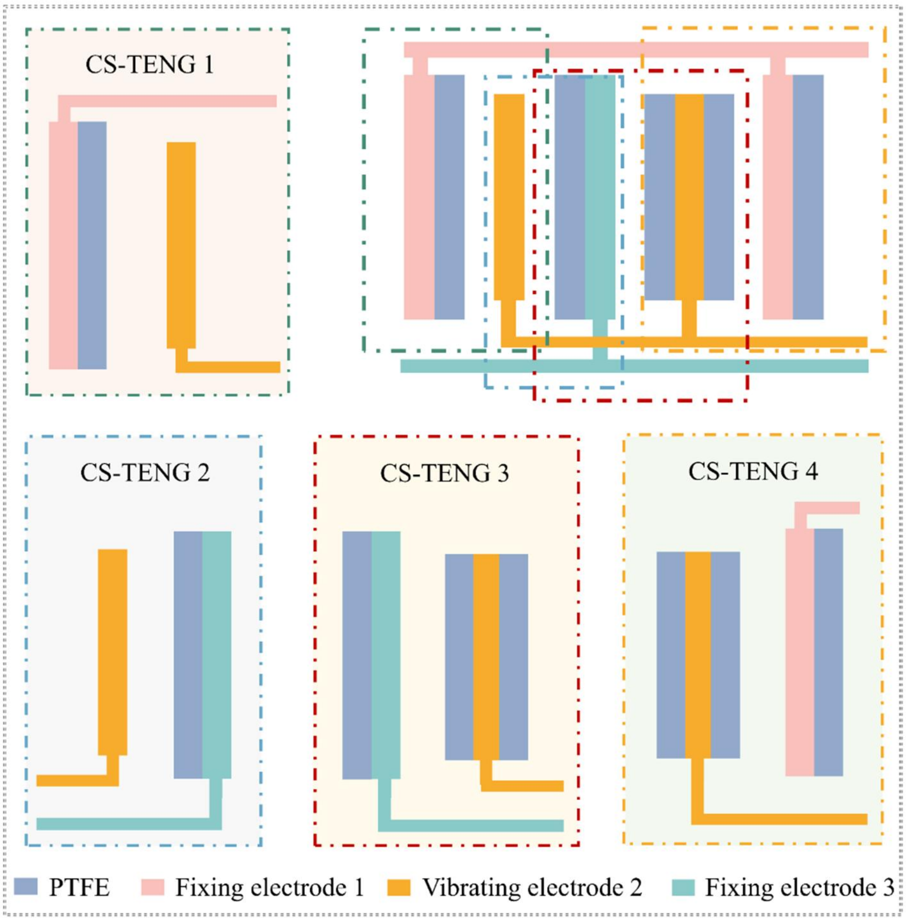

Supplementary Figure 7. Decomposition diagram of the fundamental unit of the RD-TENG model

3) Engineering innovations: Through the subsequent structural optimization strategies—such as harmonic-oscillator-type vibrating sheet design, contact push pin interconnects, and looping insertion stacking methods—the working environment of the free layer is enhanced, leading to improved contact-separation efficiency and overall energy harvesting performance.

Supplementary Figure 8 illustrates the equivalent capacitive circuit networks for three traditional models with and without the charge dispatch strategy (Supplementary Figure 8a–b) and for the RD-TENG (Supplementary Figure 8c–d). In Supplementary Figure 8d, the short-circuit connection at nodes II–II constructs a dedicated charge dispatch pathway. Through this pathway, the fundamental unit of the RD-TENG's equivalent circuit is transformed into a vertical contact-separation mode. In contrast, traditional architectures, lacking this internal channel, remain in the single-electrode mode, which inherently suffers from strong electrostatic shielding effects. Moreover, the introduction of this short-circuit pathway optimizes the capacitive coupling by avoiding the simple additive superposition of series and parallel capacitances. In RD-TENG, the capacitive interaction becomes more synergistic, while in conventional models, capacitors connected in series or parallel often lead to mutual cancellation, especially due to the shared electrode configuration. This directly

corresponds to the charge neutralization issue observed in traditional stacked structures. The analytical equations governing this behavior are detailed in the following section.

2. For traditional configurations (applicable to Structures 1, 2, and 3):

As shown in the equivalent capacitive circuit networks in Supplementary Figure 8a–b, when nodes I–IV are short-circuited, the upper loop can be derived using Kirchhoff's Voltage Law (KVL) as follows:

$$-\frac{Q_4}{C_4}+\frac{Q_{D4}}{C_D}-\frac{Q_{D3}}{C_D}+\frac{Q_3}{C_3}=0 \tag{1}$$

Lower loop:

$$-\frac{Q_{D2}}{C_D}+\frac{Q_2}{C_2}-\frac{Q_1}{C_1}+\frac{Q_{D1}}{C_D}=0 \tag{2}$$

We define the target physical quantities as follows: C_D represents the capacitance contributed by dielectric layers (i.e., parasitic capacitance); C_1 , C_2 , C_3 , C_4 represents the air gap capacitance introduced between different electrode plates (as illustrated in the Supplementary Figure 8b); Q_1, Q_2, Q_3, Q_4 denotes the charge stored on dielectric layer C_1, C_2, C_3, C_4 , Q_{D1} refers to the charge on the left-side capacitor C_D of element C_1; Q_{D2} refers to the charge on the right-side capacitor C_D of element C_2; Q_{D3} refers to the charge on the right-side capacitor C_D of element C_3; Q_{D4} refers to the charge on the left-side capacitor C_D of element C_4;.σ_T is the surface charge density on the dielectric layer; S stands for the area of each electrode plate. According to charge conservation at Node 1:

$$-\sigma_T S=-Q_1-Q_{D1} \tag{3}$$

$$Q_{D1}=\sigma_T S-Q_1 \tag{4}$$

Charge conservation at Node 2:

$$-\sigma_T S=-Q_2-Q_{D2} \tag{5}$$

$$Q_{D2}=\sigma_T S-Q_2 \tag{6}$$

Charge conservation at Node 3:

$$-\sigma_T S=-Q_3-Q_{D3} \tag{7}$$

$$Q_{D3}=\sigma_T S-Q_3 \tag{8}$$

Charge conservation at Node 2:

$$-\sigma_T S=-Q_4-Q_{D4} \tag{9}$$

$$Q_{D4}=\sigma_T S-Q_4 \tag{10}$$

According to Kirchhoff's current law (KCL), charge conservation at terminals I can be expressed as:

$$\sigma_T S\text{-}Q=Q_4+Q_{D1} \tag{11}$$

Substituting equation (4) into equation (11) yields:

$$\sigma_T S\text{-}Q=Q_4+\sigma_T S\text{-}Q_1 \tag{12}$$

$$\text{-}Q=Q_4\text{-}Q_1 \tag{13}$$

Charge conservation at terminals II:

$$2\sigma_T S=Q_{D4}+Q_{D3} \tag{14}$$

Substituting equations (4) and (10) into equation (14) yields:

$$2\sigma_T S=\sigma_T S\text{-}Q_4+\sigma_T S\text{-}Q_3 \tag{15}$$

$$Q_4=\text{-}Q_3 \tag{16}$$

Charge conservation at terminals III:

$$\sigma_T S+Q=Q_3+Q_{D2} \tag{17}$$

Substituting equation (6) into equation (17) yields:

$$\sigma_T S+Q=Q_3+\sigma_T S\text{-}Q_2 \tag{18}$$

$$Q=Q_3\text{-}Q_2 \tag{19}$$

Charge conservation at terminals IV:

$$0=Q_1+Q_2 \tag{20}$$

$$Q_1=\text{-}Q_2 \tag{21}$$

By combining the charge conservation equations at nodes 1, 2, 3, and 4, along with those at terminals I, II, III, and IV, the target physical quantities can ultimately be determined.

$$Q_1=0 \tag{22}$$

$$Q_2=0 \tag{23}$$

$$Q_3=Q \tag{24}$$

$$Q_4=\text{-}Q \tag{25}$$

$$Q_{D1}=\sigma_T S \tag{26}$$

$$Q_{D2}=\sigma_T S \tag{27}$$

$$Q_{D3}=\sigma_T S\text{-}Q \tag{28}$$

$$Q_{D4}=\sigma_T S+Q \tag{29}$$

Substituting the derived physical quantities into equation (1) yields:

$$\frac{Q}{C_4}+\frac{\sigma_T S+Q}{C_D}\text{-}\frac{\sigma_T S\text{-}Q}{C_D}+\frac{Q}{C_3}=0 \tag{30}$$

$$\text{-}\frac{\sigma_T S}{C_D}+\frac{\sigma_T S}{C_D}=0 \tag{31}$$

The final solution yields the transferred charge between ports I and III, i.e., between electrode pair 1–3, as:

$$Q=0 \tag{32}$$

Which further yields:

$$V=0 \tag{33}$$

The results indicate that no charge transfer occurs in the traditional models, resulting in zero output. This aligns with our finite element simulation analysis of the three traditional models (Supplementary Figure 2 in Supplementary Note 1 and Figure 2b). In experimental validation, however, a weak output was observed in all three traditional models (Figure 2c–e), with transferred charge on the order of several hundred nanocoulombs. This discrepancy can be attributed to deviations between the idealized conditions assumed in the models and the real-world laboratory environment and measurement process. Such deviations include: edge effects arising from the use of non-infinite electrodes versus the idealized infinite parallel plate model; humidity effects compared to ideal vacuum conditions; and non-synchronized contact-separation of the vibrating and fixed layers versus perfect coordination. These factors can introduce surface charge deviations and, under unbalanced electric fields, result in minor charge transfer. Nevertheless, the magnitude remains within the error range, and the model's results are consistent with theoretical charge transfer principles, simulation predictions, and experimental observations.

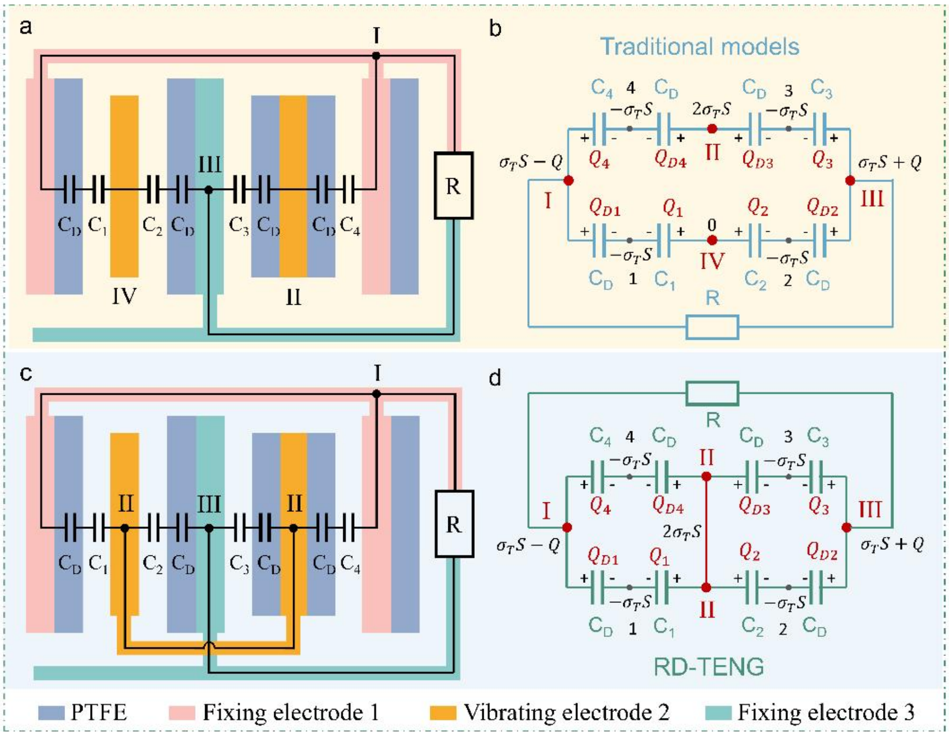

Supplementary Figure 8. Equivalent capacitive circuit network models of traditional configurations and the RD-TENG configuration.

3. For the RD-TENG configuration:

As shown in the equivalent capacitive circuit network in Supplementary Figure 8c-d, all parameter definitions remain consistent with those previously stated. According to KVL, for the loop II-4-I-1-II, we have:

$$-\frac{Q_4}{C_4}+\frac{Q_{D4}}{C_D}-\frac{Q_1}{C_1}+\frac{Q_{D1}}{C_D}=0 \tag{34}$$

For the loop II-II-2-III-3, we obtain:

$$-\frac{Q_{D3}}{C_D}+\frac{Q_3}{C_3}-\frac{Q_{D2}}{C_D}+\frac{Q_2}{C_2}=0 \tag{35}$$

When I-III are short-circuited, applying KVL to the loop II-4-I-III-3 gives:

$$-\frac{Q_4}{C_4}+\frac{Q_{D4}}{C_D}-\frac{Q_{D3}}{C_D}+\frac{Q_3}{C_3}=0 \tag{36}$$

According to KCL at different nodes, the charge conservation at Node 1 is:

$$-\sigma_T S=-Q_1-Q_{D1} \tag{37}$$

$$Q_{D1}=\sigma_T S-Q_1 \tag{38}$$

Charge conservation at Node 2:

$$-\sigma_T S=-Q_2-Q_{D2} \tag{39}$$

$$Q_{D2}=\sigma_T S-Q_2 \tag{40}$$

Charge conservation at Node 3:

$$-\sigma_T S=-Q_3-Q_{D3} \tag{41}$$

$$Q_{D3}=\sigma_T S-Q_3 \tag{42}$$

Charge conservation at Node 3:

$$-\sigma_T S=-Q_4-Q_{D4} \tag{43}$$

$$Q_{D4}=\sigma_T S-Q_4 \tag{44}$$

Charge conservation at terminals I:

$$\sigma_T S-Q=Q_4+Q_{D1} \tag{45}$$

Substituting equation (38) into equation (45) yields:

$$\sigma_T S-Q=Q_4+\sigma_T S-Q_1 \tag{46}$$

$$Q_1=Q_4+Q \tag{47}$$

Charge conservation at terminals II:

$$2\sigma_T S=Q_{D4}+Q_{D3}+\mathrm{Q}_1+\mathrm{Q}_2 \tag{48}$$

Substituting equations (42) and (44) into equation (48) yields:

$$2\sigma_T S=\sigma_T S-Q_4+\sigma_T S-Q_3+\mathrm{Q}_1+\mathrm{Q}_2 \tag{49}$$

$$Q_4+Q_3=\mathrm{Q}_1+\mathrm{Q}_2 \tag{50}$$

Charge conservation at terminals III:

$$\sigma_T S+Q=Q_3+Q_{D2} \tag{51}$$

Substituting equation (40) into equation (51) yields:

$$\sigma_T S+Q=Q_3+\sigma_T S\text{-}Q_2 \tag{52}$$

$$Q_3=Q_2+Q \tag{53}$$

Substituting the results from the combined charge conservation equations at nodes 1, 2, 3, 4 and at the three terminals into the three KVL equations, and then substituting into equation (34), we obtain:

$$-\frac{Q_4}{C_4}+\frac{\sigma_T S\text{-}Q_4}{C_D}=\frac{Q_4+Q}{C_1}-\frac{\sigma_T S\text{-}(Q_4+Q)}{C_D} \tag{54}$$

Substituting into equation (35), we obtain:

$$-\frac{\sigma_T S\text{-}\left(Q_2+Q\right)}{C_D}+\frac{Q_2+Q}{C_3}=\frac{\sigma_T S\text{-}Q_2}{C_D}-\frac{Q_2}{C_2} \tag{55}$$

Substituting into equation (36), we obtain:

$$-\frac{Q_4}{C_4}+\frac{\sigma_T S\text{-}Q_4}{C_D}-\frac{\sigma_T S\text{-}\left(Q_2+Q\right)}{C_D}+\frac{Q_2+Q}{C_3}=0 \tag{56}$$

By combining the three simplified KVL equations, we obtain:

$$Q_4=\frac{\frac{2\sigma_T S}{C_D}\text{-}Q\left(\frac{1}{C_D}+\frac{1}{C_1}\right)}{\frac{1}{C_2}+\frac{1}{C_3}+\frac{2}{C_D}} \tag{57}$$

$$Q_2=\frac{\frac{2\sigma_T S}{C_D}\text{-}Q\left(\frac{1}{C_D}+\frac{1}{C_3}\right)}{\frac{1}{C_2}+\frac{1}{C_3}+\frac{2}{C_D}} \tag{58}$$

$$-Q_4\left(\frac{1}{C_4}+\frac{1}{C_D}\right)+Q_2\left(\frac{1}{C_D}+\frac{1}{C_3}\right)+Q\left(\frac{1}{C_D}+\frac{1}{C_3}\right)=0 \tag{59}$$

$$-\frac{\frac{2\sigma_T S}{C_D}\text{-}Q\left(\frac{1}{C_D}+\frac{1}{C_1}\right)}{\frac{1}{C_1}+\frac{1}{C_4}+\frac{2}{C_D}}\left(\frac{1}{C_4}+\frac{1}{C_D}\right)+\frac{\frac{2\sigma_T S}{C_D}\text{-}Q\left(\frac{1}{C_D}+\frac{1}{C_3}\right)}{\frac{1}{C_2}+\frac{1}{C_3}+\frac{2}{C_D}}\left(\frac{1}{C_D}+\frac{1}{C_3}\right)+Q\left(\frac{1}{C_D}+\frac{1}{C_3}\right)=0 \tag{60}$$

$$\frac{\left(\frac{\frac{2\sigma_T S}{C_D}}{\frac{1}{C_1}+\frac{1}{C_4}+\frac{2}{C_D}}\left(\frac{1}{C_4}+\frac{1}{C_D}\right)-\frac{\frac{2\sigma_T S}{C_D}}{\frac{1}{C_2}+\frac{1}{C_3}+\frac{2}{C_D}}\left(\frac{1}{C_D}+\frac{1}{C_3}\right)\right)}{\left(\frac{\left(\frac{1}{C_D}+\frac{1}{C_1}\right)\left(\frac{1}{C_4}+\frac{1}{C_D}\right)}{\frac{1}{C_1}+\frac{1}{C_4}+\frac{2}{C_D}}-\frac{\left(\frac{1}{C_D}+\frac{1}{C_3}\right)\left(\frac{1}{C_D}+\frac{1}{C_3}\right)}{\frac{1}{C_2}+\frac{1}{C_3}+\frac{2}{C_D}}+\left(\frac{1}{C_D}+\frac{1}{C_3}\right)\right)}=Q \tag{61}$$

$$\frac{\frac{2\sigma_T S}{C_D}\left(\frac{1}{C_4}+\frac{1}{C_D}\right)\left(\frac{1}{C_2}+\frac{1}{C_3}+\frac{2}{C_D}\right)-\frac{2\sigma_T S}{C_D}\left(\frac{1}{C_D}+\frac{1}{C_3}\right)\left(\frac{1}{C_1}+\frac{1}{C_4}+\frac{2}{C_D}\right)}{\left(\frac{1}{C_D}+\frac{1}{C_1}\right)\left(\frac{1}{C_4}+\frac{1}{C_D}\right)\left(\frac{1}{C_2}+\frac{1}{C_3}+\frac{2}{C_D}\right)-\left(\frac{1}{C_D}+\frac{1}{C_3}\right)\left(\frac{1}{C_D}+\frac{1}{C_3}\right)\left(\frac{1}{C_1}+\frac{1}{C_4}+\frac{2}{C_D}\right)+\left(\frac{1}{C_2}+\frac{1}{C_3}+\frac{2}{C_D}\right)\left(\frac{1}{C_1}+\frac{1}{C_4}+\frac{2}{C_D}\right)\left(\frac{1}{C_D}+\frac{1}{C_3}\right)}=Q \tag{62}$$

The final simplified expression is:

$$\frac{\frac{2\sigma_T S}{C_D}\left(\frac{1}{C_4}+\frac{1}{C_D}\right)\left(\frac{1}{C_2}+\frac{1}{C_3}+\frac{2}{C_D}\right)-\frac{2\sigma_T S}{C_D}\left(\frac{1}{C_D}+\frac{1}{C_3}\right)\left(\frac{1}{C_1}+\frac{1}{C_4}+\frac{2}{C_D}\right)}{\left(\frac{1}{C_D}+\frac{1}{C_1}\right)\left(\frac{1}{C_4}+\frac{1}{C_D}\right)\left(\frac{1}{C_2}+\frac{1}{C_3}+\frac{2}{C_D}\right)+\left(\frac{1}{C_2}+\frac{1}{C_D}\right)\left(\frac{1}{C_1}+\frac{1}{C_4}+\frac{2}{C_D}\right)\left(\frac{1}{C_D}+\frac{1}{C_3}\right)}=Q \tag{63}$$

To further simplify, we observe that during the motion, $C_1=C_3$, $C_2=C_4$, We define $C_1=C_3=C_x$, $C_2=C_4=C_{s\text{-}x}$, where, based on the parallel-plate capacitor model $C=\frac{\varepsilon S}{x}$, we obtain:

$$C_x=\frac{\varepsilon_0 S}{x} \tag{64}$$

$$C_{s\text{-}x}=\frac{\varepsilon_0 S}{s\text{-}x} \tag{65}$$

$$C_D=\frac{\varepsilon_0 \varepsilon_r S}{d} \tag{66}$$

Where ε_0 is the vacuum permittivity, ε_r is the relative permittivity of the dielectric material, S is the area of each electrode plate, and x is the plate distance from the leftmost dielectric to electrode II (the plate spacing of C_1), s represents the spacing between the two dielectric layers on the left and right. Since the thickness of the steel sheet is much smaller than s, it can be neglected. $s\text{-}x$ is the plate distance from the dielectric on the right side to electrode II (the plate spacing of C_2), and d is the thickness of the dielectric layer (PTFE film). Detailed parameters are shown in Supplementary Figure 9. Here, electrode II acts as the moving component oscillating between the two fixed electrodes.

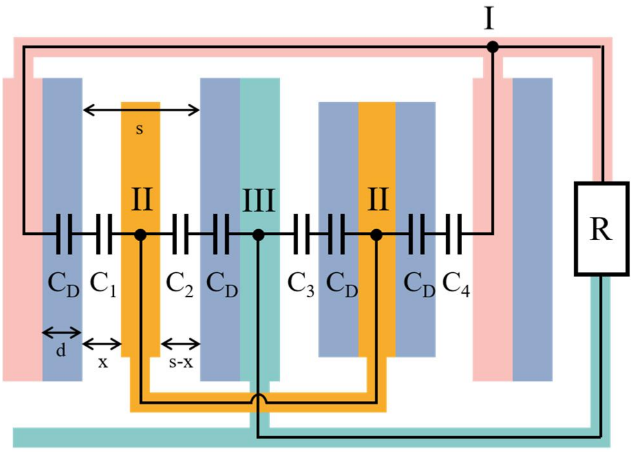

Supplementary Figure 9. Detailed parameters.

According to the KVL for the upper loop I-4-II-3-III, the open-circuit voltage between 1-3 electrode in the RD-TENG model is derived as:

$$V=-\frac{Q_4}{C_4}+\frac{Q_{D4}}{C_D}-\frac{Q_{D3}}{C_D}+\frac{Q_3}{C_3}=\frac{2\sigma_T S}{C_D}\frac{\frac{1}{C_x}-\frac{1}{C_{s\text{-}x}}}{\frac{1}{C_x}+\frac{1}{C_{s\text{-}x}}+\frac{2}{C_D}} \tag{67}$$

Meanwhile, by simplifying equation (63), the short-circuit transferred charge is obtained as:

$$Q=\frac{\frac{\sigma_T S}{C_D}\left(\frac{1}{C_{s\text{-}x}}-\frac{1}{C_x}\right)}{\left(\frac{1}{C_x}+\frac{1}{C_D}\right)\left(\frac{1}{C_{s\text{-}x}}+\frac{1}{C_D}\right)} \tag{68}$$

Where: C_x, $C_{s\text{-}x}$, C_D are show in equations (64)-(66)

To this end, we performed a DESMOS simulation (Supplementary Figure 10), modeling the relationship between the short-circuit transferred charge and the open-circuit voltage with respect to the displacement x of the vibrator between the electrodes, in which: s=5.5×10^{-3} m, S=7×10^{-3} m^2, d=80 μm,

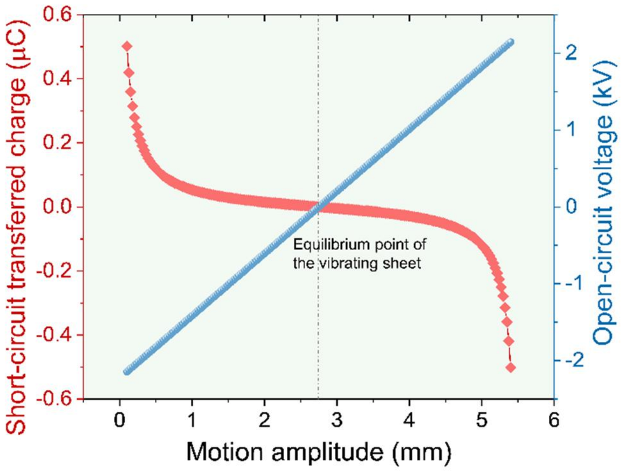

Supplementary Figure 10. RD-TENG Model Visualization: Relationship between the short-circuit transferred charge and open-circuit voltage output with respect to the displacement x of the vibrator between the electrodes.

As shown in the simulation results, the short-circuit transferred charge over one full structural cycle is approximately 1.0 μC, and the open-circuit voltage is around 4.2 kV, which aligns well with the finite element potential simulation results (Supplementary Figure 18). The RD-TENG in this study contains 3.5 structural cycles, so the simulated total short-circuit transferred charge reaches approximately 3.5 μC, while the open-circuit voltage remains at 4.2 kV, due to the parallel configuration of individual cycles. These simulated results closely match our experimental measurements, where the maximum short-circuit transferred charge reached 2.91 μC, and the open-circuit voltage was 2.2 kV. Although the voltage shows a larger deviation, it falls within the reasonable error range expected from non-ideal experimental conditions, consistent with the discrepancies observed in traditional structural models. In particular, the presence of moisture in air leads to an effective dielectric constant higher than that of vacuum, causing the air-related capacitances C_1, C_3, C_2, C_4 throughout the circuit to be greater than theoretical predictions. That is, both C_x and $C_{s\text{-}x}$ tend to be overestimated. According to the open-circuit voltage expression (67), such overestimations in C_x and $C_{s\text{-}x}$ would lead to a lower experimentally measured open-circuit voltage compared to the theoretical model. Therefore, the results of the quantitative analytical model remain consistent with the charge transfer mechanism, finite element simulation, and experimental measurements. Together with the subsequent discussions in the main text, this work establishes a closed-loop validation framework that spans mechanism analysis, charge transfer principles, quantitative modeling, finite element simulations, and experimental verification—forming a mutually corroborating chain of evidence.

Supplementary Note 6. Construction of a comprehensive performance evaluation system for TENG systems

Under the ongoing evolution of TENG architectures and the continuous expansion of their application scenarios, establishing a systematic performance evaluation framework that integrates structural universality with electro-physical insight has become one of the core challenges in the field. To overcome the limitations of previous evaluation methods that focused solely on output voltage, current, or surface charge density, we propose and refine a comprehensive, multidimensional, and engineering-oriented evaluation framework in this study. This framework is designed to enable both horizontal benchmarking across TENG devices and vertical performance optimization from single-unit devices to integrated systems. Specifically, the evaluation framework encompasses three categories of performance metrics:

Supplementary Table 3. Comprehensive evaluation metric. Abbreviations: TSD, triboelectric surface density; SSA, specific surface area; VPD, volumetric power density; VCD, volumetric charge density; SCD, surface charge density.

No.	Attribute	Indicator	Definition	Significance
1	**Structural Performance Metrics**	TSD	Defining the triboelectric surface area per unit device volume	Quantifies the integration level and compactness of energy-harvesting layers within the device. It serves as a measure of both spatial efficiency and material utilization, reflecting the extent to which a structure maximizes its internal active layer density.
		SSA	Defined as the triboelectric surface area per unit device mass	SSA reflects the design's lightweight efficiency and cost-effectiveness. It is particularly important in portable, wearable, or large-area deployable applications where weight and material economy are critical.
2	**Electrical Performance Metrics:**	VCD<br>VPD	Corresponding peak output per unit volume	Reflects the combined effects of internal structural integration and material-coupling efficiency among triboelectric layers, serving as a measure linking electrical and structural performance. reflecting the spatial energy conversion capacity and practical output delivery potential of the device. (Furthermore, the average power on a volumetric basis can be considered)
		SCD<br>SPD	Corresponding peak output per unit area	These are often material-dominated metrics, reflecting the intrinsic charge-generation capability of dielectric materials and the influence of surface engineering (e.g., nano-structuring, charge injection). (Furthermore, the average power on an area basis can be considered)
3	**Application-Oriented Indicators**		These metrics assess the system's readiness for real-world deployment	Manufacturability and packaging compatibility; Energy storage efficiency and charging rates; Load-driving capabilities; Mechanical robustness, durability under cyclic stress; Response uniformity in large-scale arrays; Scenario adaptability across diverse environmental and excitation conditions.

From a long-term and industry-oriented perspective, compact structures, dense integration of energy-harvesting units, and lightweight miniaturization represent key development directions for TENGs in distributed energy and sensing applications. Thus, this study prioritizes the advancement of TSD, positioning it as a central index. Enhancing TSD supports industrial scalability, while also introducing challenges related to electrostatic shielding and charge cancellation—challenges that are systematically addressed in this work through the integration of charge dispatch strategies and structural innovations. TSD serves as a core metric for evaluating spatial utilization and unit-level optimization in dense stacking strategies and complements other energy performance indicators in a mutually reinforcing manner (Supplementary Figure 11). We believe that this multidimensional performance framework will provide a foundational reference for future TENG design, evaluation, and standardization, and will support the field's transition from material–structure–system synergy toward practical integration and industrial deployment.

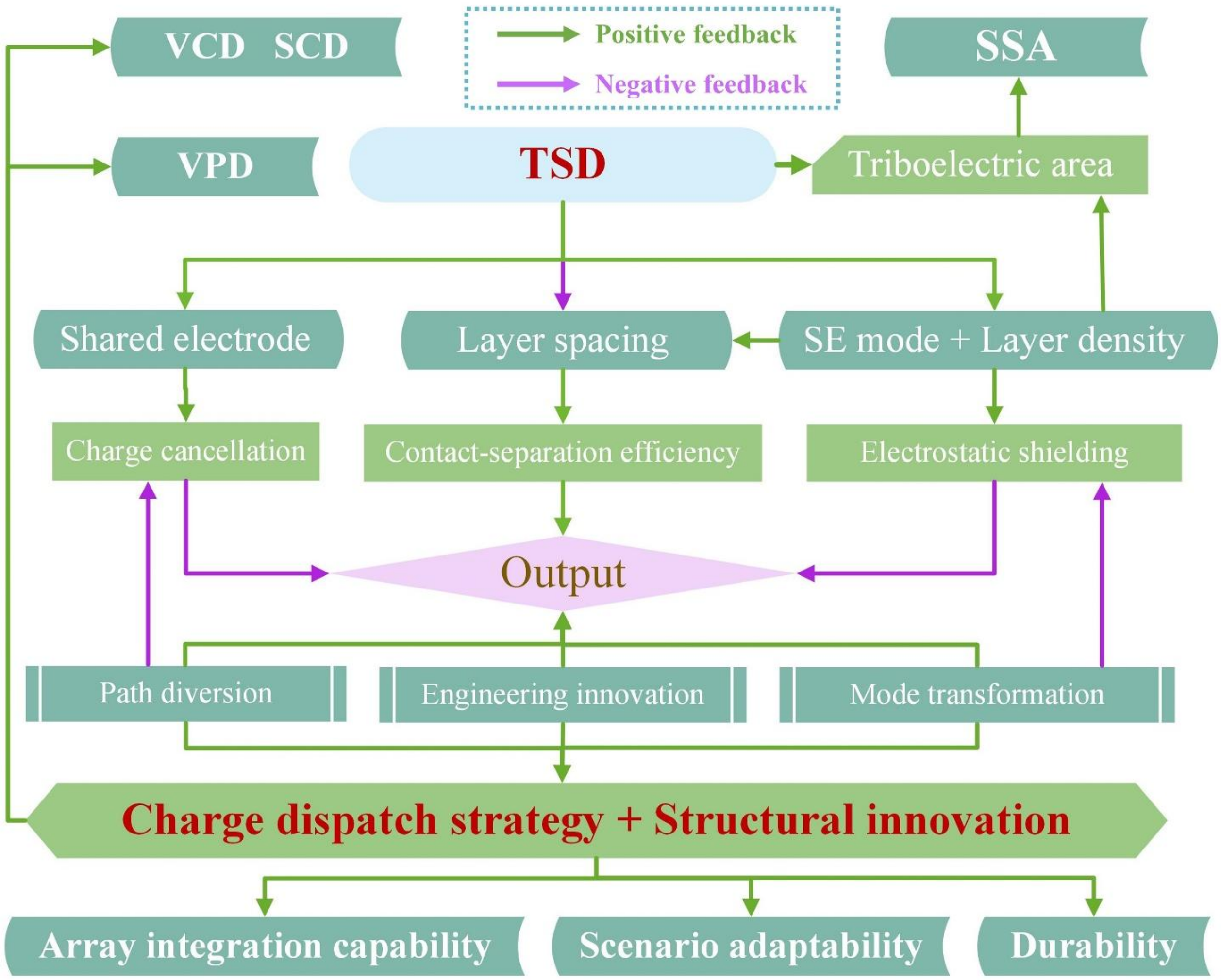

Supplementary Figure 11. Relationship among various evaluation metrics systems. Positive feedback refers to a process where an increase in one quantity promotes the growth of another. In contrast, negative feedback denotes a process where an increase in one quantity suppresses the other.

The evaluation framework of this work is as follows:

1) High-density triboelectric layer and spatial utilization, the record TSD = 2.76 cm^{-1};

2) Lightweight design: 345 g total weight, SSA = 2.90 $cm^2 g^{-1}$;

3) Enhanced output: VPD = 136.74 $W·m^{-3}$; VCD = 11.69 $mC·m^{-3}$; SCD = 42.2 $\mu C·m^{-2}$;

4) System robustness: 12,760 cycles without decay;

5) Energy storage capability: Without employing any power management circuits (e.g., buck-voltage and current-amplifying circuits), a 47 μF capacitor could be charged to 9.7 V in 1 min;

6) Scalable & adaptable: Lego-style modular array integration and real-sea wave harvesting.

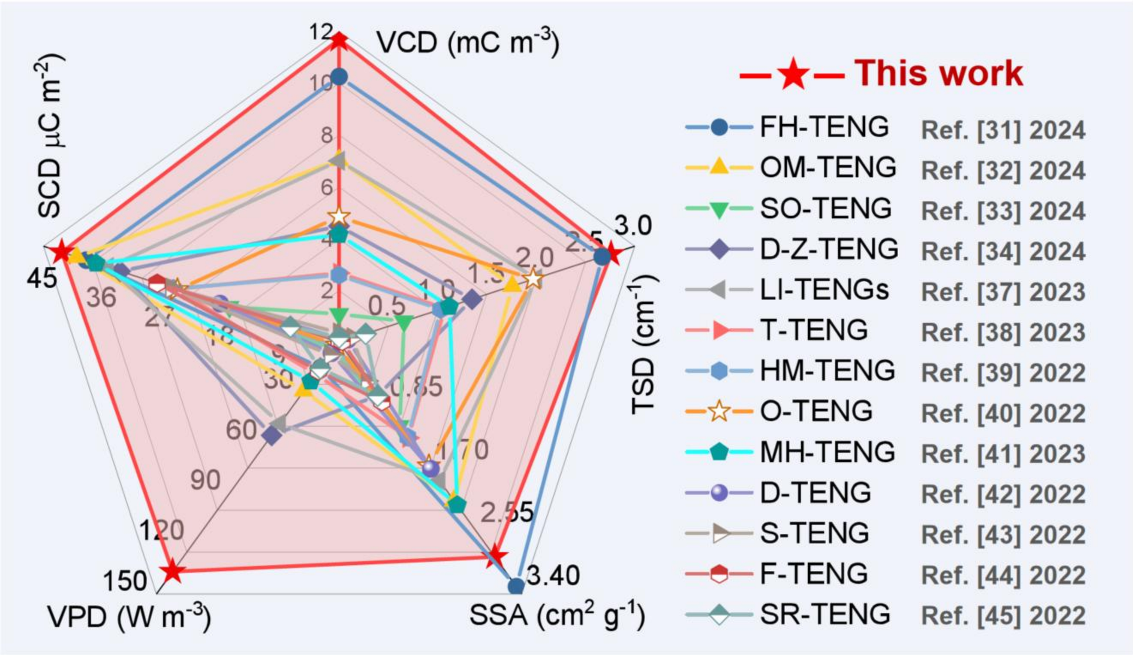

Supplementary Figure 12. Comparison of the structural and electrical performance metrics with other works.

Supplementary Note 7. Orthogonal experimental design.

Introduction

The output of RD-TENG is not only influenced by amplitude and acceleration individually but may also be affected by the interaction between amplitude and acceleration. To scientifically determine the optimal output frequency combination, the best output, and the hierarchical relationships between parameters, we introduced the orthogonal experimental method to systematically explore the optimal combinations of amplitude, acceleration, and terminal velocity. This approach allows us to derive the optimal frequency-to-amplitude ratio. To systematically investigate the response patterns of the device to various input excitation parameters, and to better understand the mechanical-to-electrical energy conversion capability of the RD-TENG, as well as the influencing factors and their hierarchical relationships, we applied the orthogonal experimental method commonly used in engineering applications to study linear and oscillatory excitation input parameters. [48,49]

The orthogonal experimental method is a widely used experimental design approach in engineering applications. Systematically arranging and analyzing multi-factor and multi-level experiments it helps optimize and improve products or processes. The basic principle is to use orthogonal tables to reasonably combine experimental factors and levels, ensuring that both the main effects and interaction effects of each factor are effectively evaluated. The core idea is to leverage the orthogonality of the orthogonal table to balance the factor combinations, thereby reducing the number of experiments while ensuring the representativeness and reliability of the results. Orthogonal tables are characterized by "uniform distribution and neat comparability," which allows for effective separation of factor effects and their interactions, making them suitable for optimizing process parameters, product design, and other fields.

With the assistance of the orthogonal experimental method, the external excitation input parameters that influence the output of the RD-TENG were systematically explored and optimized. This method has significantly advanced subsequent tests and research. Furthermore, the orthogonal experimental method introduced in this study can inspire researchers in the industry by providing methodologies and approaches for finding the optimal combinations in TENG device structure design, energy harvesting system optimization, and power management with multi-component coupling, particularly in research involving multiple variables and parameters.

Experiment 1: Orthogonal experimental design of RD-TENG under direct linear drive.

1. **Clarify the purpose of the experiment.**

Select the optimal combination from the input parameters of the linear motor, including wave

frequency, wave amplitude, acceleration, and terminal velocity. Ultimately, determine the best input and output for the device, as well as the primary and secondary relationships among the input parameters.

Supplementary Table 4. Factor-level table.

Num.	Factors	Levels		Index	Orthogonal table
		Range	Acquisition quantity	2-3 electrode pair	
1	Frequency (Hz)	Obtain the optimal quantity	1	Short-circuit transfer charge: Q	$L_{49}(7^8)$
2	Amplitude (mm)	10-70	7		
3	Acceleration (m s^{-2})	1-13	7		
4	Terminal velocity (m s^{-1})	2-8	7		

2. Determine the factors, levels, and indices, and select the orthogonal table.

Supplementary Table 5. Orthogonal table.

Row Num. / Experiment Num.	1	2	3	4	5	6	7	8
1	1	1	1	1	1	1	1	1
2	1	2	2	2	2	2	2	2
3	1	3	3	3	3	3	3	3
4	1	4	4	4	4	4	4	4
5	1	5	5	5	5	5	5	5
6	1	6	6	6	6	6	6	6
7	1	7	7	7	7	7	7	7
8	2	1	2	3	4	5	6	7
9	2	2	3	4	5	6	7	1
10	2	3	4	5	6	7	1	2
11	2	4	5	6	7	1	2	3
12	2	5	6	7	1	2	3	4
13	2	6	7	1	2	3	4	5
14	2	7	1	2	3	4	5	6
15	3	1	3	5	7	2	4	6
16	3	2	4	6	1	3	5	7
17	3	3	5	7	2	4	6	1
18	3	4	6	1	3	5	7	2
19	3	5	7	2	4	6	1	3
20	3	6	1	3	5	7	2	4
21	3	7	2	4	6	1	3	5
22	4	1	4	7	3	6	2	5
23	4	2	5	1	4	7	3	6
24	4	3	6	2	5	1	4	7
25	4	4	7	3	6	2	5	1
26	4	5	1	4	7	3	6	2
27	4	6	2	5	1	4	7	3
28	4	7	3	6	2	5	1	4
29	5	1	5	2	6	3	7	4
30	5	2	6	3	7	4	1	5
31	5	3	7	4	1	5	2	6
32	5	4	1	5	2	6	3	7
33	5	5	2	6	3	7	4	1
34	5	6	3	7	4	1	5	2
35	5	7	4	1	5	2	6	3
36	6	1	6	4	2	7	5	3
37	6	2	7	5	3	1	6	4
38	6	3	1	6	4	2	7	5
39	6	4	2	7	5	3	1	6

Table Continued.

Row Num. / Experiment Num.	1	2	3	4	5	6	7	8
40	6	5	3	1	6	4	2	7
41	6	6	4	2	7	5	3	1
42	6	7	5	3	1	6	4	2
43	7	1	7	6	5	4	3	2
44	7	2	1	7	6	5	4	3
45	7	3	2	1	7	6	5	4
46	7	4	3	2	1	7	6	5
47	7	5	4	3	2	1	7	6
48	7	6	5	4	3	2	1	7
49	7	7	6	5	4	3	2	1

2. Header design.

Supplementary Table 6. Table header design of experiment 1.

Factors	Acceleration (m s^{-2})<br>A	Amplitude (mm)<br>B	Terminal velocity (m s^{-1})<br>C	/<br>/	/<br>/	/<br>/	/<br>/	/<br>/	Results<br>2-3: Q (nC)
Num.	1	2	3	4	5	6	7	8	/

3. Design experimental scheme.

Supplementary Table 7. Experimental setup and data acquisition (see Supplementary Figure 13).

Row Num. / Experiment Num.	1<br>Acceleration: a (m s^{-2})	2<br>Amplitude: A (mm)	3<br>Terminal velocity: v (m s^{-1})	4<br>/	5<br>/	6<br>/	7<br>/	8<br>/	Results<br>1-2: Q (nC)
1	3	10	2	/	/	/	/	/	74
2	3	20	3	/	/	/	/	/	193
3	3	30	4	/	/	/	/	/	125
4	3	40	5	/	/	/	/	/	103
5	3	50	6	/	/	/	/	/	131
6	3	60	7	/	/	/	/	/	246
7	3	70	8	/	/	/	/	/	114
8	5	10	3	/	/	/	/	/	686
9	5	20	4	/	/	/	/	/	743
10	5	30	5	/	/	/	/	/	456
11	5	40	6	/	/	/	/	/	500
12	5	50	7	/	/	/	/	/	736
13	5	60	8	/	/	/	/	/	738
14	5	70	2	/	/	/	/	/	469
15	7	10	4	/	/	/	/	/	1,290
16	7	20	5	/	/	/	/	/	1,261
17	7	30	6	/	/	/	/	/	1,074
18	7	40	7	/	/	/	/	/	1,084
19	7	50	8	/	/	/	/	/	1,212
20	7	60	2	/	/	/	/	/	1,233
21	7	70	3	/	/	/	/	/	1,122
22	9	10	5	/	/	/	/	/	903
23	9	20	6	/	/	/	/	/	1,137
24	9	30	7	/	/	/	/	/	1,487

Table Continued.

Row Num. / Experiment Num.	1<br>Acceleration: a (m s^{-2})	2<br>Amplitude: A (mm)	3<br>Terminal velocity: v (m s^{-1})	4<br>/	5<br>/	6<br>/	7<br>/	8<br>/	Results<br>1-2: Q (nC)
25	9	40	8	/	/	/	/	/	1,285
26	9	50	2	/	/	/	/	/	1,558
27	9	60	3	/	/	/	/	/	1,354
28	9	70	4	/	/	/	/	/	1,558
29	11	10	6	/	/	/	/	/	933
30	11	20	7	/	/	/	/	/	1,573
31	11	30	8	/	/	/	/	/	1,637
32	11	40	2	/	/	/	/	/	1,716
33	11	50	3	/	/	/	/	/	1,451
34	11	60	4	/	/	/	/	/	1,790
35	11	70	5	/	/	/	/	/	1,845
36	13	10	7	/	/	/	/	/	1,286
37	13	20	8	/	/	/	/	/	1,721
38	13	30	2	/	/	/	/	/	1,953
39	13	40	3	/	/	/	/	/	1,970
40	13	50	4	/	/	/	/	/	1,628
41	13	60	5	/	/	/	/	/	1,802
42	13	70	6	/	/	/	/	/	2,089
43	15	10	8	/	/	/	/	/	1,105
44	15	20	2	/	/	/	/	/	1,664
45	15	30	3	/	/	/	/	/	1,854
46	15	40	4	/	/	/	/	/	1,787
47	15	50	5	/	/	/	/	/	1,907
48	15	60	6	/	/	/	/	/	2,004
49	15	70	7	/	/	/	/	/	2,141

4. The calculation method of the mean analysis in orthogonal experimental design.

In orthogonal experimental design, the range analysis method is mainly used to assess the influence of factors on experimental results by comparing the ranges (the difference between the maximum and minimum values) of the data. The calculation method involves the following steps:

1) Organize experimental data.

Before performing range analysis, the orthogonal experiment should be completed, and the results should be recorded. The original data acquisition is shown in the Figure below.

2) Calculate the average evalue for each factor level.

For each factor at different levels, calculate the average value of all experimental results. Suppose a factor has k levels, and the experimental results at each level are y_{ji} (where j=1, 2, …, k. i = 1, 2, …, m., and m is the number of experiments at each level).

Calculate the average value at each level as follows:

$$\overline{y}_j = \frac{1}{m}\sum_{i=1}^{m} y_{ji} \tag{69}$$

where $\overline{y_j}$ is the average value at the *j-th* level of the factor.

3) Calculate the range for each factor.

For each factor, find the maximum and minimum values at each level and compute the range:

$$R_j = \max(\overline{y}_j) - \min(\overline{y}_j) \tag{70}$$

Where R_j is the range of factor j, representing the fluctuation range of the experimental results for that factor.

4) Compare the ranges of different factors.

Compare the ranges of all factors. A larger range indicates a greater influence on the experimental results, helping to identify significant factors.

5) Determine the optimal factor level combination.

Based on the comparison of ranges, identify the most influential factors and their optimal levels.

6) Summary.

Through the range analysis method in orthogonal experimental design, the influence of multiple factors on experimental results can be quickly evaluated, and the optimal factor-level combination can be selected to optimize the experiment. This method is simple and efficient, and it is suitable for the preliminary screening and optimization of experiments with multiple factors.

Supplementary Table 8. Data analysis.

Row Num. / Experiment Num.	1<br>Acceleration: a (m s^{-2})	2<br>Amplitude: A (mm)	3<br>Terminal velocity: v (m s^{-1})	4<br>/	5<br>/	6<br>/	7<br>/	8<br>/	Results<br>1-3: Q (nC)
y_{j1}	985	6,277	8,667	/	/	/	/	/	/
y_{j2}	4,328	8,292	8,630	/	/	/	/	/	/
y_{j3}	8,276	8,586	8,921	/	/	/	/	/	/
y_{j4}	9,282	8,445	8,277	/	/	/	/	/	/
y_{j5}	10,945	8,623	7,868	/	/	/	/	/	/
y_{j6}	12,449	9,167	8,553	/	/	/	/	/	/
y_{j7}	12,462	9,338	7,812	/	/	/	/	/	/
$\overline{y}_{j1}$	141	897	1,238	/	/	/	/	/	/
$\overline{y}_{j2}$	618	1,185	1,233	/	/	/	/	/	/
$\overline{y}_{j3}$	1,182	1,227	1,274	/	/	/	/	/	/
$\overline{y}_{j4}$	1,326	1,206	1,182	/	/	/	/	/	/
$\overline{y}_{j5}$	1,564	1,232	1,124	/	/	/	/	/	/
$\overline{y}_{j6}$	1,778	1,310	1,222	/	/	/	/	/	/
$\overline{y}_{j7}$	1,780	1,334	1,116	/	/	/	/	/	/
R_j	1,639	437	150	/	/	/	/	/	/
Trend analysis chart	Supplementary Figure 14a.	Supplementary Figure 14b.	Supplementary Figure 14c.	/	/	/	/	/	/
Optimal combination	13	70	4	/	/	/	/	/	/
Primary and secondary factors	Acceleration ＞ Amplitude ＞ Terminal velocity								
Frequency conversion: f	f=2.4, $a=f^2$*0.001*32*A								

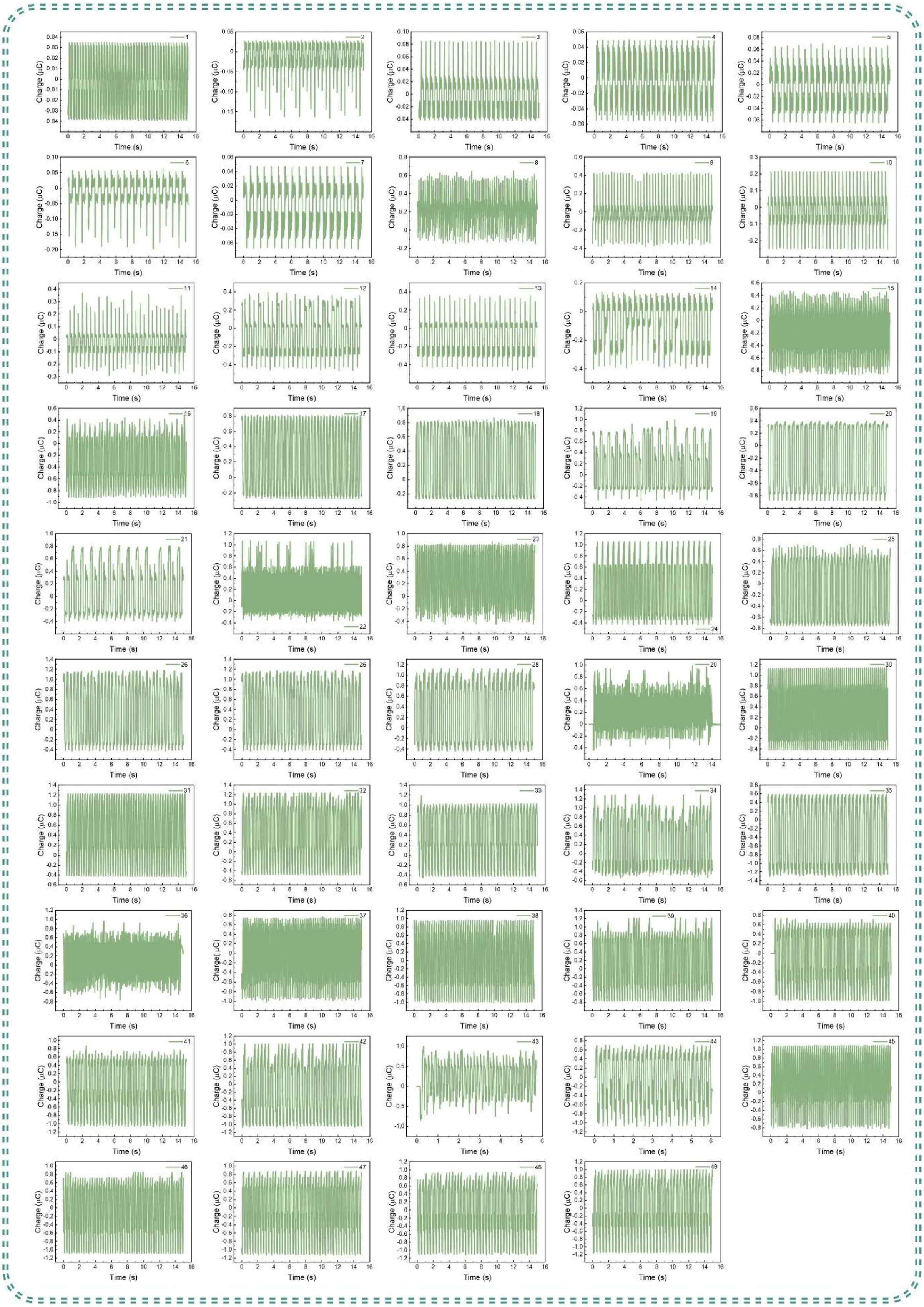

Supplementary Figure 13. The original data acquisition diagram of the orthogonal experimental design of RD-TENG under direct linear drive.

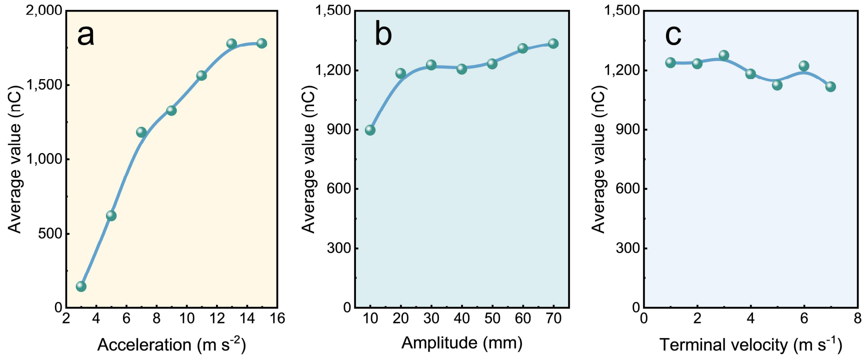

Supplementary Figure 14. The output variation trend of RD-TENG under direct linear drive concerning each input parameter. (a) acceleration, (b) amplitude, (c) terminal velocity

5. Conclusion.

The following conclusions were drawn based on range analysis and subsequent experiments: As the amplitude increases, the output initially increases rapidly, then slows down and tends to saturate. An increase in amplitude means that the vibrating sheet's movement distance becomes greater, allowing more time for the vibrating sheet to complete the contact-separation process with the fixing sheet and efficiently generate electricity. However, as the amplitude continues to increase, the contact-separation efficiency between the vibrating sheet and the fixing sheet gradually reaches an optimal state. When the amplitude reaches approximately 70 mm, the output stabilizes and no longer increases. The relationship between acceleration and output shows a peak: when the acceleration is 13 m s^{-2}, the output reaches its maximum value, after which the output slightly decreases as the acceleration further increases. This is because, at the initial stage of excitation, with constant amplitude, an increase in acceleration results in a rise in frequency. As the frequency increases, the contact-separation speed between the vibrating sheet and the fixing sheet accelerates, and the number of contact-separation cycles per unit time increases. Meanwhile, the enlarged amplitude enhances the contact pressure. The moving part gains higher velocity, thus higher momentum, which leads to more thorough contact with the fixing sheet and an increased output. These two effects work synergistically to enhance the efficiency of triboelectric charge generation and the rate of charge transfer, resulting in a steady improvement in output performance. As the excitation approaches 2.4 Hz / 70 mm, the frequency of external excitation nears the natural frequency of the vibrating sheet system, achieving complete and synchronous contact behavior and optimal coupling efficiency. However, as the frequency continues to increase, the structural response gradually lags behind the excitation rhythm, leading to non-ideal vibrations, slippage, and edge collisions. The moving part cannot separate and return in time during the short duration, resulting in a decrease in output. The overall energy conversion efficiency of the system thus declines. Furthermore, at high frequencies,

multiple vibrating sheets are prone to phase mismatch and asynchronous responses, causing partial electrical output cancellation among different units and further weakening the overall performance. In summary, the output behavior of the RD-TENG is governed by the degree of matching between the excitation and structural dynamics, reflecting the inherently nonlinear coupling characteristics of the triboelectrification-electrostatic induction system. It should also be emphasized that this optimum point is not fixed but can be tuned by adjusting the parameters of the vibrating sheet to modify its natural frequency, thereby adapting to different external vibration environments and ensuring robust engineering scalability. The terminal velocity, as the frequency adjustment variable of the linear motor, has little effect on the output of the device. Ultimately, through comprehensive analysis, the optimal operating conditions of the RD-TENG are found to be at an amplitude of 70 mm, acceleration of 13 m s^{-2}, and terminal velocity of 4 m s^{-1}. The best output frequency, as calculated, is 2.4 Hz.

Experiment 2: Orthogonal experimental design of RD-TENG under linear drive and swinging drive based on frequency-reducing and amplitude-amplifying magnetic repulsion pendulum.

1. **Define the Experimental Objective:** The goal of the experiment is to select two optimal combinations from the six degrees of freedom input parameters: wave frequency, wave amplitude, amplitude coefficient, and swing angle. The first optimal combination is derived for the linear drive, including wave frequency, wave amplitude, and amplitude coefficient, while the second optimal combination corresponds to the swinging drive, which includes wave frequency, swing angle, and amplitude coefficient. Ultimately, the best inputs and outputs for the device in both linear drive and swinging drive states across the six degrees of freedom will be determined, along with the hierarchical relationships between the influence of each input parameter.

Supplementary Table 9. Factor-level table.

Num.	Factors	Levels		Index	Experiment		
		Range	Acquisition quantity	1-3 electrode pair	Num.	Combination	Orthogonal table
1	Frequency (Hz)	0.6-1.8	7	Short-circuit transfer charge: Q	A	1,2,3	$L_{49}(7^8)$
2	Amplitude (mm)	8-20	7				
3	amplitude coefficient	1-7	7		B	1,4,3	$L_{49}(7^8)$
4	Pendulum angle (°)	0-30	7				

2. Determine the factors, levels, and indices, and select the orthogonal table.

The orthogonal table is the same as in Experiment 1.

3. **Header design.**

Supplementary Table 10. Table header design of combination A for the linear drive of experiment 2.

Factors	Frequency (Hz)	Amplitude (mm)	Amplitude coefficient	/	/	/	/	/	Results
	A	B	C	/	/	/	/	/	1-3: Q (nC)
Num.	1	2	3	4	5	6	7	8	/

Supplementary Table 11. Table header design of combination B for swinging drive of experiment 2.

Factors	Frequency (Hz)	Pendulum angle (°)	Amplitude coefficient	/	/	/	/	/	Results
	A	B	C	/	/	/	/	/	1-3: Q (nC)
Num.	1	2	3	4	5	6	7	8	/

4. Design an experimental scheme.

Supplementary Table 12. Experimental setup and data acquisition of combination A for the linear drive.

Row Num. / Experiment Num.	1	2	3	4	5	6	7	8	Results
	Frequency (Hz)	Amplitude (mm)	Amplitude coefficient	/	/	/	/	/	1-3: Q (nC)
1	0.6	8	1	/	/	/	/	/	17
2	0.6	12	2	/	/	/	/	/	31
3	0.6	16	3	/	/	/	/	/	589
4	0.6	20	4	/	/	/	/	/	1,242

Table Continued.

Row Num. / Experiment Num.	1 Frequency (Hz)	2 Amplitude (mm)	3 Amplitude coefficient	4 /	5 /	6 /	7 /	8 /	Results 1-3: Q (nC)
5	0.6	24	5	/	/	/	/	/	1,107
6	0.6	28	6	/	/	/	/	/	1,945
7	0.6	32	7	/	/	/	/	/	2,075
8	0.8	8	2	/	/	/	/	/	144
9	0.8	12	3	/	/	/	/	/	325
10	0.8	16	4	/	/	/	/	/	2,073
11	0.8	20	5	/	/	/	/	/	1,827
12	0.8	24	6	/	/	/	/	/	1,652
13	0.8	28	7	/	/	/	/	/	2,140
14	0.8	32	1	/	/	/	/	/	380
15	1	8	3	/	/	/	/	/	940
16	1	12	4	/	/	/	/	/	2,092
17	1	16	5	/	/	/	/	/	2,061
18	1	20	6	/	/	/	/	/	2,110
19	1	24	7	/	/	/	/	/	2,187
20	1	28	1	/	/	/	/	/	1,314
21	1	32	2	/	/	/	/	/	1,874
22	1.2	8	4	/	/	/	/	/	1,957
23	1.2	12	5	/	/	/	/	/	1,990
24	1.2	16	6	/	/	/	/	/	2,067
25	1.2	20	7	/	/	/	/	/	2,427
26	1.2	24	1	/	/	/	/	/	1,942
27	1.2	28	2	/	/	/	/	/	2,347
28	1.2	32	3	/	/	/	/	/	2,267
29	1.4	8	5	/	/	/	/	/	2,330
30	1.4	12	6	/	/	/	/	/	2,422
31	1.4	16	7	/	/	/	/	/	2,432
32	1.4	20	1	/	/	/	/	/	2,359
33	1.4	24	2	/	/	/	/	/	2,331
34	1.4	28	3	/	/	/	/	/	2,403
35	1.4	32	4	/	/	/	/	/	2,425
36	1.6	8	6	/	/	/	/	/	2,326
37	1.6	12	7	/	/	/	/	/	2,445
38	1.6	16	1	/	/	/	/	/	113
39	1.6	20	2	/	/	/	/	/	2,361
40	1.6	24	3	/	/	/	/	/	2,283
41	1.6	28	4	/	/	/	/	/	2,416
42	1.6	32	5	/	/	/	/	/	2,469
43	1.8	8	7	/	/	/	/	/	2,372
44	1.8	12	1	/	/	/	/	/	111
45	1.8	16	2	/	/	/	/	/	2,412
46	1.8	20	3	/	/	/	/	/	2,432
47	1.8	24	4	/	/	/	/	/	2,379
48	1.8	28	5	/	/	/	/	/	2266
49	1.8	32	6	/	/	/	/	/	2193

5. The Calculation Method of the Range Analysis in Orthogonal Experimental Design (The method is the same as in Experiment 1.)

Supplementary Table 13. Data analysis of combination A for the linear drive.

Row Num. / Experiment Num.	1	2	3	4	5	6	7	8	Results
	Frequency (Hz)	Amplitude (mm)	Amplitude coefficient	/	/	/	/	/	1-3: Q (nC)
y_{j1}	7,006	10,086	6,237	/	/	/	/	/	/
y_{j2}	8,540	9,416	11,500	/	/	/	/	/	/
y_{j3}	12,578	11,747	11,238	/	/	/	/	/	/
y_{j4}	14,998	14,758	14,584	/	/	/	/	/	/
y_{j5}	16,702	13,881	14,050	/	/	/	/	/	/
y_{j6}	14,413	14,831	14,714	/	/	/	/	/	/
y_{j7}	14,165	13,683	16,078	/	/	/	/	/	/
$\overline{y}_{j1}$	1,001	1,441	891	/	/	/	/	/	/
$\overline{y}_{j2}$	1,220	1,345	1,643	/	/	/	/	/	/
$\overline{y}_{j3}$	1,797	1,678	1,605	/	/	/	/	/	/
$\overline{y}_{j4}$	2,143	2,108	2,083	/	/	/	/	/	/
$\overline{y}_{j5}$	2,386	1,983	2,007	/	/	/	/	/	/
$\overline{y}_{j6}$	2,059	2,119	2,102	/	/	/	/	/	/
$\overline{y}_{j7}$	2,024	1,955	2,297	/	/	/	/	/	/
R_j	2,386	2,108	2,083	/	/	/	/	/	/
Trend analysis chart	Supplementary Figure 15a.	Supplementary Figure 15b.	Supplementary Figure 15c.	/	/	/	/	/	/
Optimal combination	1.4	20	4	/	/	/	/	/	/
Primary and secondary factors	Frequency ＞ Amplitude ＞ Amplitude coefficient			/	/	/	/	/	/

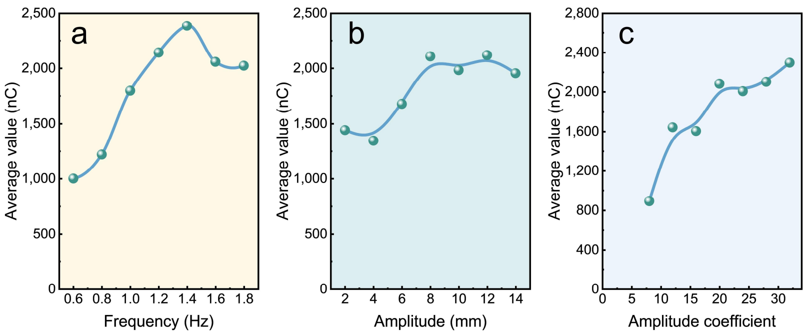

Supplementary Figure 15. The output variation trend of RD-TENG is based on the frequency-reducing and amplitude-amplifying magnetic repulsion pendulum under a six-degree-of-freedom platform linear drive, concerning each input parameter. (a) Frequency, (b) Amplitude, (c) Amplitude coefficient

Supplementary Table 14. Experimental setup and data acquisition of combination B for the swinging drive.

Row Num. / Experiment Num	1	2	3	5	6	7	8	Results
	Frequency (Hz)	Pendulum angle (°)	Amplitude coefficient	/	/	/	/	1-3: Q (nC)
1	0.6	2	1	/	/	/	/	362
2	0.6	4	1.5	/	/	/	/	1,386
3	0.6	6	2	/	/	/	/	1,793
4	0.6	8	2.5	/	/	/	/	1,892

Table Continued.

Experiment Num \ Row Num.	1<br>Frequency (Hz)	2<br>Pendulum angle (°)	3<br>Amplitude coefficient	5<br>/	6<br>/	7<br>/	8<br>/	Results<br>1-3: Q (nC)
5	0.6	10	3	/	/	/	/	2,043
6	0.6	12	3.5	/	/	/	/	2,216
7	0.6	14	4	/	/	/	/	2,160
8	0.8	6	1.5	/	/	/	/	1,922
9	0.8	8	2	/	/	/	/	1,945
10	0.8	10	2.5	/	/	/	/	1,927
11	0.8	12	3	/	/	/	/	1,910
12	0.8	14	3.5	/	/	/	/	1,966
13	0.8	2	4	/	/	/	/	2,003
14	0.8	4	1	/	/	/	/	1,786
15	1	10	2	/	/	/	/	1,862
16	1	12	2.5	/	/	/	/	1,689
17	1	14	3	/	/	/	/	1,928
18	1	2	3.5	/	/	/	/	1,890
19	1	4	4	/	/	/	/	2,020
20	1	6	1	/	/	/	/	1,725
21	1	8	1.5	/	/	/	/	1,998
22	1.2	14	2.5	/	/	/	/	1,961
23	1.2	2	3	/	/	/	/	2,016
24	1.2	4	3.5	/	/	/	/	2,084
25	1.2	6	4	/	/	/	/	1,925
26	1.2	8	1	/	/	/	/	2,038
27	1.2	10	1.5	/	/	/	/	2,097
28	1.2	12	2	/	/	/	/	2,014
29	1.4	4	3	/	/	/	/	2,050
30	1.4	6	3.5	/	/	/	/	2,064
31	1.4	8	4	/	/	/	/	1,947
32	1.4	10	1	/	/	/	/	2,248
33	1.4	12	1.5	/	/	/	/	2,004
34	1.4	14	2	/	/	/	/	1,948
35	1.4	2	2.5	/	/	/	/	2,060
36	1.6	8	3.5	/	/	/	/	1,974
37	1.6	10	4	/	/	/	/	1,979
38	1.6	12	1	/	/	/	/	1,976
39	1.6	14	1.5	/	/	/	/	1,989
40	1.6	2	2	/	/	/	/	1,978
41	1.6	4	2.5	/	/	/	/	2,004
42	1.6	6	3	/	/	/	/	1,997
43	1.8	12	4	/	/	/	/	1,994
44	1.8	14	1	/	/	/	/	1,939
45	1.8	2	1.5	/	/	/	/	1,540
46	1.8	4	2	/	/	/	/	1,951
47	1.8	6	2.5	/	/	/	/	1,942
48	1.8	8	3	/	/	/	/	1,945
49	1.8	10	3.5	/	/	/	/	2,007

6. The Calculation Method of the Range Analysis in Orthogonal Experimental Design (The method is the same as in Experiment 1).

Supplementary Table 15. Data analysis of combination B for the swinging drive.

Row Num. / Experiment Num	1	2	3	5	6	7	8	Results
	Frequency (Hz)	Pendulum angle (°)	Amplitude coefficient	/	/	/	/	1-3: Q (nC)
y_{j1}	11,852	11,849	12,074	/	/	/	/	/
y_{j2}	13,459	13,281	12,936	/	/	/	/	/
y_{j3}	13,112	13,368	13,491	/	/	/	/	/
y_{j4}	14,135	13,798	13,475	/	/	/	/	/
y_{j5}	14,321	14,163	13,889	/	/	/	/	/
y_{j6}	13,897	13,803	14,201	/	/	/	/	/
y_{j7}	13,318	13,891	14,028	/	/	/	/	/
$\overline{y}_{j1}$	1,693	1,693	1,725	/	/	/	/	/
$\overline{y}_{j2}$	1,923	1,897	1,848	/	/	/	/	/
$\overline{y}_{j3}$	1,873	1,910	1,927	/	/	/	/	/
$\overline{y}_{j4}$	2,019	1,971	1,925	/	/	/	/	/
$\overline{y}_{j5}$	2,046	2,023	1,984	/	/	/	/	/
$\overline{y}_{j6}$	1,985	1,972	2,029	/	/	/	/	/
$\overline{y}_{j7}$	1,903	1,984	2,004	/	/	/	/	/
R_j	2,046	242	1,925	/	/	/	/	/
Trend analysis chart	Supplementary Figure 16a.	Supplementary Figure 16b.	Supplementary Figure 16c.	/	/	/	/	/
Optimal combination	1.4	10	4	/	/	/	/	/
Primary and secondary factors	Frequency ＞ Amplitude coefficient ＞ Pendulum angle			/	/	/	/	/

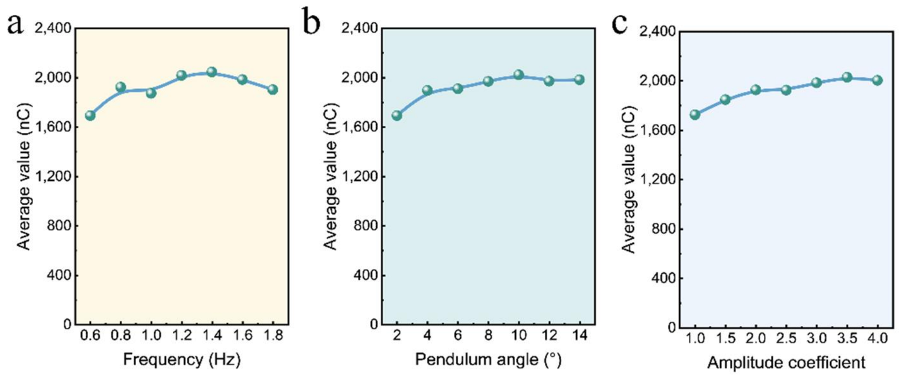

Supplementary Figure 16. The output variation trend of RD-TENG is based on the frequency-reducing and amplitude-amplifying magnetic repulsion pendulum under a six-degree-of-freedom platform swinging drive concerning each input parameter. (a) Frequency, (b) Amplitude coefficient, (c) Pendulum angle

7. Conclusion

Combination A investigates the optimal combination and system's best output of the RD-TENG under linear drive based on the frequency-reducing and amplitude-amplifying magnetic repulsion pendulum, considering the relevant input parameters—frequency, amplitude, and amplitude

coefficient — across the six degrees of freedom. Combination B explores the optimal combination and system's optimal output of the RD-TENG under swinging drive, focusing on the relevant excitation output parameters — frequency, angle, and amplitude coefficient — across the six degrees of freedom. The final result indicates that under linear drive, the system achieves optimal output with frequency, amplitude, and amplitude coefficient set to 1.4 Hz, 20 mm, and 4, respectively, with the hierarchical influence relationship being: frequency > amplitude > amplitude coefficient. For a swinging drive, the optimal combination is: frequency 1.4 Hz, angle 10°, and amplitude coefficient 4, with the impact hierarchy being: angle > frequency > amplitude coefficient. From the trend chart, it can be concluded that under linear driving, all driving parameters collectively influence the results. After eliminating error factors, amplitude, and amplitude coefficient have a relatively smaller impact, while frequency is the primary influencing factor. This indicates that the frequency-reducing and amplitude-amplifying magnetic repulsion pendulum inherently amplifies the small external driving amplitude into a larger working amplitude for the RD-TENG. In the swinging drive mode, individual parameters have little effect on the outcome. As long as the external input meets the system's minimum triggering condition, the RD-TENG system can operate efficiently. This demonstrates the frequency-reducing and amplitude-amplifying magnetic repulsion pendulum's significant potential in reducing response frequency and increasing working amplitude.

Supplementary Note 8. Discussion on materials, engineering choices, scalability, limitations, and future strategies.

1. Selection of core materials and engineering design strategies:

(1) Mn65 silicon–manganese spring steel

In this study, Mn65 silicon-manganese spring steel was selected as the material for the vibrating electrode, fixed electrode, and electrode conductive sheet in the RD-TENG system. This choice represents an optimal engineering solution that balances the requirements of mechanical performance, electrical function, structural integration, and manufacturability across multiple dimensions. Its advantages are summarized as follows:

1) Outstanding elastic modulus and structural rigidity: Mn65 exhibits a high elastic modulus (~200 GPa) and tensile strength ($\sigma_t \geq 980$ MPa), enabling it to deliver stable and repeatable vibration responses under high-frequency contact-separation excitation. Moreover, it serves as a structural backbone in the high-density triboelectric layer stacking, effectively resisting deformation and mitigating performance degradation caused by stress concentration.

2) Good electrical conductivity and positive triboelectric polarity: As a metallic conductor with a triboelectrically positive surface, Mn65 forms an ideal triboelectric pair with materials such as PTFE. This allows the vibrating sheet to simultaneously function as the triboelectric layer, electrode layer, and self-supporting layer, enabling wire-free integrated structural design.

3) Excellent fatigue resistance and surface stability: Its high surface hardness and abrasion resistance ensure stable frictional performance during prolonged contact–separation cycles and suppress mechanical fatigue. In addition, its smooth and flat surface improves the conformity and completeness of each contact event, thereby enhancing energy conversion efficiency and prolonging device lifespan.

4) Intrinsic mass-loading characteristic: With a moderate density (7.85 g cm^{-3}), the material inherently provides inertial mass to the vibrating sheet. When combined with the elastic bridge, it functions as a simple harmonic oscillator, expanding the frequency response of the mech-elect conversion.

5) Superior machinability and structural design adaptability: Mn65 spring steel offers excellent processability and can be patterned using laser etching technology to fabricate the integrated vibrating electrode and fixed electrode with high precision, thereby enhancing manufacturing accuracy and interlayer consistency.

The relevant material parameters are summarized in the table below.

Supplementary Table 16. Material parameters of Mn65 spring steel (based on Chinese National Standard GB/T 1222-2016, ASTM A689, and the Mechanical Engineering Materials Handbook)

Category	Property	Value / Range	Unit	Notes
**Chemical Composition**	Carbon (C)	0.62–0.70	wt %	Primary strengthening element
	Manganese (Mn)	0.90–1.20	wt%	Improves hardenability
	Silicon (Si)	0.17–0.37	wt%	Solid-solution strengthening
	Phosphorus (P)	≤0.035	wt%	Controlled impurity
	Sulfur (S)	≤0.035	wt%	Controlled impurity
**Mechanical Properties**	Ultimate tensile strength (σ_t)	≥980 (up to 1200–1600 after cold rolling)	MPa	Dependent on heat treatment
	Yield strength (σ_Y)	≥785	MPa	—
	Young's modulus (E)	~200	GPa	Typical for steels
	Elongation at break (δ)	≥8 (annealed state)	%	—
	Hardness (annealed)	≤ HB 285 (up to HRC 40–50 when cold-rolled)	—	—
**Heat Treatment Parameters**	Quenching temperature	830–860	°C	Oil quenching
	Tempering temperature	400–500	°C	Balances strength and toughness
	Annealing temperature	680–720	°C	Softened by controlled cooling
**Spring Performance**	Fatigue limit (10^7 cycles)	450–600	MPa	Can be improved by 20%–30% via shot peening
	Service temperature range	−40 to +200	°C	Derating is required at elevated temperatures
**Physical Properties**	Density	7.85	g cm^{-3}	—
	Coefficient of thermal expansion	11.5×10^{-6}	/°C	In the range of 20–100 °C
	Thermal conductivity	~50	W/ (m K)	—

(2) Magnet

In the scenario adaptability expansion strategy, magnets are employed as the restoring force source for swinging, functioning analogously to mechanical springs by providing repeatable restoring forces under external excitation. However, compared to traditional springs, magnetic repulsion structures not only fulfill the mechanical restoring role but also offer superior performance in structural stability, response sensitivity, mechanical lifespan, protective adaptability, nonlinear tunability, and scalability—aligning with our goal of achieving high sensitivity and adaptability in low-frequency, low-amplitude natural energy environments.

1) **High-sensitivity response:** Traditional springs rely on elastic deformation and follow Hooke's Law, where the restoring force is proportional to displacement:

$$F_{spring}(r)=-kx \tag{71}$$

where k is the spring stiffness coefficient and x is the displacement, resulting in a linear restoring force. In contrast, the repulsive force between permanent magnets exhibits a distinctly nonlinear

distance-dependent response. According to the magnetic dipole model, the repulsive force F between magnets varies with distance x approximately as:

$$F_{mag}(r) \propto \frac{A}{x^n} \tag{72}$$

A is the magnetic repulsion constant, influenced by the structural and material properties of the magnets. The exponent n is greater than 2, typically ranging between 2 and 4, depending on the magnet shape and boundary conditions. As shown in equation (72), the magnetic repulsive force increases rapidly as the distance between the magnets decreases, enabling a stronger restoring force even under small displacement perturbations. This results in a higher effective system stiffness during the initial response phase, which helps overcome startup inertia and facilitates rapid activation at low frequencies.

To visually compare the magnitude and trend of restoring forces with a mechanical spring, we set A = 0.5 N mm^2, k = 1 N mm^{-1}, and n = 2, and plotted the force-displacement relationship in Supplementary Figure 17. In the low-displacement region (0-2.5 mm), the magnetic repulsion curve exhibits a steeper slope than the linear spring, indicating greater responsiveness and sensitivity to small excitations.

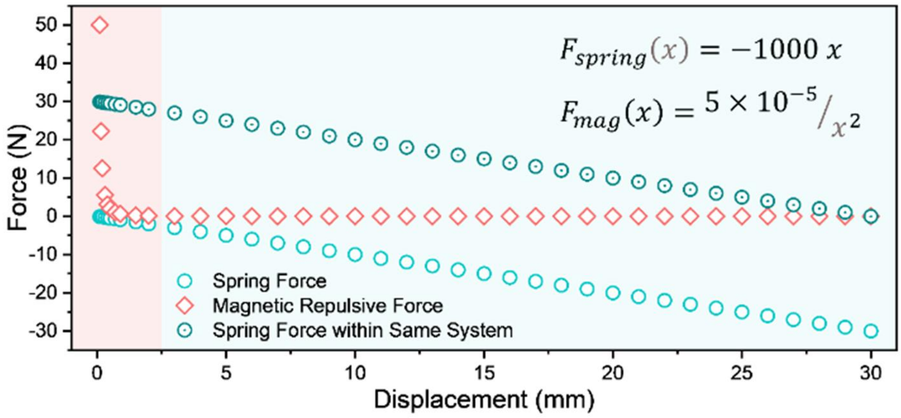

Supplementary Figure 17. Comparison of restoring forces for magnetic repulsion and traditional spring.

2) High dynamic tunability and environmental adaptability. The restoring stiffness k of traditional springs is typically fixed by their material properties and geometry, requiring physical replacement to alter performance. In contrast, the equivalent restoring stiffness k_{eff} of a magnetic repulsion system can be continuously tuned by adjusting the spacing, orientation, and size of the magnets. This is reflected in the repulsion constant A in equation (72) and Supplementary Table 17. The simplified model is:

$$A \approx \frac{\mu_0}{4\pi} \frac{6\, m_1\, m_2}{1} \tag{73}$$

Supplementary Table 17. Factors affecting the magnetic repulsion constant (A).

Parameter	Meaning
μ_0	Vacuum permeability, $\mu_0 = 4\pi \times 10^{-7}$ H m^{-1}
m_1, m_2	Effective magnetic moments (or approximated magnetic charges) of the two magnets
**Geometric parameters**	Magnet shape (e.g., cylinder, sphere), area, and alignment direction
**Material properties**	Type of permanent magnet (e.g., NdFeB, SmCo), magnetic energy product, etc.

Moreover, according to the natural frequency equation of a pendulum system in vibration theory:

$$f_0=\frac{1}{2\pi}\sqrt{\frac{k_{eff}}{I}} \tag{74}$$

where k_{eff} represents the system's effective stiffness, and I denotes the moment of inertia. Since the k_{eff} of a magnetic repulsion system is far more adjustable than that of a rigid mechanical spring, it is easier to match with low-frequency excitation sources through careful magnetic spacing design. This significantly improves both the energy coupling efficiency and the oscillation amplitude. In contrast, conventional springs often suffer from insufficient preload, limited displacement amplitude, and response lag—especially under unstable and asymmetric excitations like ocean waves, resulting in lower matching efficiency and energy conversion rates. Therefore, in our system design, the magnetic structure parameters can be flexibly adjusted according to different excitation scenarios and TENG configurations to achieve frequency matching and optimized startup response, enhancing adaptability to varying wave conditions.

3) Mechanical advantages. From an engineering standpoint, the magnetic repulsion system offers superior integration compatibility with our proposed floating oscillation platform. Unlike conventional springs, which require auxiliary components such as central bearings, guide rails, and limiters, the magnetic repulsion structure forms a three-dimensional repulsive field simply through spatial magnet placement, enabling a more compact and streamlined design. Beyond structural simplicity, magnetic systems also outperform mechanical springs in corrosion resistance-critical for harsh marine environments. Traditional springs, often made of carbon steel or silicon-manganese alloys, are prone to oxidation, rust, and fatigue failure under prolonged exposure to seawater. Even stainless steel variants can degrade over time due to stress concentration and chloride ion corrosion. Moreover, fully sealing spring-based assemblies is challenging, as springs typically link two moving parts. In contrast, the N52 NdFeB magnets used in this work are coated with high-performance anti-corrosion layers, nickel-copper-nickel plating, and epoxy resin, which effectively isolate the magnetic core from air and moisture. Additionally, all magnets are sealed within PLA housing and

waterproof adhesives, minimizing exposure to humidity. As non-contact force sources, these magnets do not undergo mechanical wear or metal-on-metal contact, thus avoiding frictional corrosion and fatigue damage. Therefore, in marine applications, magnetic repulsion structures offer enhanced durability, lower maintenance demands, and improved long-term mechanical reliability.

2. Strategy scalability.

For a technology to evolve from experimental demonstration to industrial-scale and commercial deployment, it must possess both a sustainable performance output mechanism and the ability to be manufactured and scaled. This is particularly critical for triboelectric nanogenerators (TENGs), whose performance is highly dependent on structural configurations and interfacial conditions. Therefore, scalability, universality, and system compatibility are not only reflections of scientific merit but also key determinants of practical applicability. Parameters such as assembly simplicity, modular compatibility, and suitability for batch manufacturing will dictate whether the technology can be widely adopted in future deployment scenarios.

At the device level:

1) Charge dispatch strategy: The proposed three-electrode charge dispatch strategy, which integrates alternate film coating and a short-circuited central electrode, exhibits structural compatibility and mode transferability across diverse TENG architectures. Its core lies in forming dual charge migration loops—an internal loop via a freely moving, short-circuited electrode between two fixed electrodes, and an external loop via the terminal outputs—which together optimize the electric field distribution and charge transfer pathways. This helps alleviate common issues in high-density stacked TENGs, such as electrostatic shielding and charge cancellation. In free-standing contact-separation (FCS) mode devices, this strategy proves particularly advantageous. Traditional multilayer FCS-TENGs often rely on insulating spacers to avoid interlayer interference and short-circuit risks, which compromises integration density and charge utilization. Here, by introducing a short-circuited vibrating electrode into a shared electrode density FCS configuration, the device achieves high integration while maintaining efficient output, thus breaking the trade-off between stacking density and performance. Moreover, this mechanism is adaptable to simplified single-layer FCS systems by incorporating a freely movable electrode that builds a symmetric charge dispatch loop with the fixed layer or ground, offering a modular and scalable design approach.

Importantly, this strategy is also extendable to the free-standing sliding mode (FS-TENG) and more complex hybrid structures. In FS-mode devices, sliding components are typically mechanical-only. By modifying the slider to include oppositely polarized dielectric films and connecting it as a

third electrode, it can simultaneously serve triboelectric, charge redistribution, and output functions, thereby enhancing performance through path-controlled charge dispatch. Broadly speaking, any TENG structure with multiple electrodes and relative-motion interfaces, e.g., contact-separation (CS), rolling slide (RS), and solid-liquid contact modes (LS), can benefit from this strategy, as long as at least four definable electrodes (triboelectric layer) and controlled polarity arrangements are present. Through functional reassignment and reconnection of internal and external electrodes, charge dispatch paths can be reconstructed, boosting both output performance and structural integration.

2) Vibrating sheets: The vibrating sheet's resonant frequency can be precisely tuned by adjusting parameters such as the number, width, and length of connecting bridges, sheet thickness, and outer diameter. More and wider or shorter bridges, thicker sheets, and smaller diameters all increase the resonant frequency, making the device more suitable for harvesting energy from high- or ultra-high-frequency sources, such as wind-induced vibration, tip oscillation, or aeroelastic flutter.

3) Contact push pin electrode connection: Wire-free architecture is essential for multilayer TENGs. As the number of layers increases, internal wiring becomes increasingly complex, occupying space, hindering contact-separation efficiency, and introducing risks of short circuits or disconnection. The push pin method eliminates the need for wires, adhesives, or soldering by providing direct, modular electrical contact between electrode groups. This approach improves spatial efficiency, enhances mechanical contact, simplifies assembly, and is scalable—especially for compact or rapidly prototyped multilayer devices.

4) Looping stacking method for power units: This method enables rapid assembly of triboelectric layers by stacking onto latches in a ring-like fashion. The interlayer separation can be adjusted using ring gaskets of different thicknesses, avoiding gluing, taping, or welding steps. It significantly simplifies fabrication and provides a flexible, scalable strategy for assembling high-density, multilayer TENG devices. Gasket size and thickness can be tailored to fit specific structural or environmental demands, and the stacking method is transferable to other TENG designs.

External scalability aspects:

1) Rotating-clasp-device array assembly: Device arrays are essential for scaling TENGs toward commercial and industrial applications. The proposed clasp-based modular assembly—comprising “push pin–insulating sheet–electrode conductive sheet–conductive sponge–push pin” modules—enables rapid, stable, and solder-free parallel integration of multiple devices. The process resembles battery series/parallel connection or LEGO-style stacking, offering a universal reference for assembling compact TENG arrays.

2) Frequency-reducing and amplitude-amplifying magnetic repulsion platform: The magnetic platform's stiffness and repulsive constant can be tuned by modifying the number and placement of magnets, their strength, and the length or spacing of the pendulum rod and deflectors. This adjustability enhances responsiveness to varying excitations. Additionally, the upper end of the pendulum rod can accommodate different TENG architectures, allowing the platform to adapt to both varying stimulus intensities and diverse structural modes.

3. Potential limitations and future mitigation strategies.

Although the RD-TENG with a three-electrode high-density triboelectric layer exhibits clear advantages in mitigating electrostatic shielding and charge cancellation, improving structural integration, optimizing engineering design, and enhancing energy output performance—along with demonstrated stability and adaptability under real oceanic conditions—there remain several limitations to be further addressed:

First, while the short-circuited electrode charge dispatch strategy has significantly improved output performance in high-density stacked architectures and has shown theoretical scalability to other modes, its experimental validation remains largely limited to the FCS mode. Although feasibility analyses have been conducted for application in FS, CS, RS, and SL modes, practical implementation and validation are still ongoing. In future work, we aim to extend this charge dispatch mechanism across a broader range of TENG architectures, integrating reconfigurable electrode structures and alternating polarization strategies to enhance cross-mode compatibility and functionality.

Second, corrosion remains a critical challenge in marine deployments. While this work has implemented multiple layers of engineering protection—structurally, the RD-TENG operates as a floating platform and avoids direct seawater contact, with the core generator module staying above the waterline during operation; in terms of material sealing, a tightly enclosed PLA housing with acrylic sealing plates and industrial-grade adhesives (e.g., Dow Corning 732) is employed to block all joints, and the outer stacked layer further protects the inner stacked layer from external vapor ingress. Field tests under moderate oceanic disturbances (tidal height of 4.5 m, wind speed of 29 km h^{-1}) confirmed sealing integrity and stable output, with no signs of water ingress, corrosion, or degradation. However, long-term stability under extreme conditions—such as prolonged salt spray, UV exposure, and turbulence—requires further empirical validation. Stainless steel was not selected due to its limited elasticity, poor conductivity, and difficulty in laser processing. In contrast, Mn65 spring steel offers superior elasticity, conductivity, and processability, making it more suitable for

the integrated device design. Nonetheless, we acknowledge that in prolonged or extreme marine environments, Mn65 may still face corrosion risks. Future work will explore surface treatments (e.g., electroplating, epoxy coatings), enhanced module sealing, the use of elastic composites or corrosion-resistant alloys, and systematic evaluation through corrosion testing and simulation.

Moreover, the marine environment is inherently unpredictable, especially under extreme conditions such as storm surges, high-frequency turbulence, or the superposition of intense waves. In such scenarios, the MRP platform may experience severe swaying, overturning risks, or amplified structural shocks. In future work, we plan to enhance the anti-overturning capability and output stability of the RD-TENG under harsh sea states by integrating six-degree-of-freedom anti-tilt structures, flexible limiting frames, high-strength support materials, and intelligent buoy stabilization technologies. Simultaneously, we also intend to introduce numerical simulations and wave tank experiments to systematically investigate the dynamic response behaviors of the system under various sea conditions.

Finally, although the rotating-clasp-device and contact push-pin connection strategies enhance the integration stability and electrical connectivity of RD-TENG arrays, challenges remain under high-frequency excitation. In practice, asynchronous response and incomplete output superposition have been observed across modules due to platform-induced oscillations during inertial halts. These effects introduce phase mismatches among vibrating sheets, compromising synchronized contact-separation behavior and reducing energy summation efficiency. Moving forward, we plan to introduce flexible damping layers, limiters, and local frequency tuning designs to improve mechanical coordination among modules. Additionally, we will validate the array under periodic sinusoidal excitation platforms that better simulate real-world vibration scenarios, aiming to enhance synchronization and energy integration efficiency in practical conditions.

Supplementary Figures

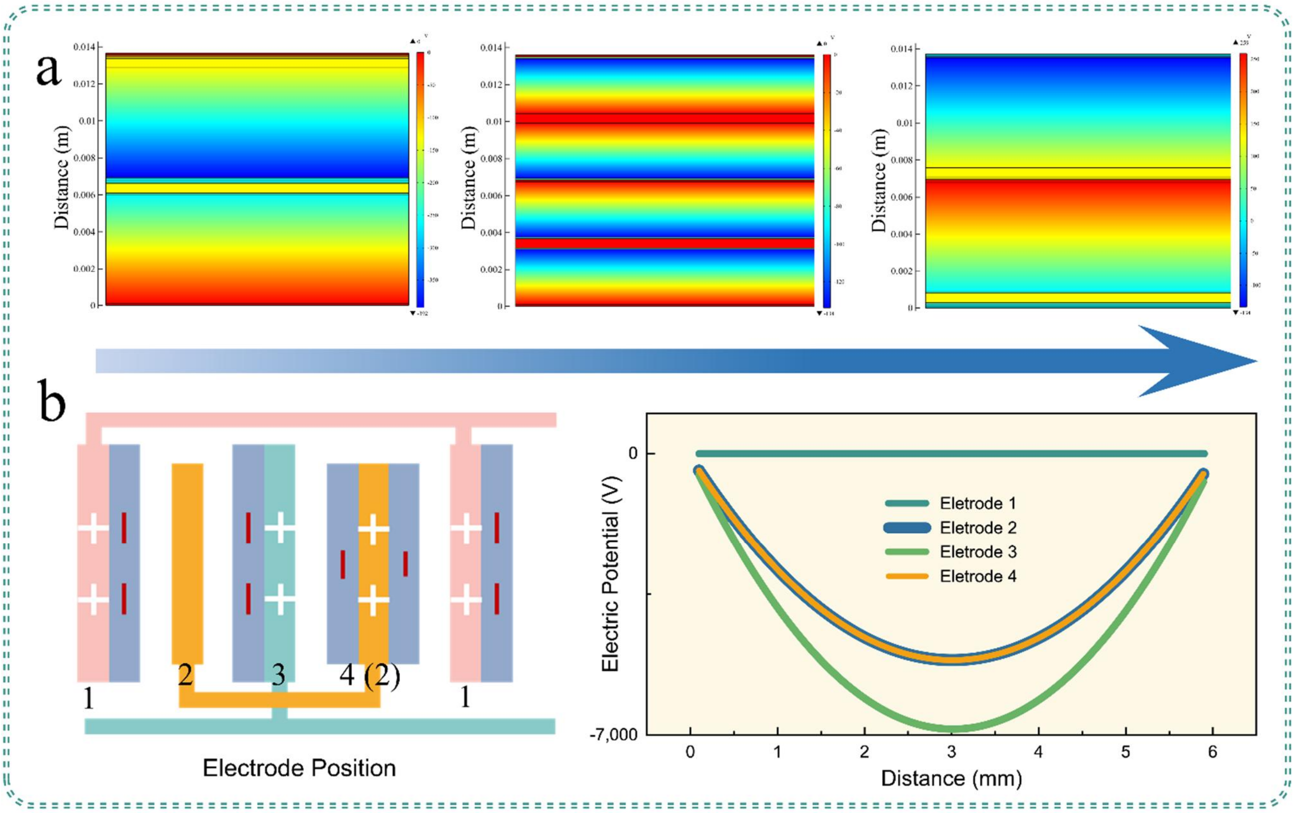

Supplementary Figure 18. (a) Simulation of the electric potential of the vibrating sheet moving from one end to the other in the RD-TENG (the electrode is grounded). (b) Positions of each electrode and their electric potential variation curves as the vibrating sheet oscillates from one end to the other.

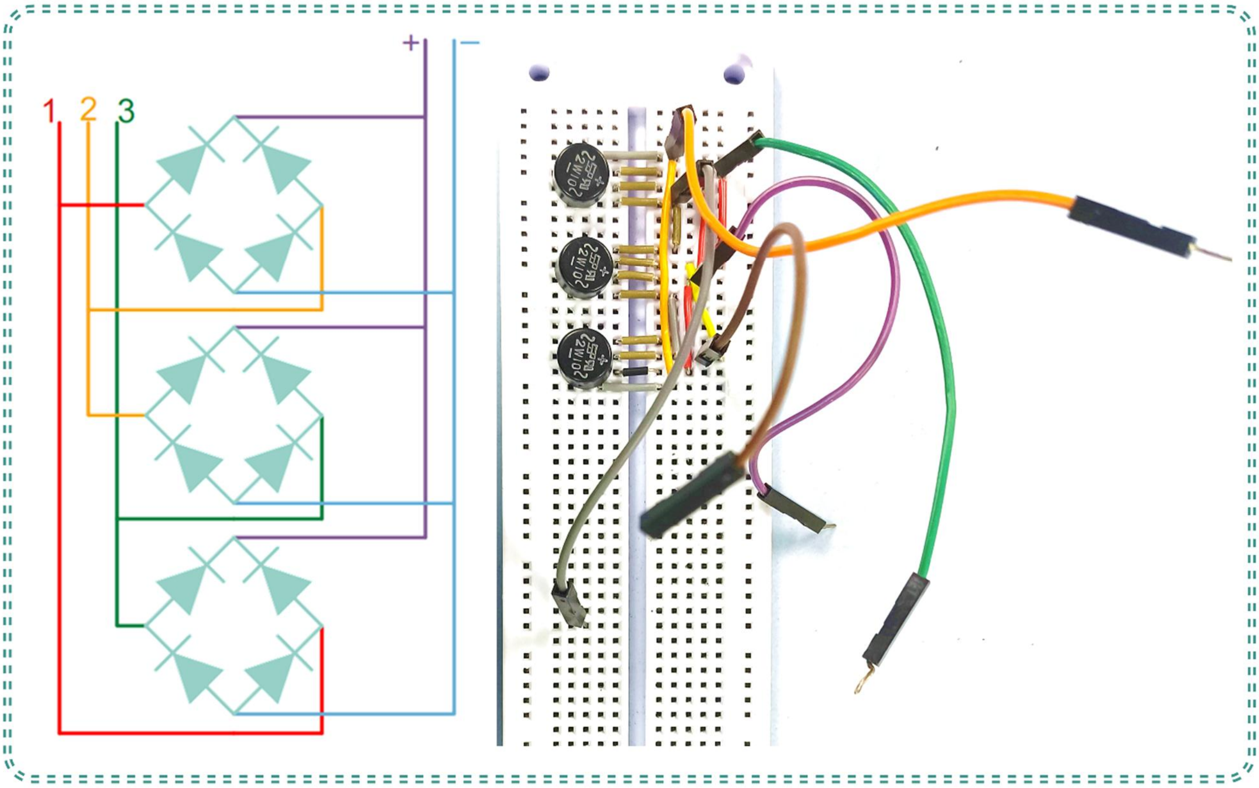

Supplementary Figure 19. Three-electrode rectifier circuit diagram and its physical circuit image.

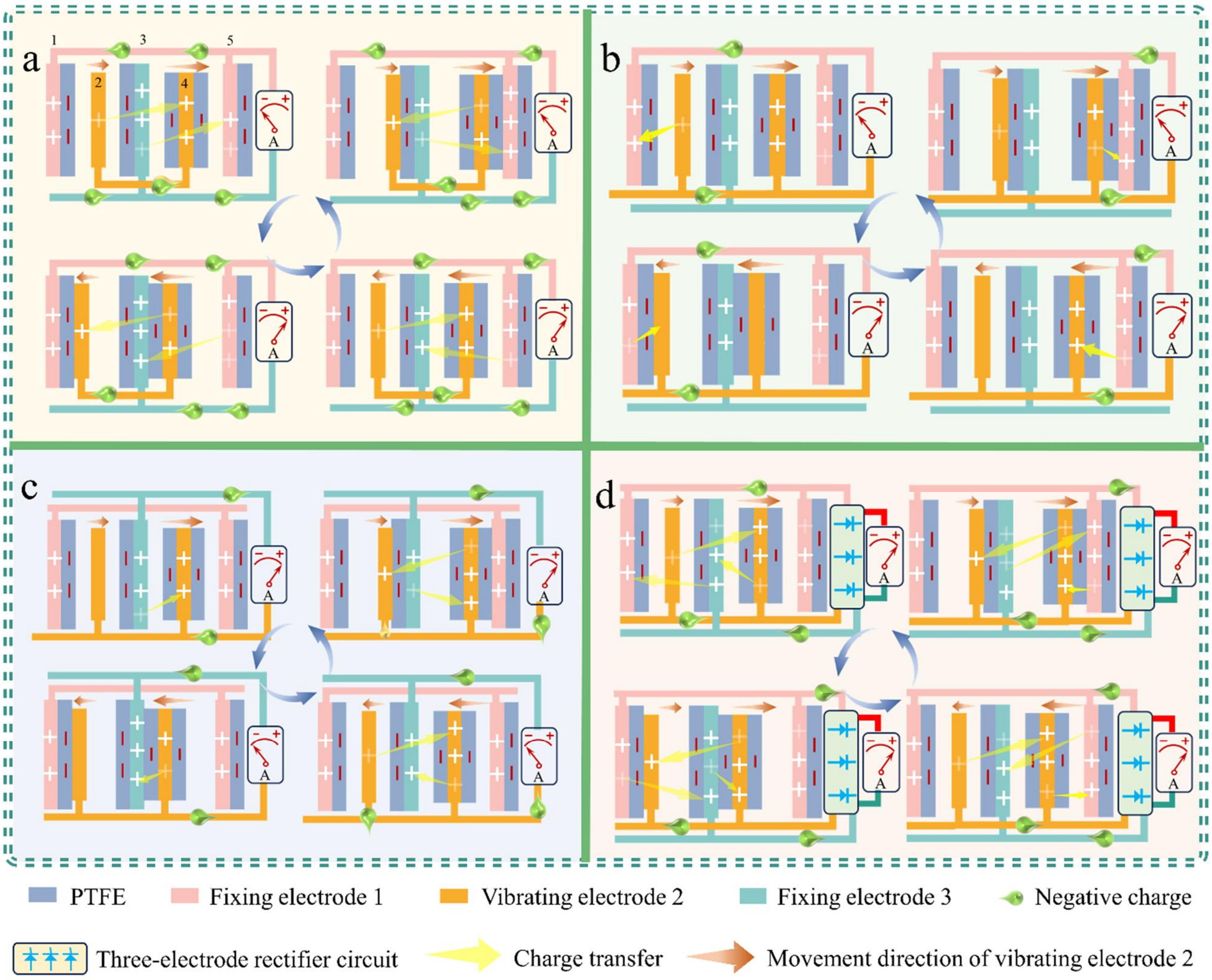

Supplementary Figure 20. Charge dispatch and transfer schematic diagram for each electrode pair of RD-TENG (the electrode is grounded). (a) 1-3 electrode pair. (b) 1-2 electrode pair. (c) 2-3 electrode pair. (d) rectified electrode pair.

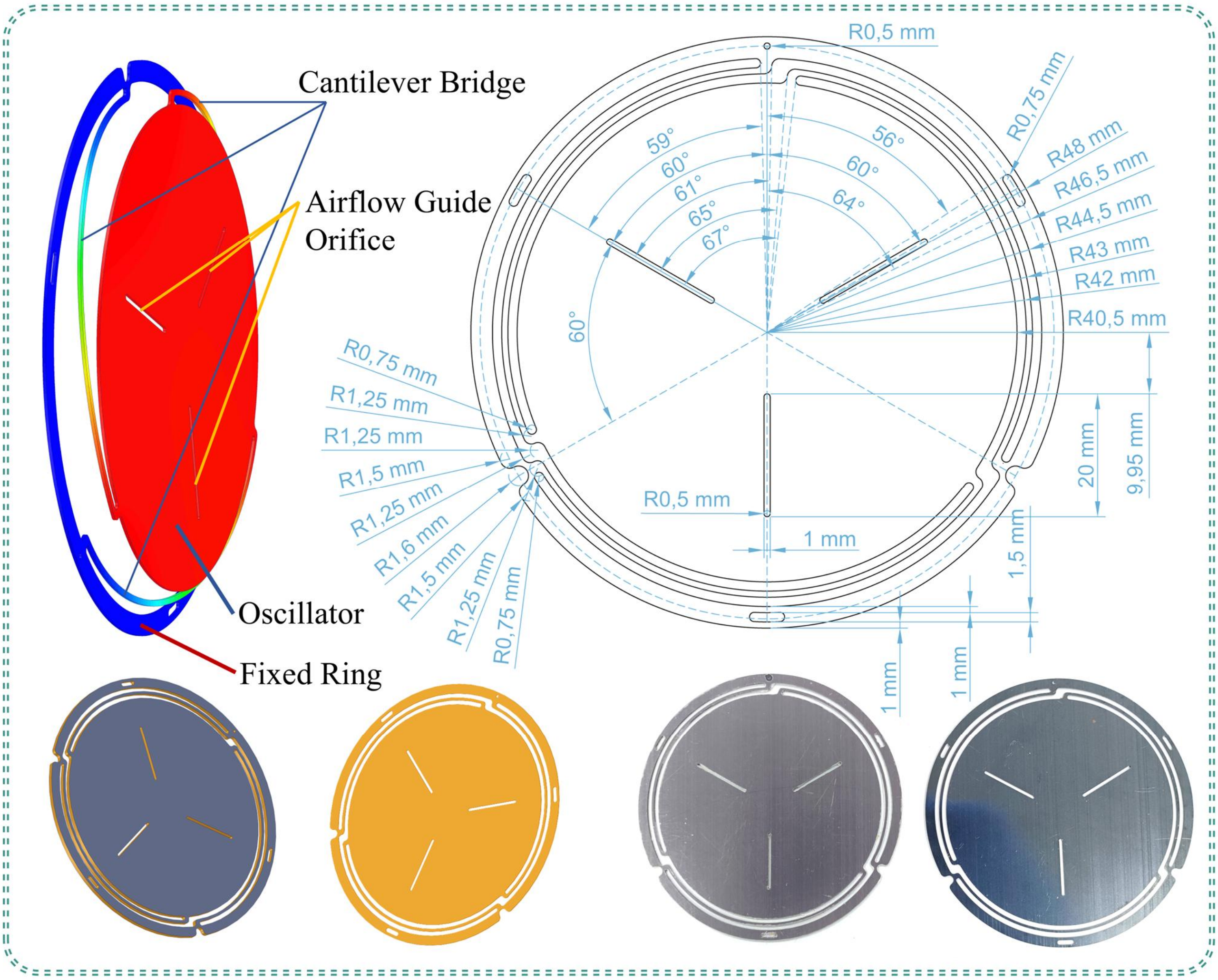

Supplementary Figure 21. Vibrating sheet style, parameters, model, and physical image.

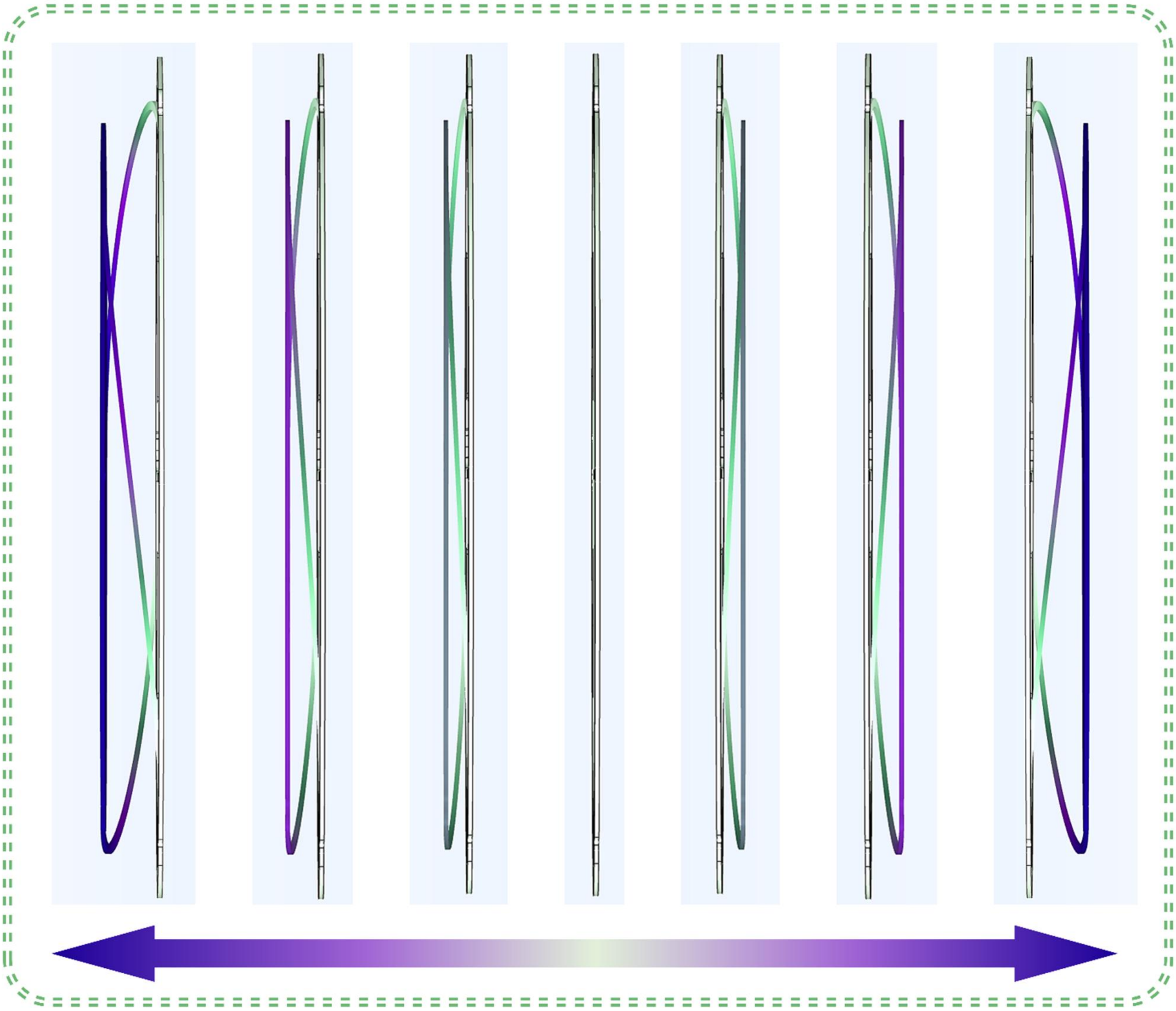

Supplementary Figure 22. Vibration simulation of the vibrating steel sheet.

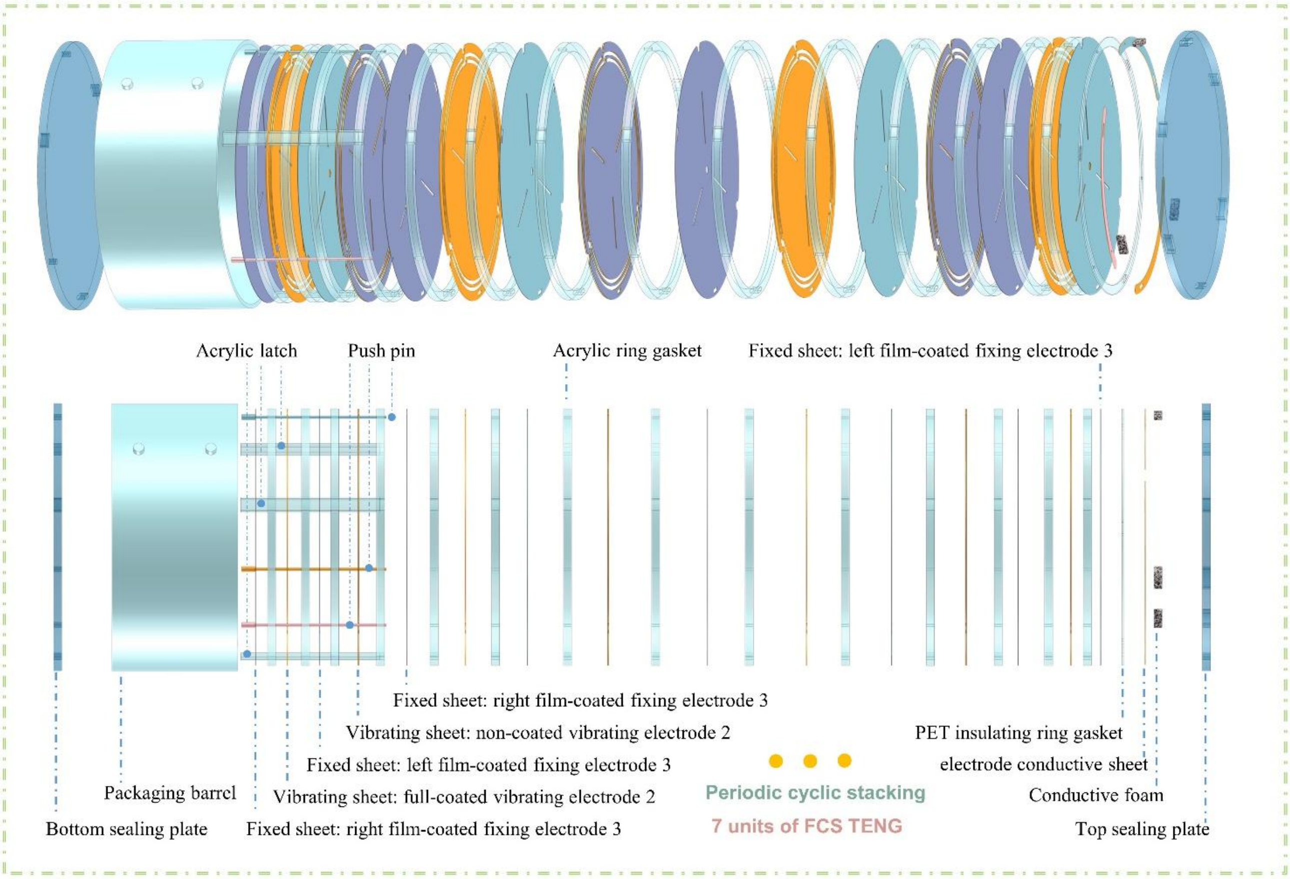

Supplementary Figure 23. Exploded isometric view and front view of the full-scale structural model of the RD-TENG with all components.

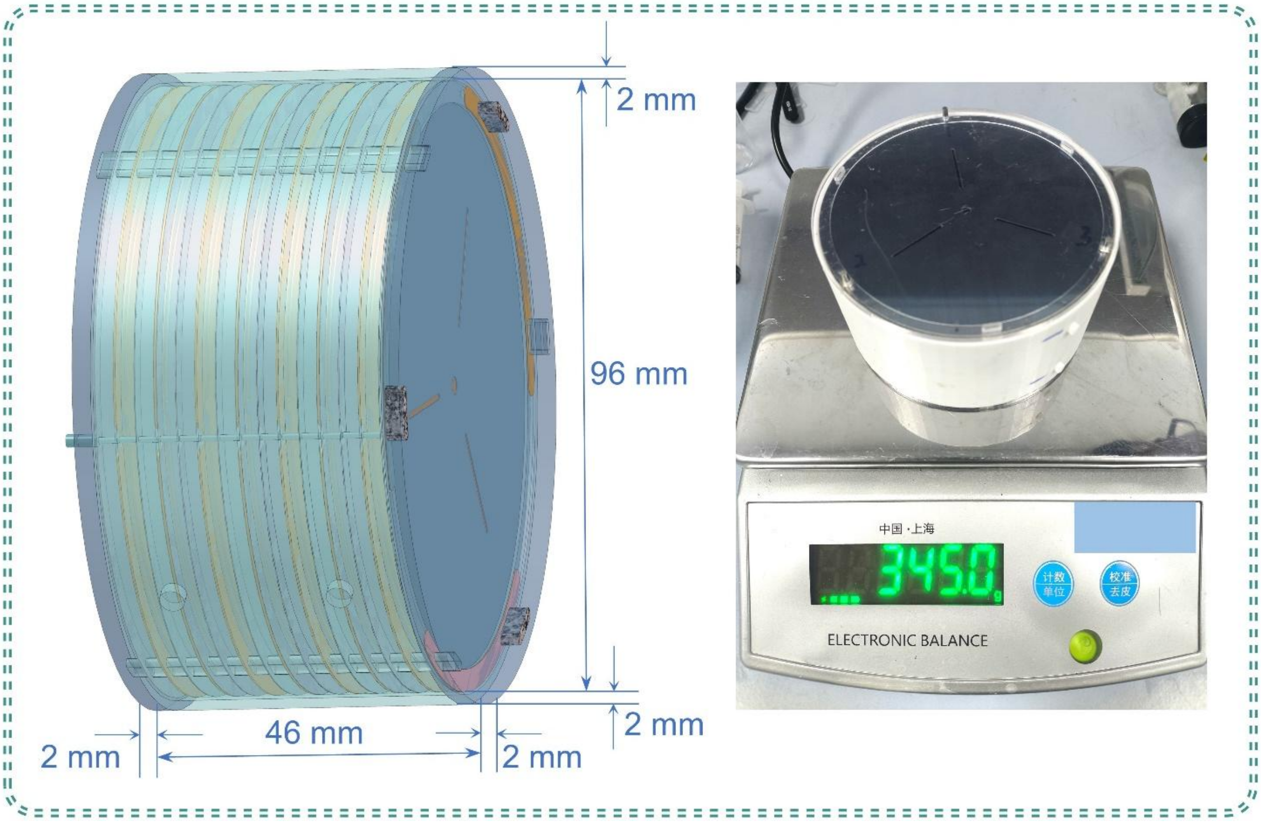

Supplementary Figure 24. Packaged device model parameters and physical image.

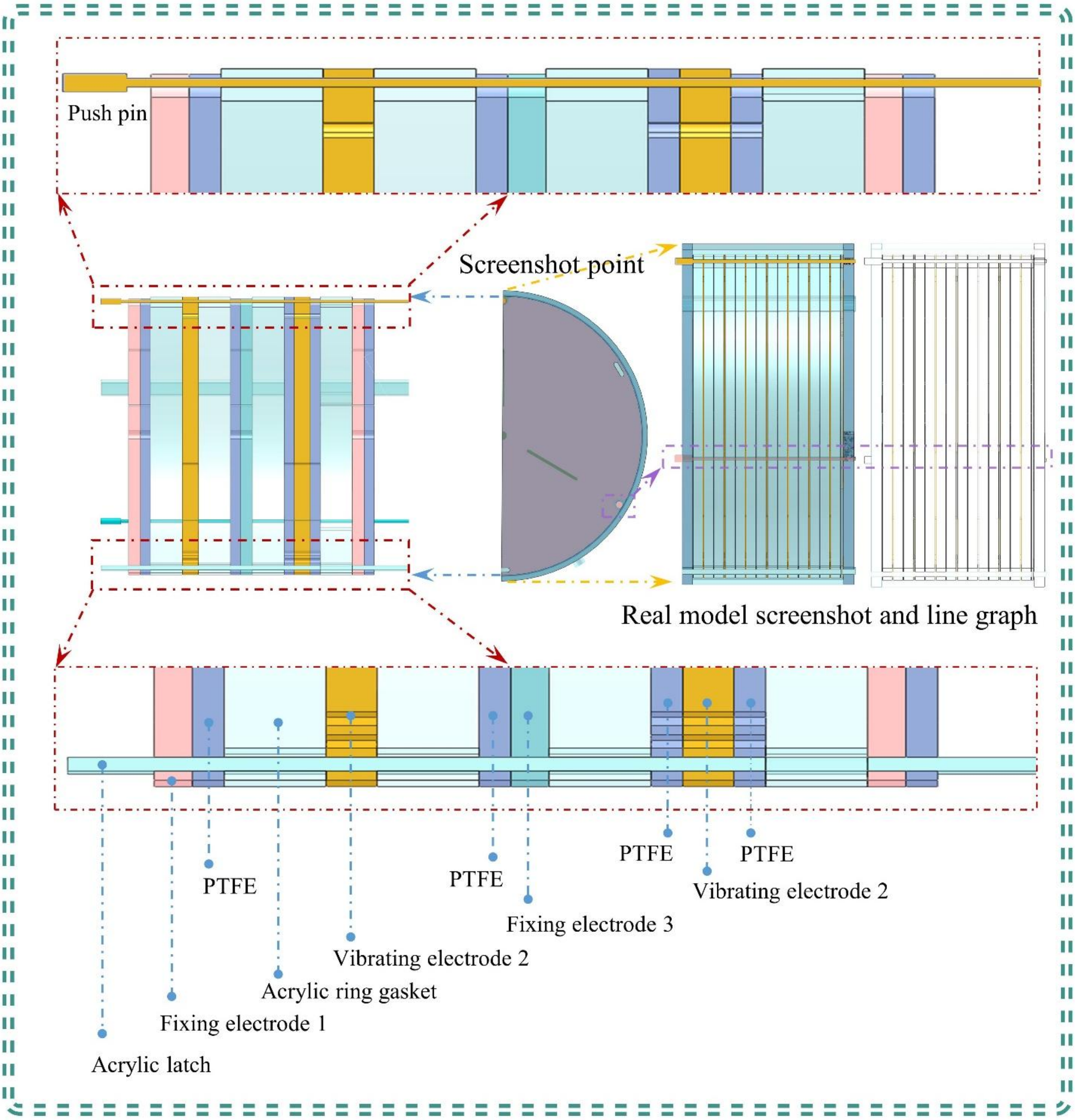

Supplementary Figure 25. Cross-sectional schematic and orientation diagram of a single structural cycle of the RD-TENG.

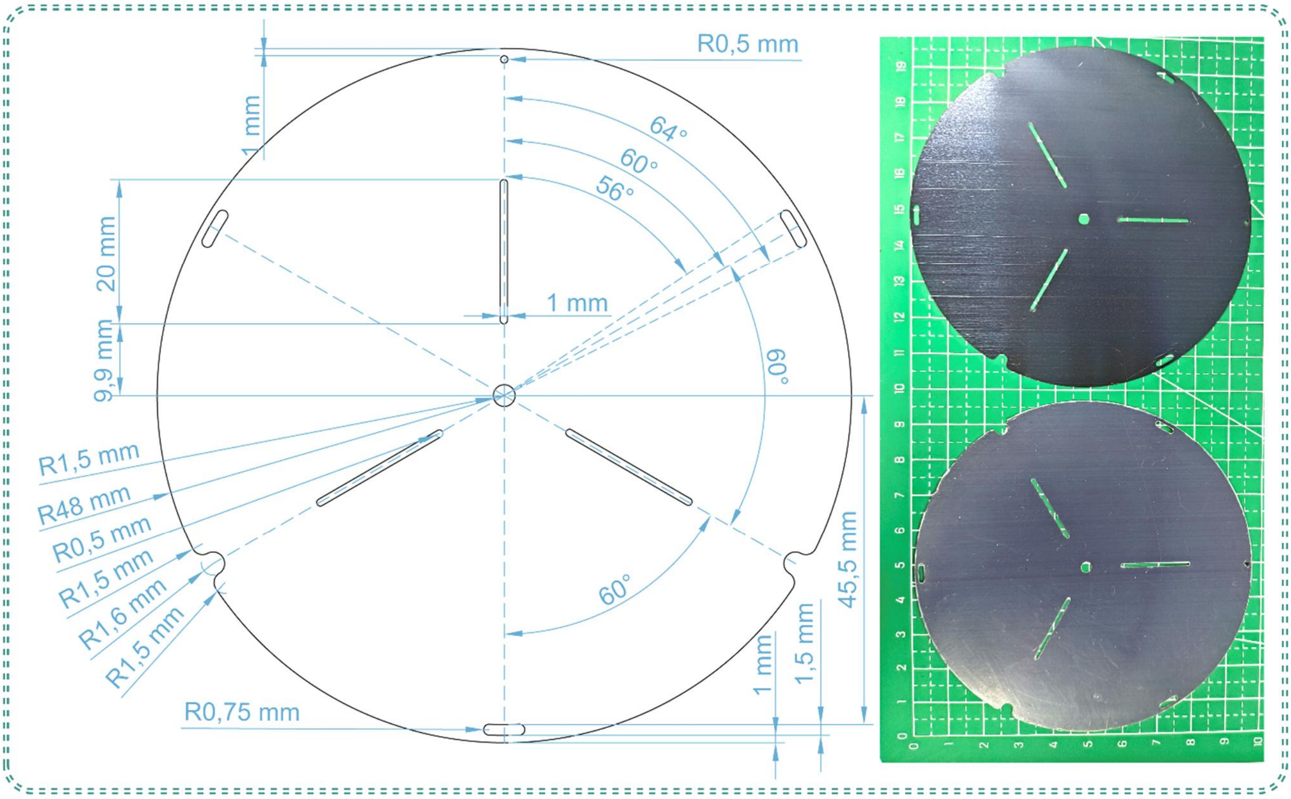

Supplementary Figure 26. Fixing sheet parameters and physical image.

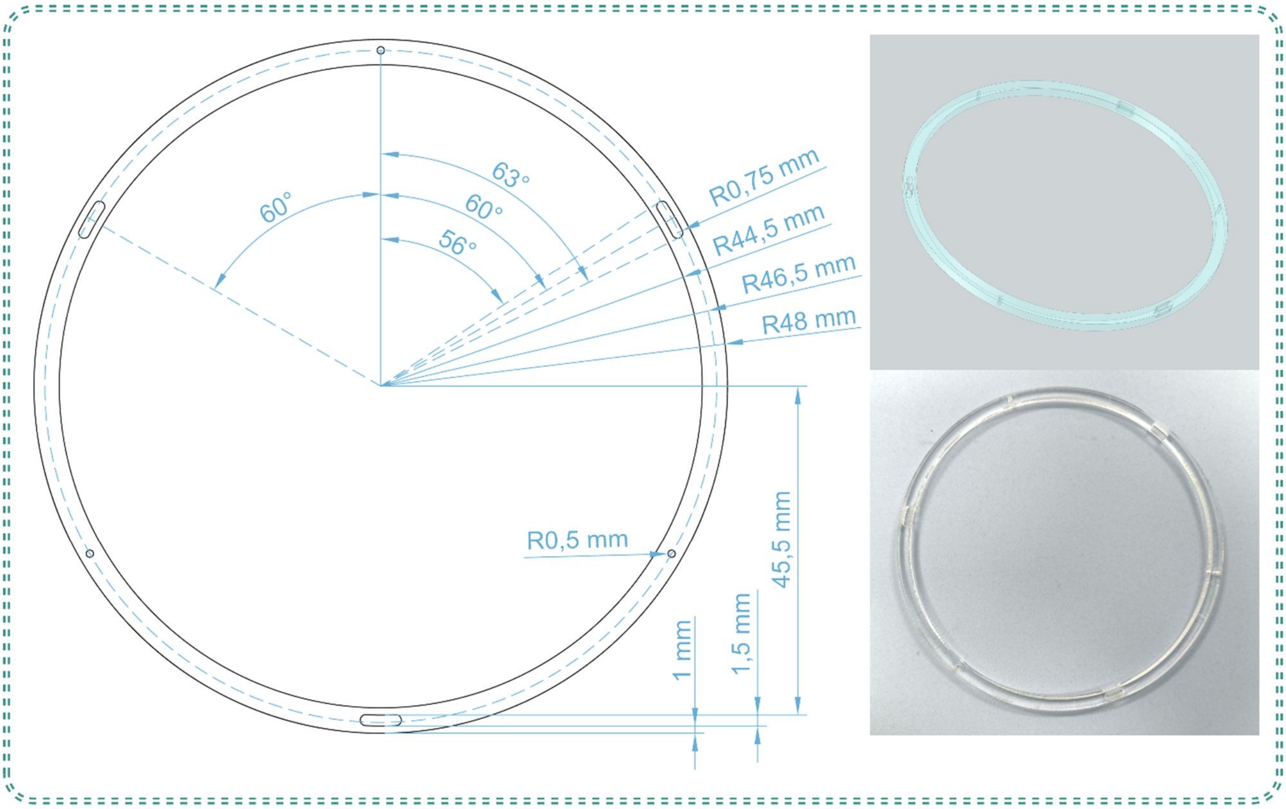

Supplementary Figure 27. Acrylic ring gasket parameters, model, and physical image.

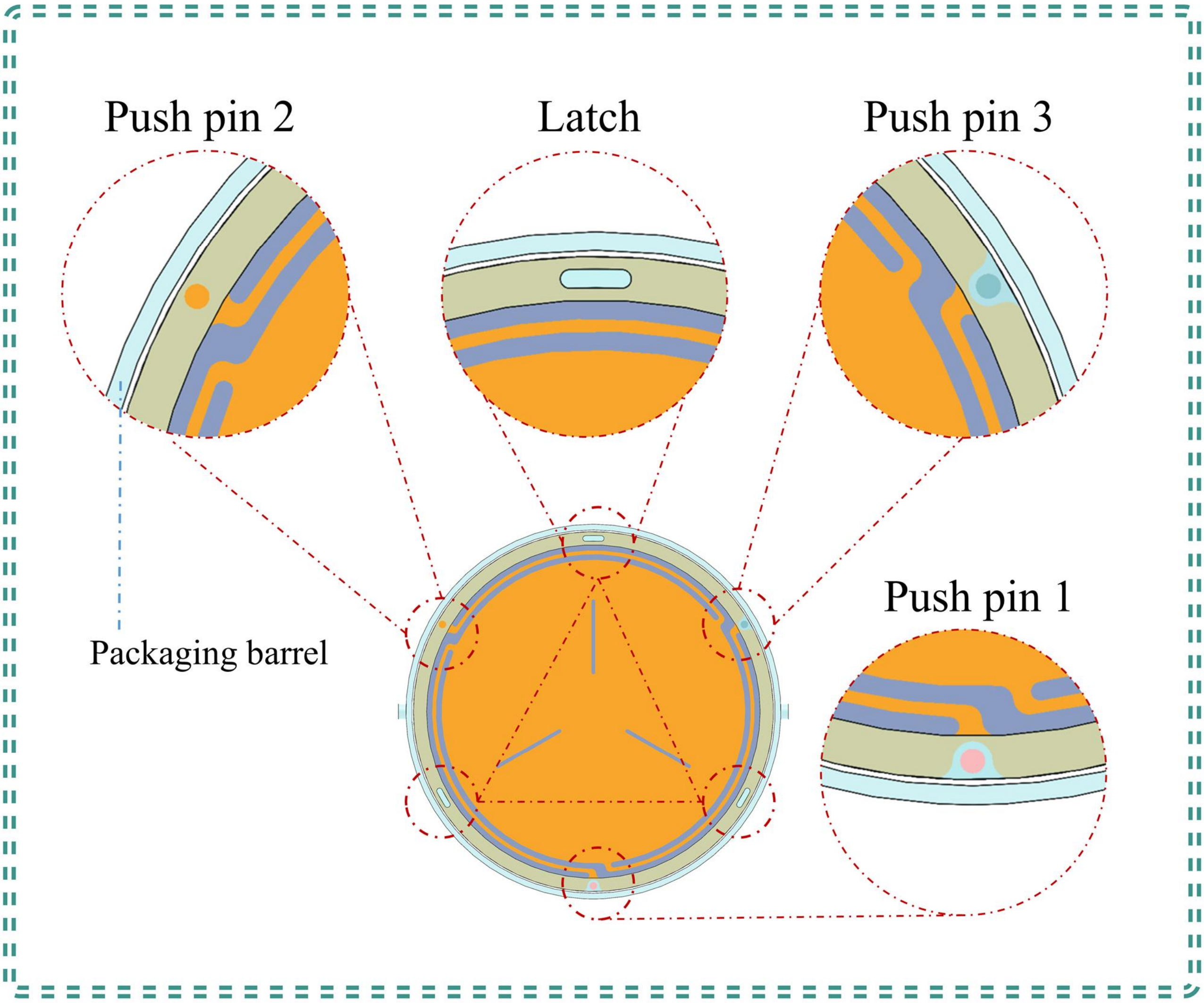

Supplementary Figure 28. Structural details of push pin and latch alignment holes.

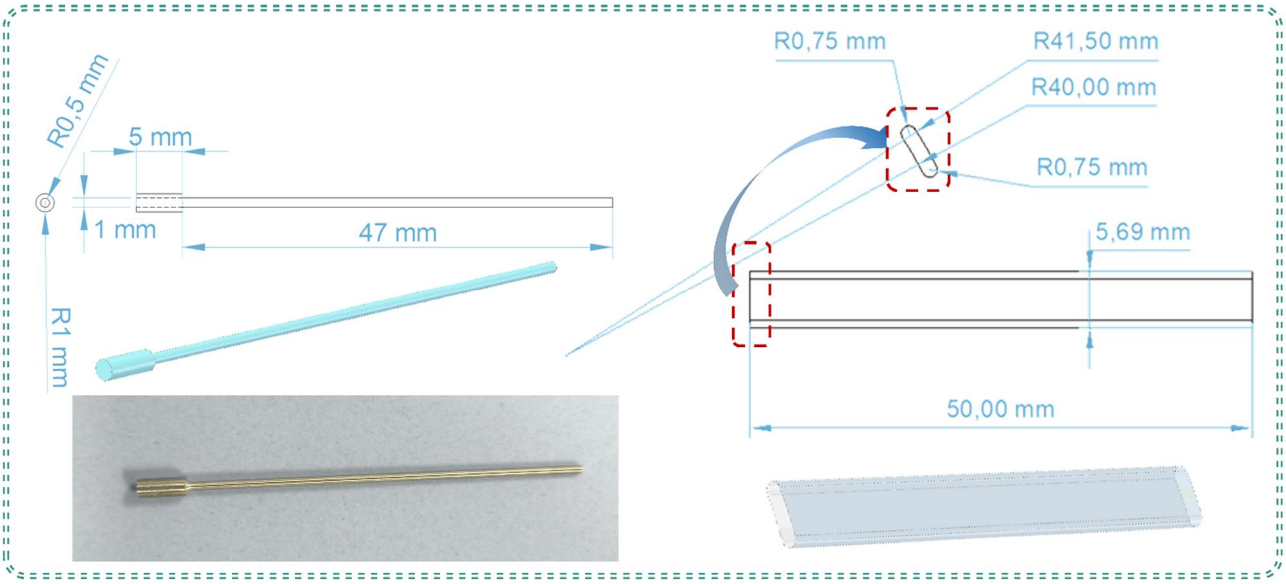

Supplementary Figure 29. Push pin and latch parameters.

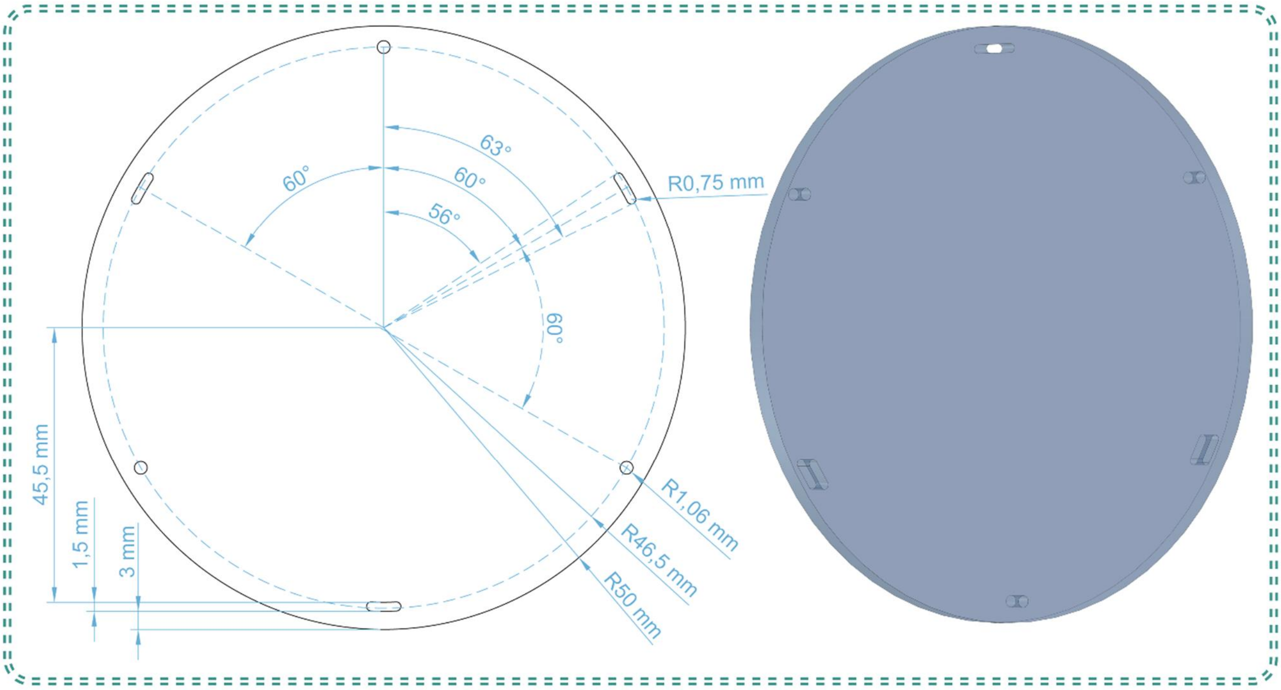

Supplementary Figure 30. Bottom sealing plate and its parameters.

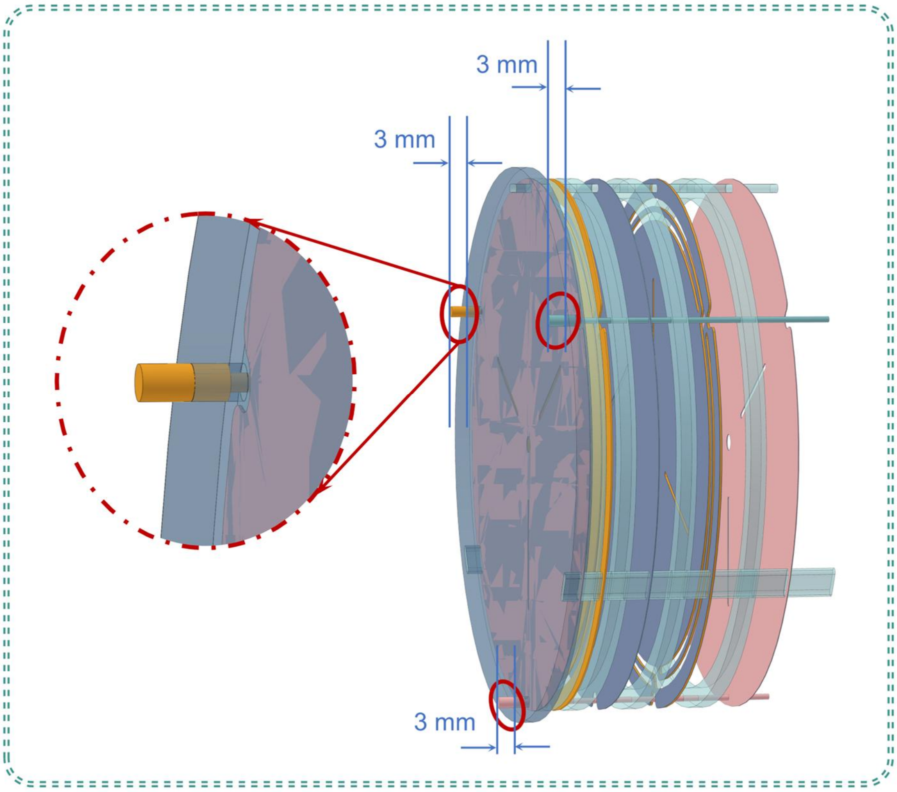

Supplementary Figure 31. The protruding end of the push pin serves as the electrode lead-out terminal.

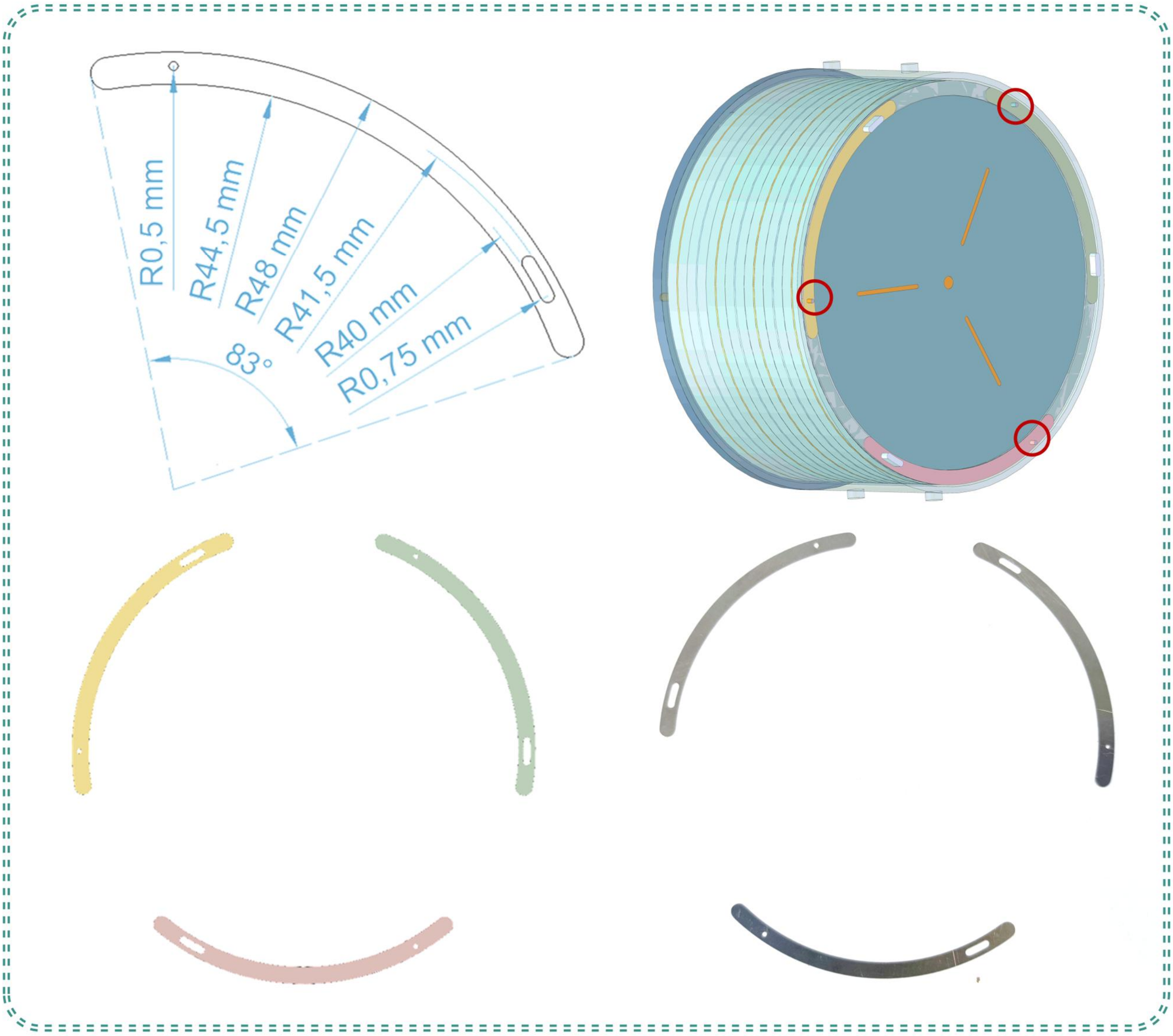

Supplementary Figure 32. Electrode conductive sheet parameters, position, model, and physical image.

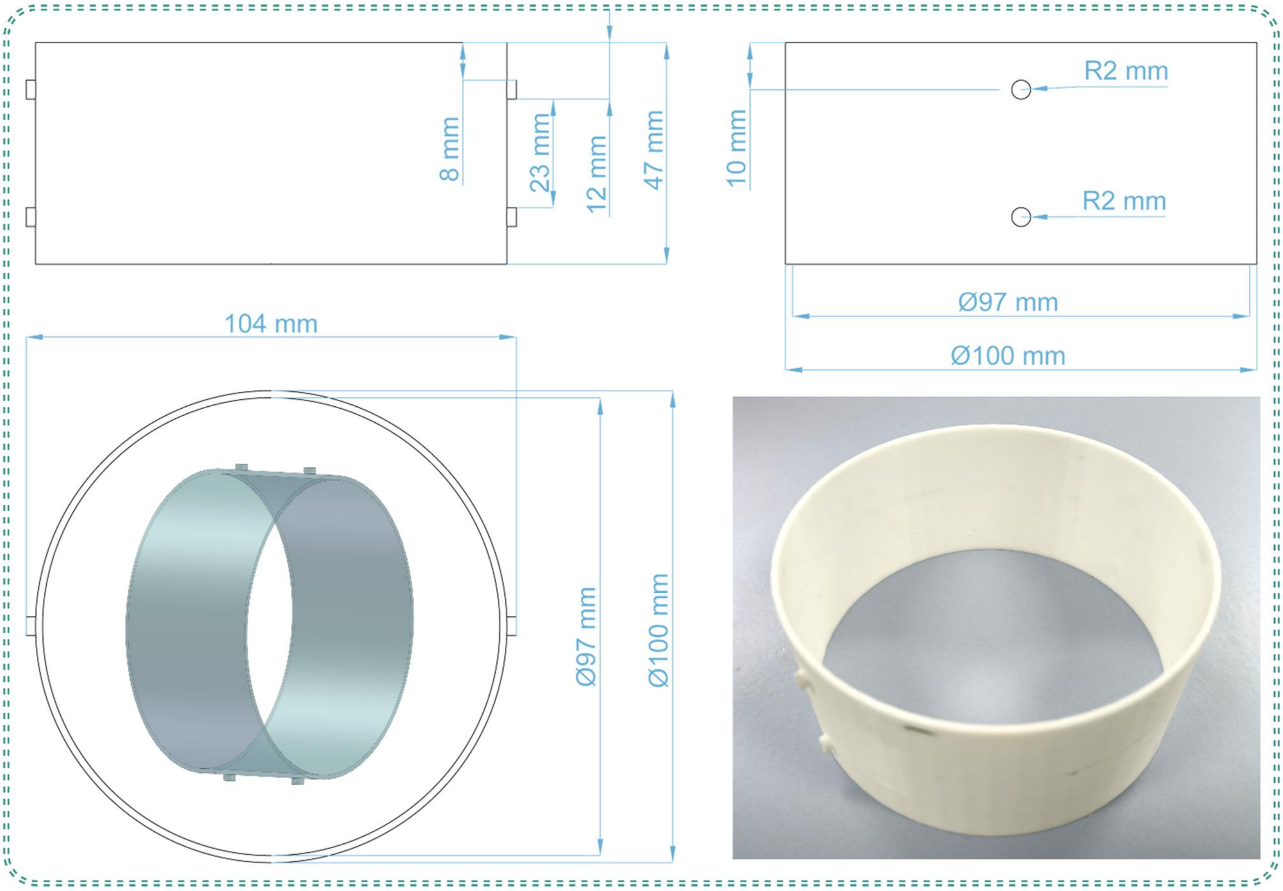

Supplementary Figure 33. Packaging barrel parameters and its physical image.

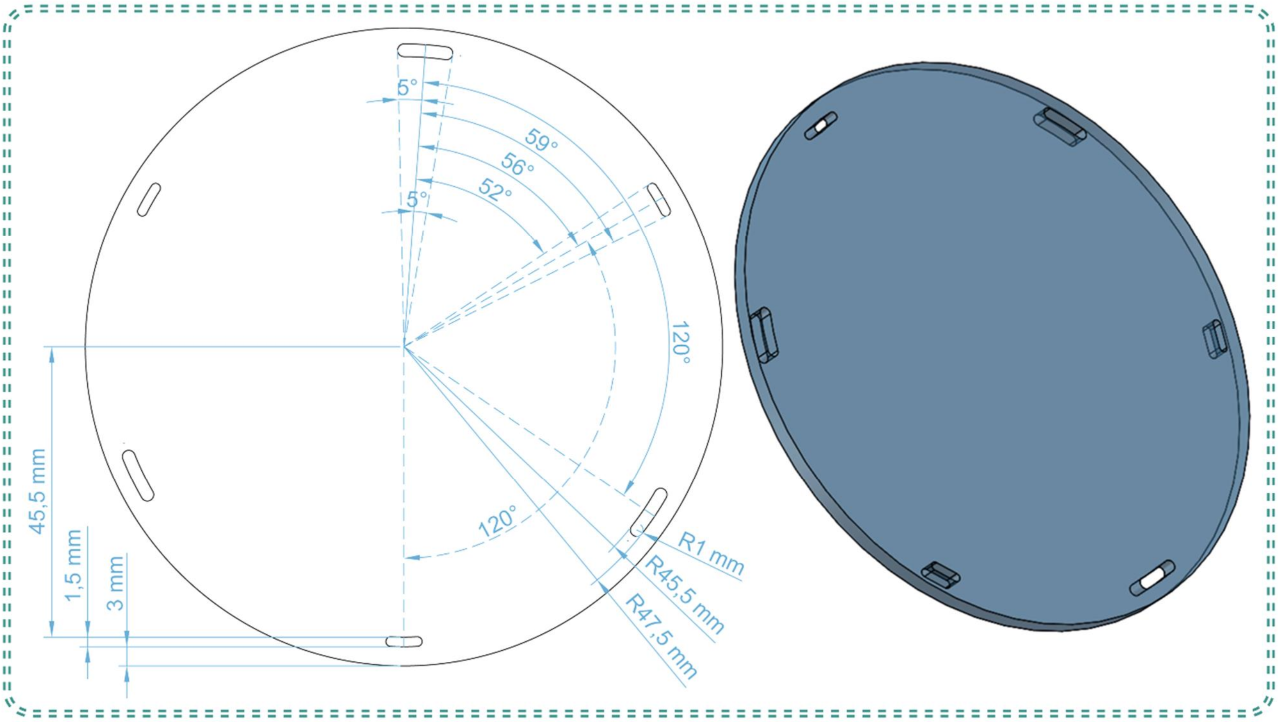

Supplementary Figure 34. Top sealing plate and its parameters.

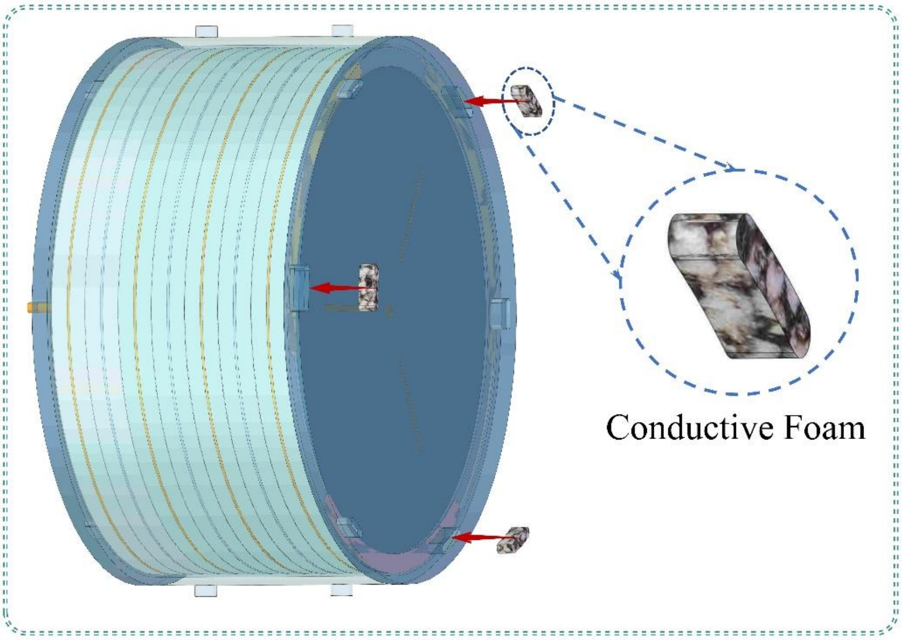

Supplementary Figure 35. Conductive sponge and its installation location.

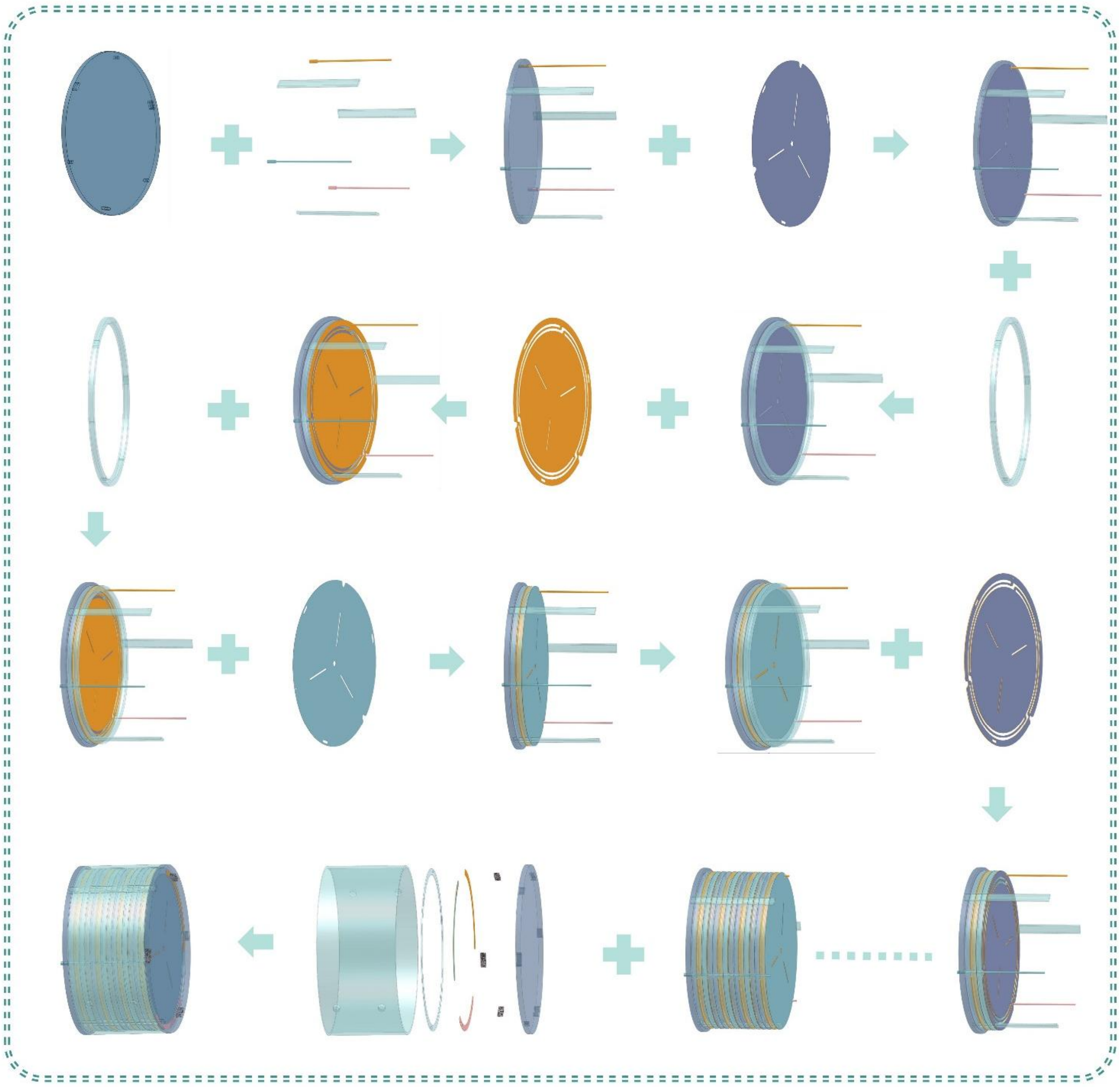

Supplementary Figure 36. Assembly steps of the RD-TENG device.

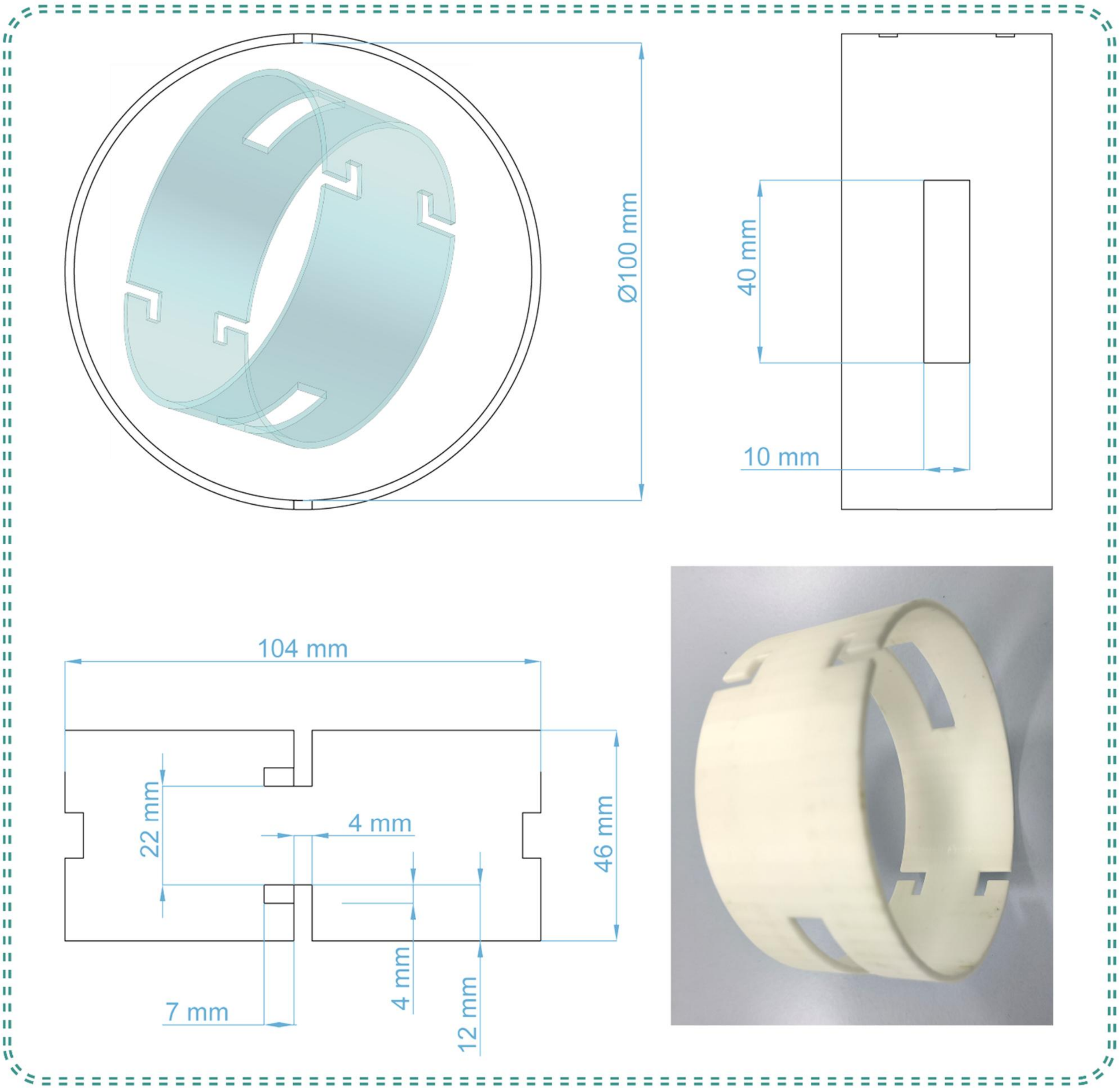

Supplementary Figure 37. Parameters and physical image of the rotating-clasp device.

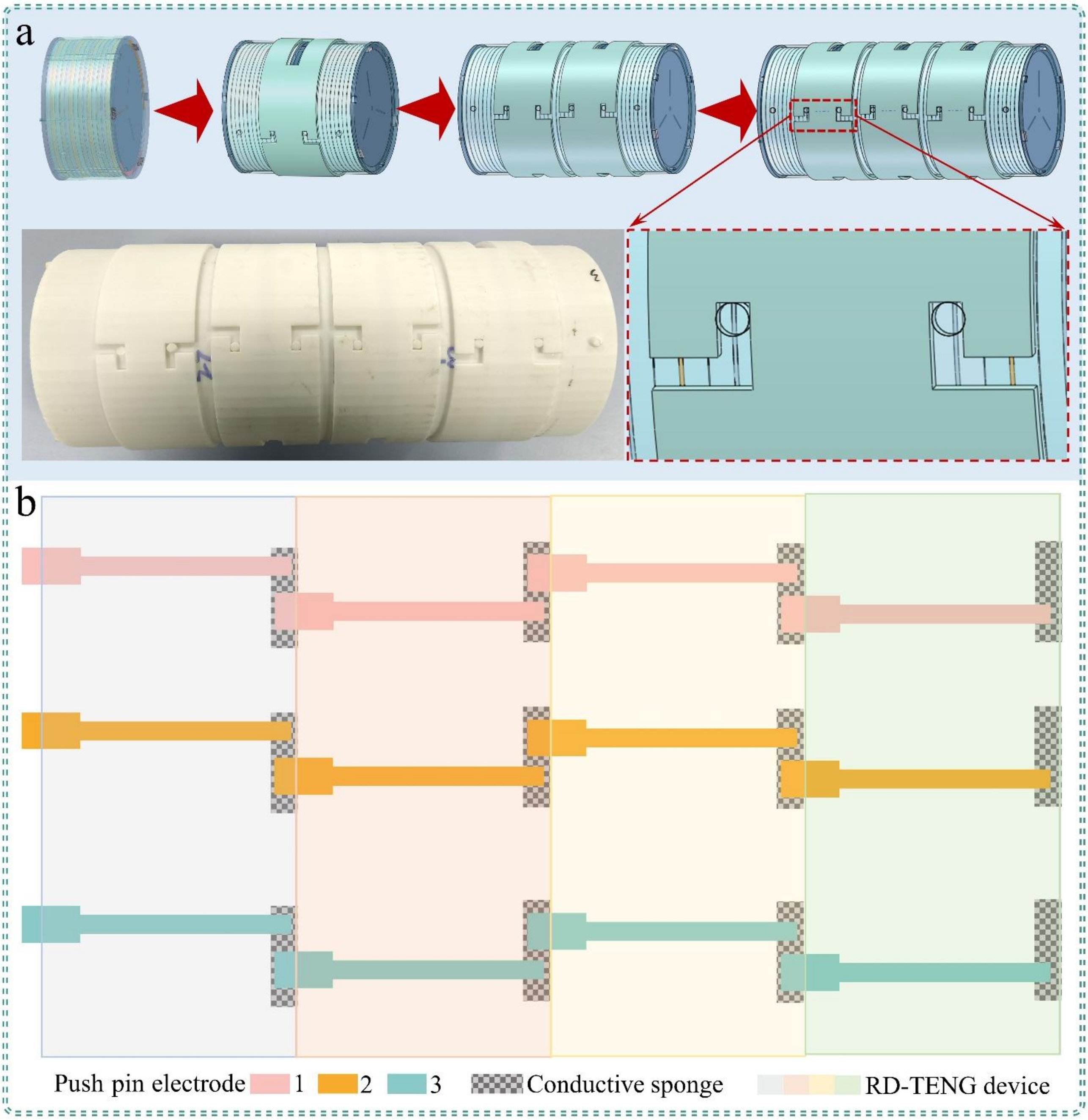

Supplementary Figure 38. (a) Details and physical image of the RD-TENG device array. (b) Connection method of push-pin electrodes in the device array.

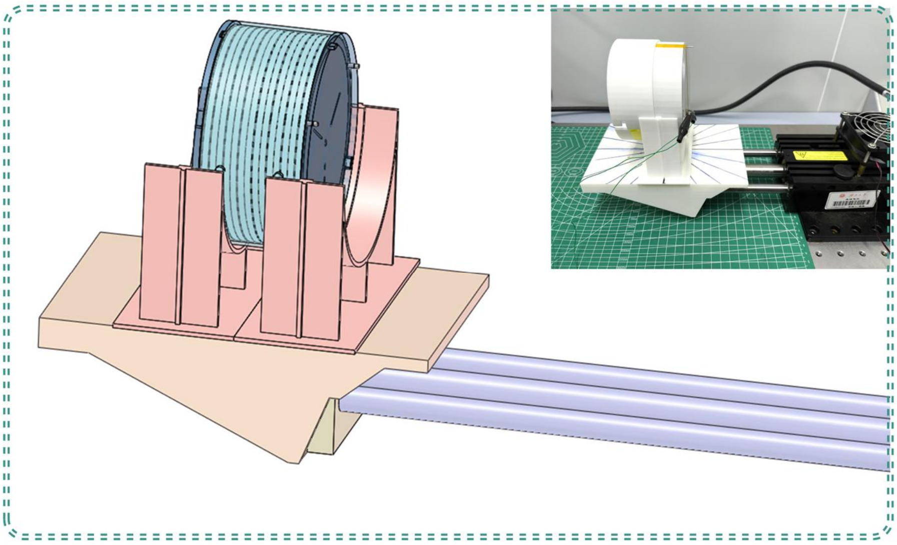

Supplementary Figure 39. Vibrational energy simulation testing platform.

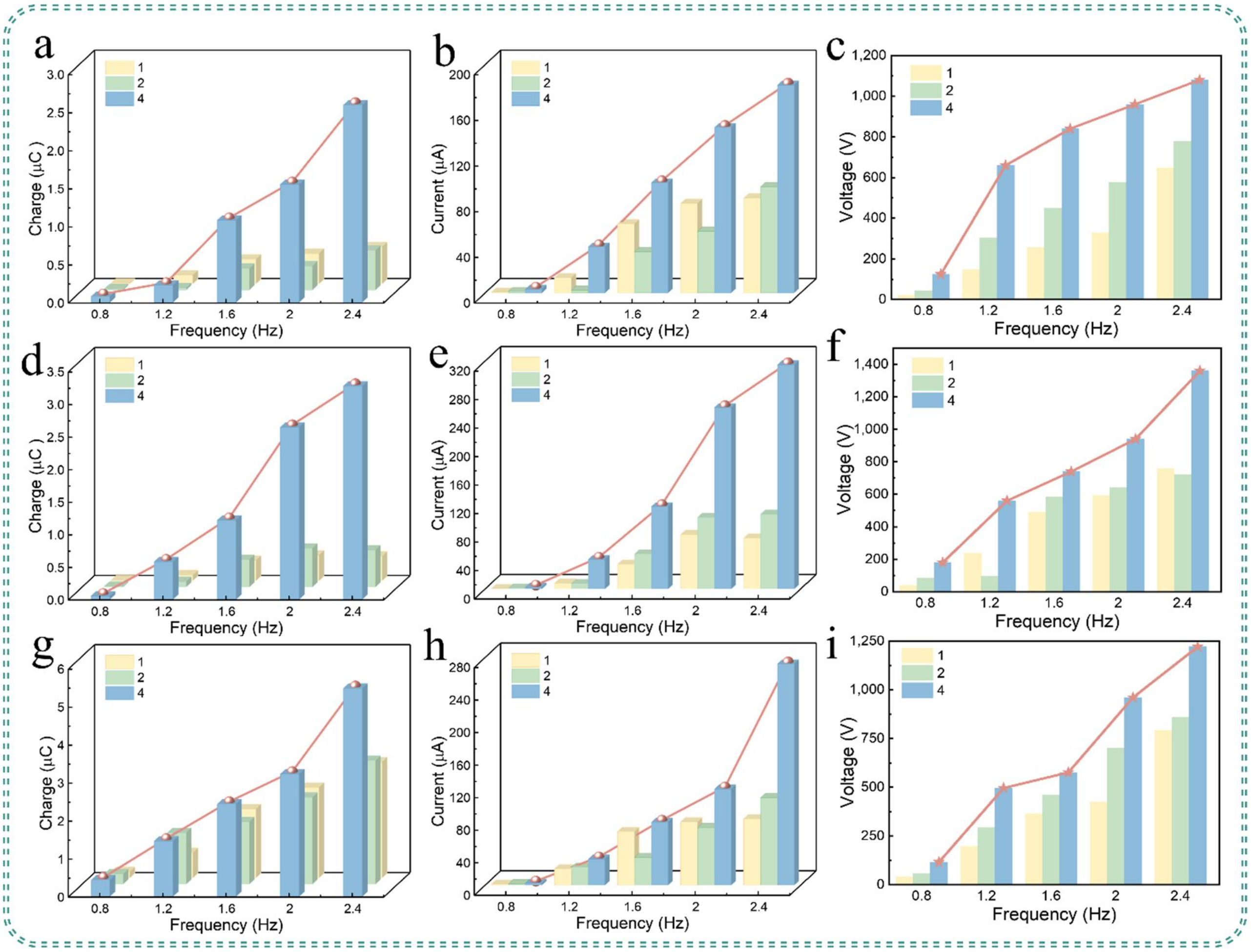

Supplementary Figure 40. Comparison of the output of RD-TENG (Structure 4) with traditional structures. (a-c) Comparison of output charge, current, and voltage for the 1-2 electrode pair across different structures. (d-f) Comparison of output charge, current, and voltage for the 2-3 electrode pair across different structures. (g-h) Comparison of output charge, current, and voltage for the rectified electrode pair across different structures.

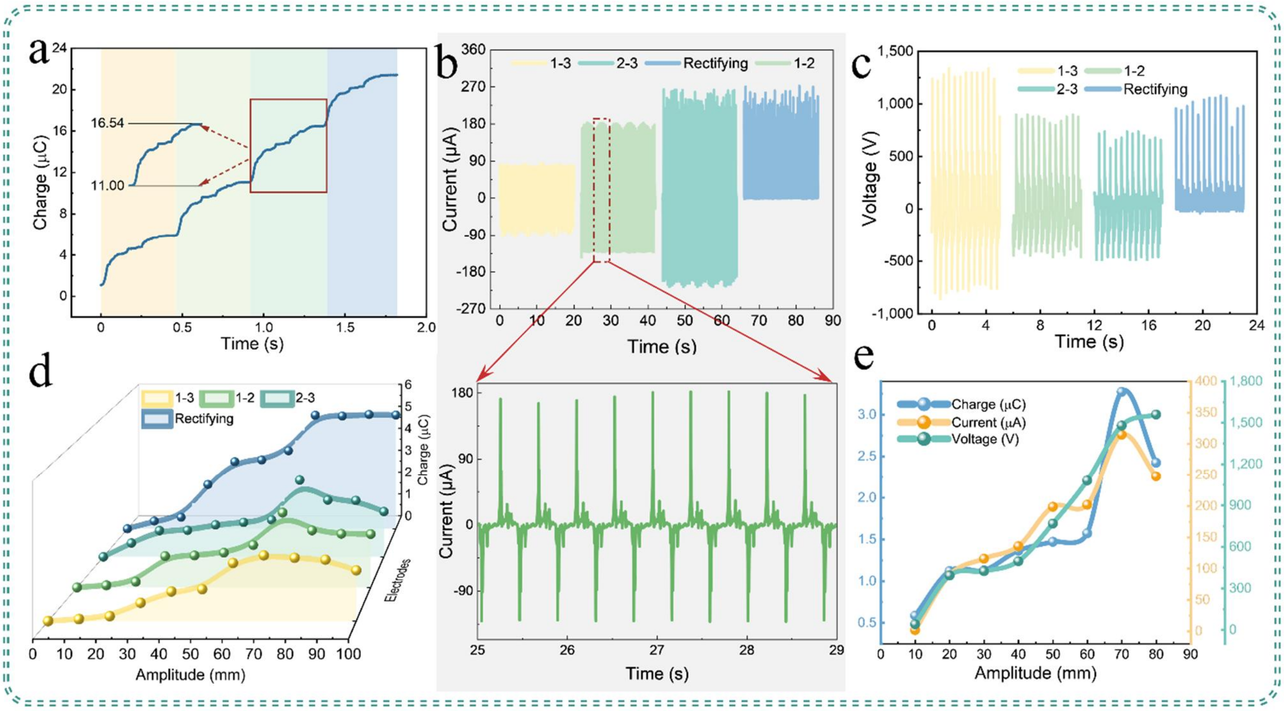

Supplementary Figure 41. Output characteristics of RD-TENG. (a) Cumulative charge of the rectified electrode pair of RD-TENG under optimal excitation conditions. (b-c) Short-circuit current and open-circuit voltage of each electrode pair. (d) The relationship between the charge output of each electrode pair and amplitude under a frequency of 2.4 Hz. (e) The output relationship between the output quantities of the 2-3 electrode pair and the amplitude under a frequency of 2.4 Hz.

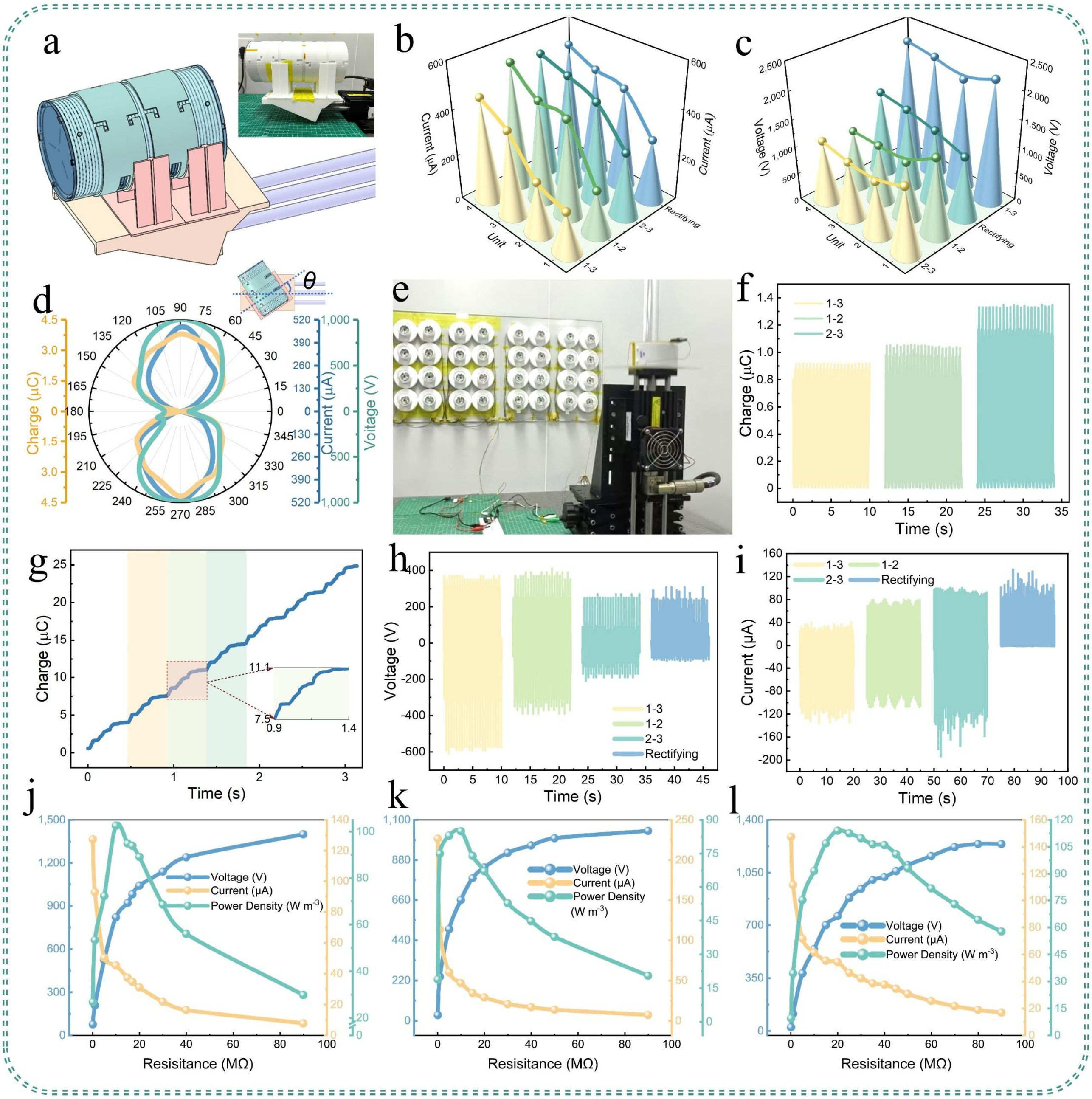

Supplementary Figure 42. Output characteristics of RD-TENG for harvesting vibrational energy. (a) Array device vibration energy simulation testing platform (b-c) Under the optimal excitation conditions of frequency 2.4 Hz and amplitude 70 mm, the output relationship of short-circuit current and open-circuit voltage with the increase in the number of device arrays. (d) The output relationship of the 1-2 electrode pair for three devices within the array under optimal excitation conditions concerning the variation of azimuth angle. (e-i) Vertical test platform and short-circuit transfer charge, open-circuit voltage, and short-circuit current of each electrode pair in vertical testing. (j-l) Output voltage, current, and peak power density of RD-TENG with different load resistances for the 1-2, 2-3, and rectified electrode pairs.

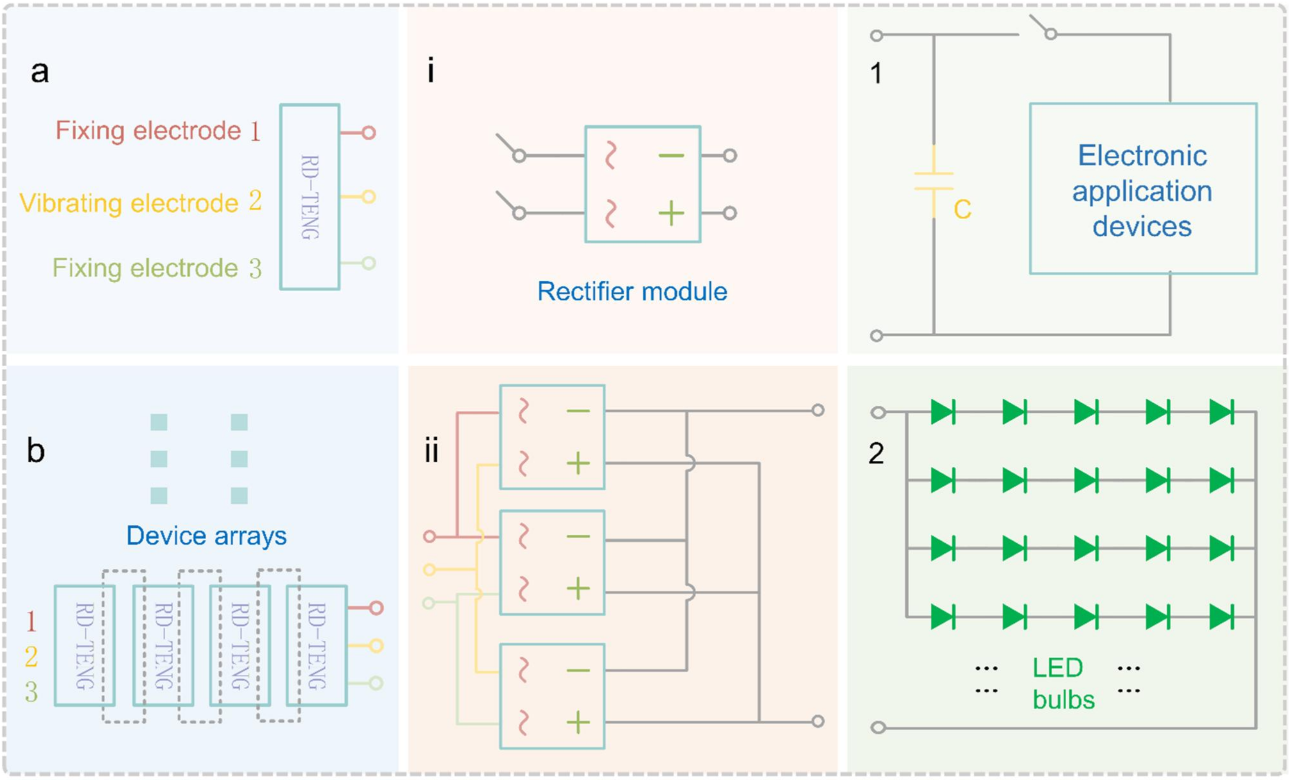

Supplementary Figure 43. Various circuit matching modules used in different tests, adaptable to specific application scenarios.

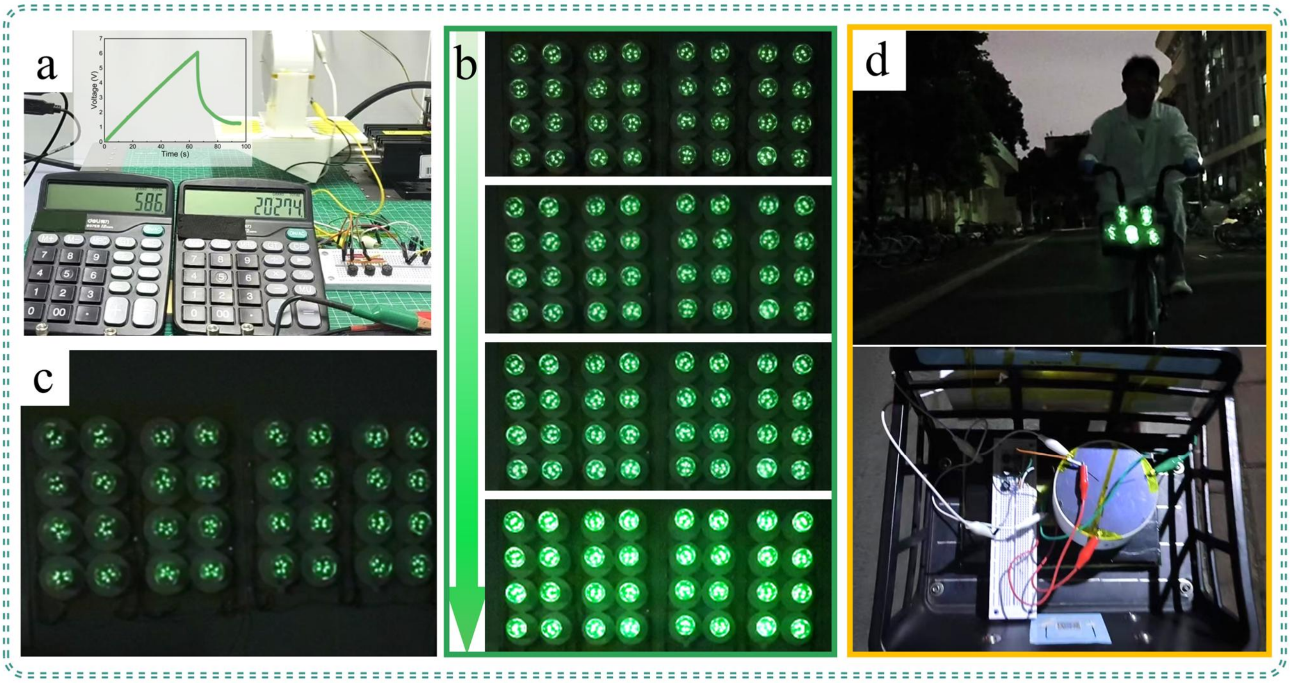

Supplementary Figure 44. Application of RD-TENG for vibration energy harvesting. (a) One RD-TENG device charges a 100 μF capacitor for 100 seconds, drives two parallel calculators with a continuous power supply for 15 seconds. (b) Schematic diagram showing the lighting of 32 2 W LED bulbs from dim to bright by adding 1 to 4 devices in sequence in the array under the optimal combination of 2.4 Hz frequency and 70 mm amplitude. (c) RD-TENG illuminates 32 2W LED bulbs under vertical drive. (d) The rectifier electrode pair of a single RD-TENG device is placed in the bicycle front basket, driving five 2W commercial LED bulbs as a front light or warning light for the bicycle, providing safety warnings for night rides.

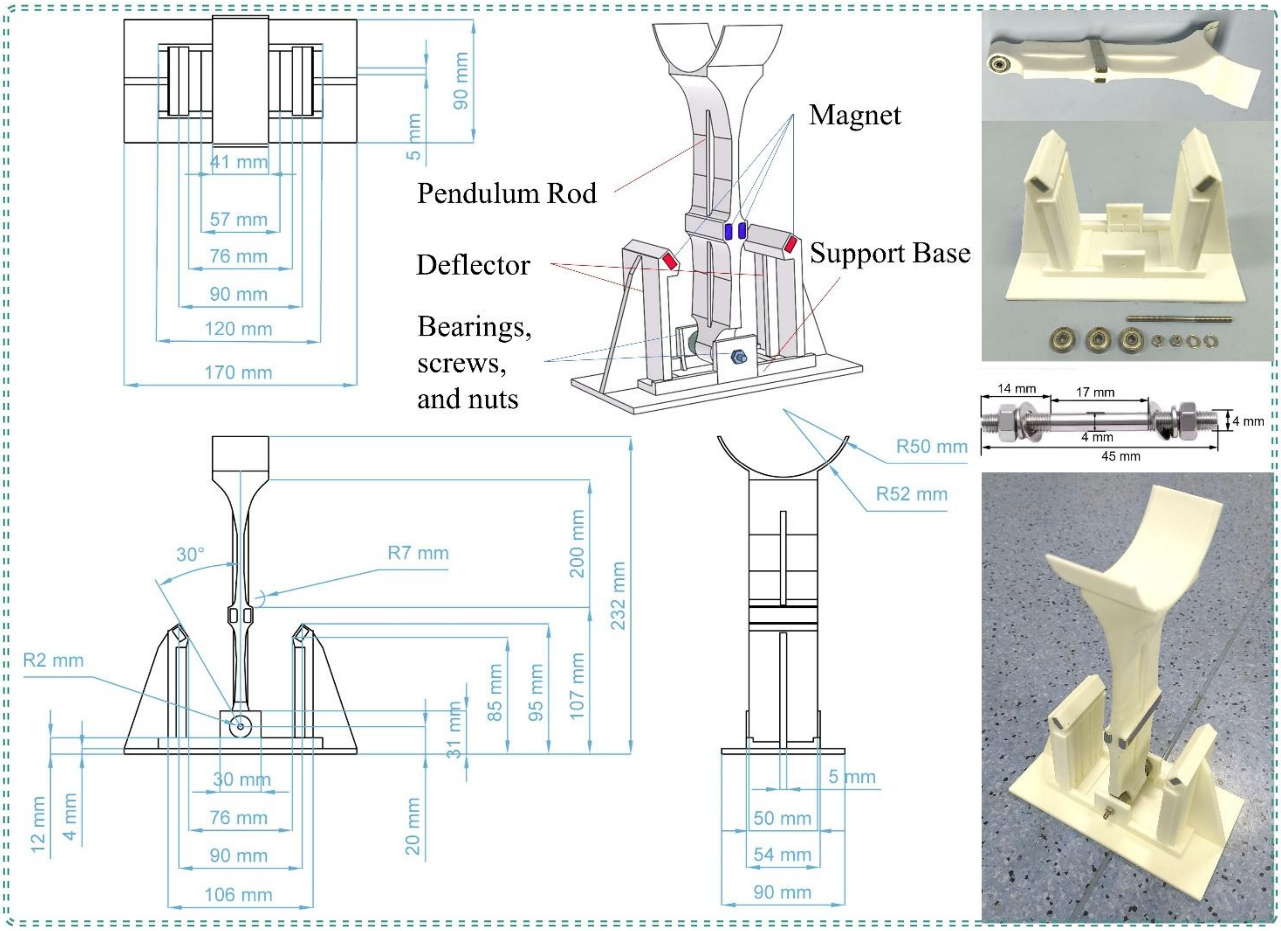

Supplementary Figure 45. Dimensional parameters and schematic diagram of the components of the frequency-reducing and amplitude-amplifying magnetic repulsion pendulum.

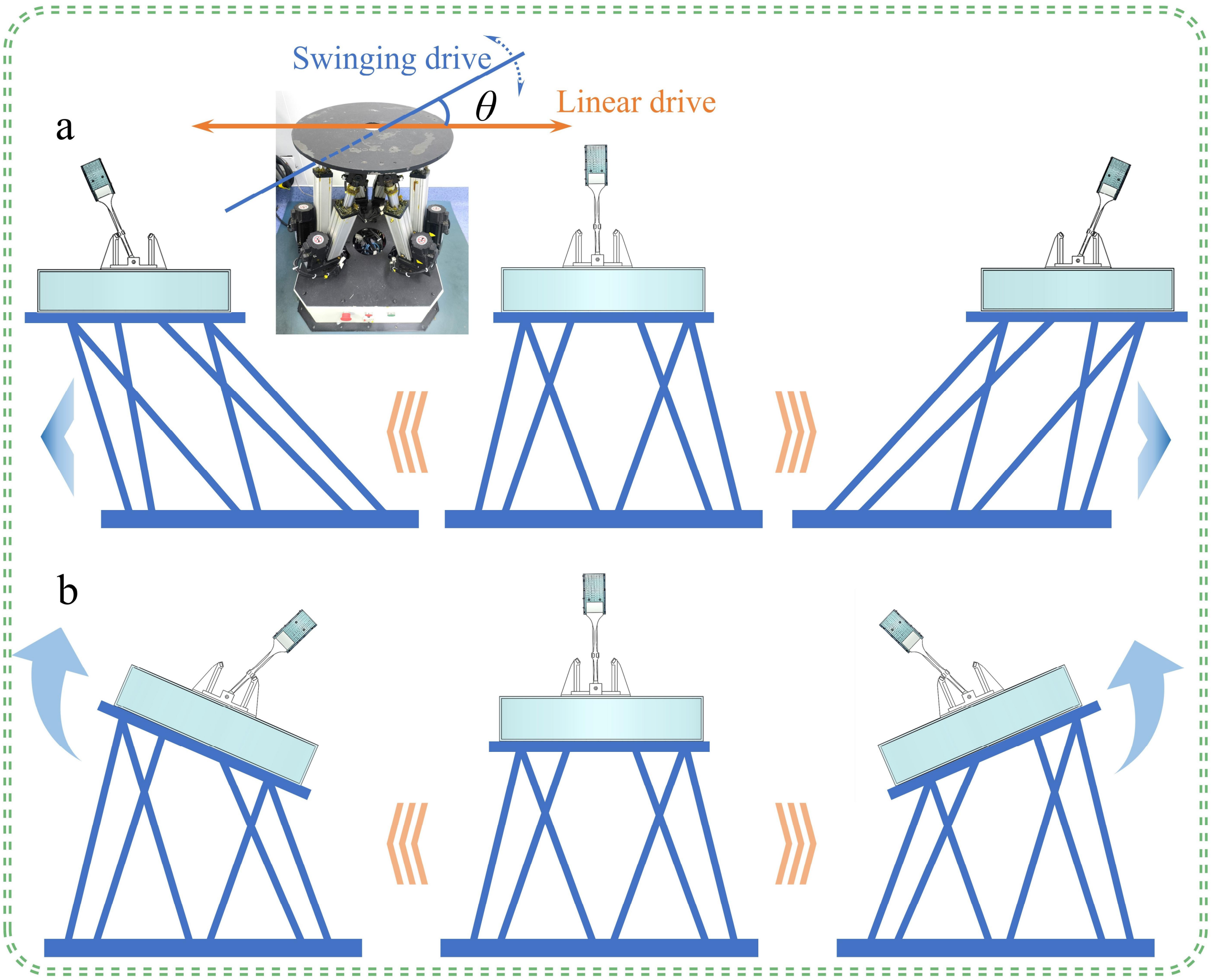

Supplementary Figure 46. RD-TENG based on frequency-reducing and amplitude-amplifying magnetic repulsion pendulum driven by a six-degree-of-freedom platform. (a) linear drive. (b) swinging drive.

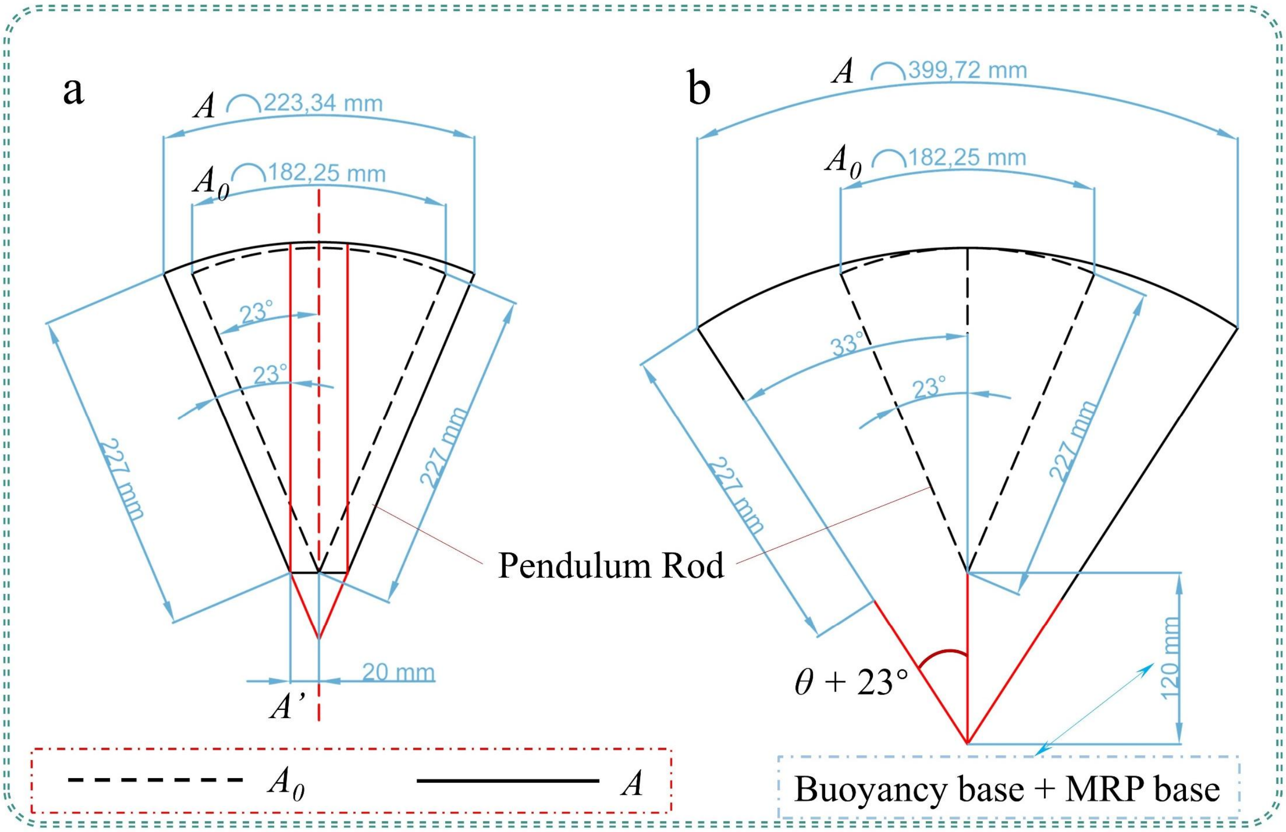

Supplementary Figure 47. Amplitude amplification calculation diagram of frequency-reducing and amplitude-amplifying magnetic repulsion pendulum under linear and swinging drive. (a) linear drive (A'=20 mm). (b) swinging drive (θ=10°). Here, A' is the driven amplitude of the base, A_0 is the amplitude of the MRP, A is the amplitude of the RD-TENG, and θ is the phase angle of the driven base.

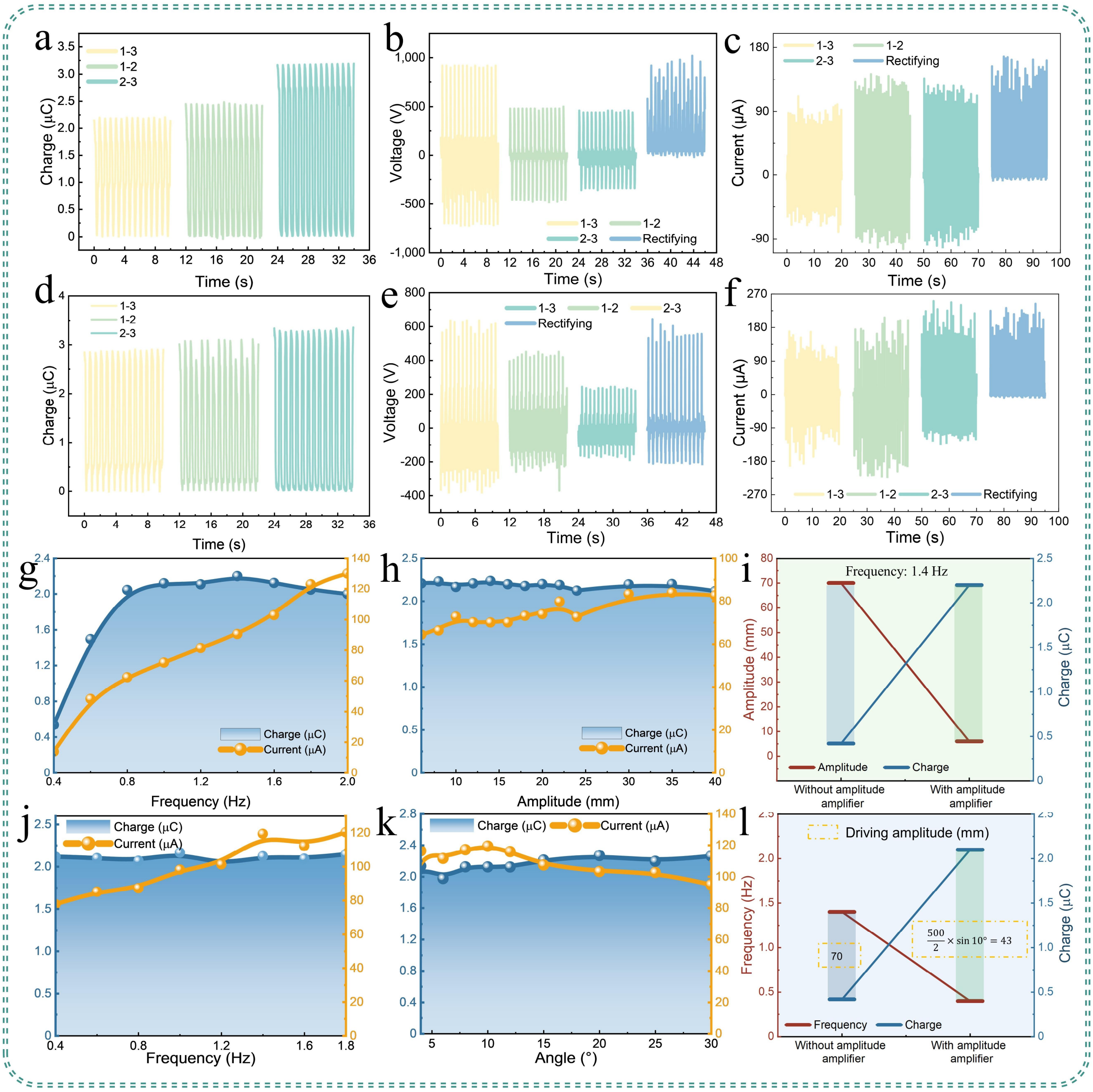

Supplementary Figure 48. Output characteristics of RD-TENG based on frequency-reducing and amplitude-amplifying magnetic repulsion pendulum under linear drive and swing drive on a six-degree-of-freedom platform. (a-c) Short-circuit transferred charge, open-circuit voltage, and short-circuit current of each electrode pair under linear drive. (d-f) Short-circuit transferred charge, open-circuit voltage, and short-circuit current of each electrode pair under swinging drive. (g-h) Relationship between output quantities and frequency (Fixed at an amplitude of 70 mm), amplitude (Fixed at a frequency of 2.4 Hz) under linear drive. (i) Frequency reduction and amplitude amplification characteristics under linear drive. (j-k) Relationship between output quantities and frequency (Fixed at an amplitude of 70 mm), amplitude (Fixed at a frequency of 2.4 Hz) under swinging drive. (l) Frequency reduction and amplitude amplification characteristics under swinging drive (The diameter of the buoyant base is 500 mm).

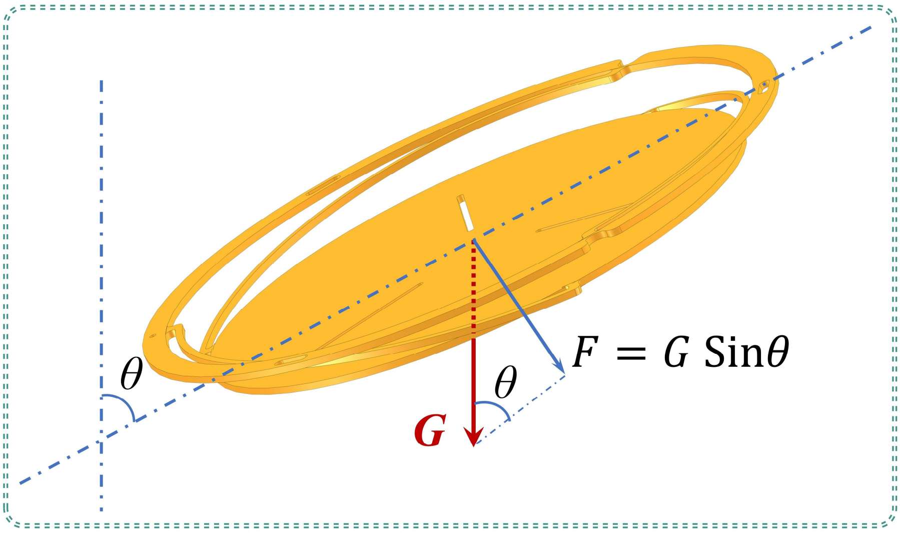

Supplementary Figure 49. Force analysis diagram of the vibrating sheet under swinging drive, where a larger swing angle allows the rotor to open more effectively. *G* denotes the gravity of the vibrating sheet oscillator, θ represents the sum of the base swing angle and the MRP pendulum rod swing angle, and *F* denotes the force acting on the oscillator along the displacement direction.

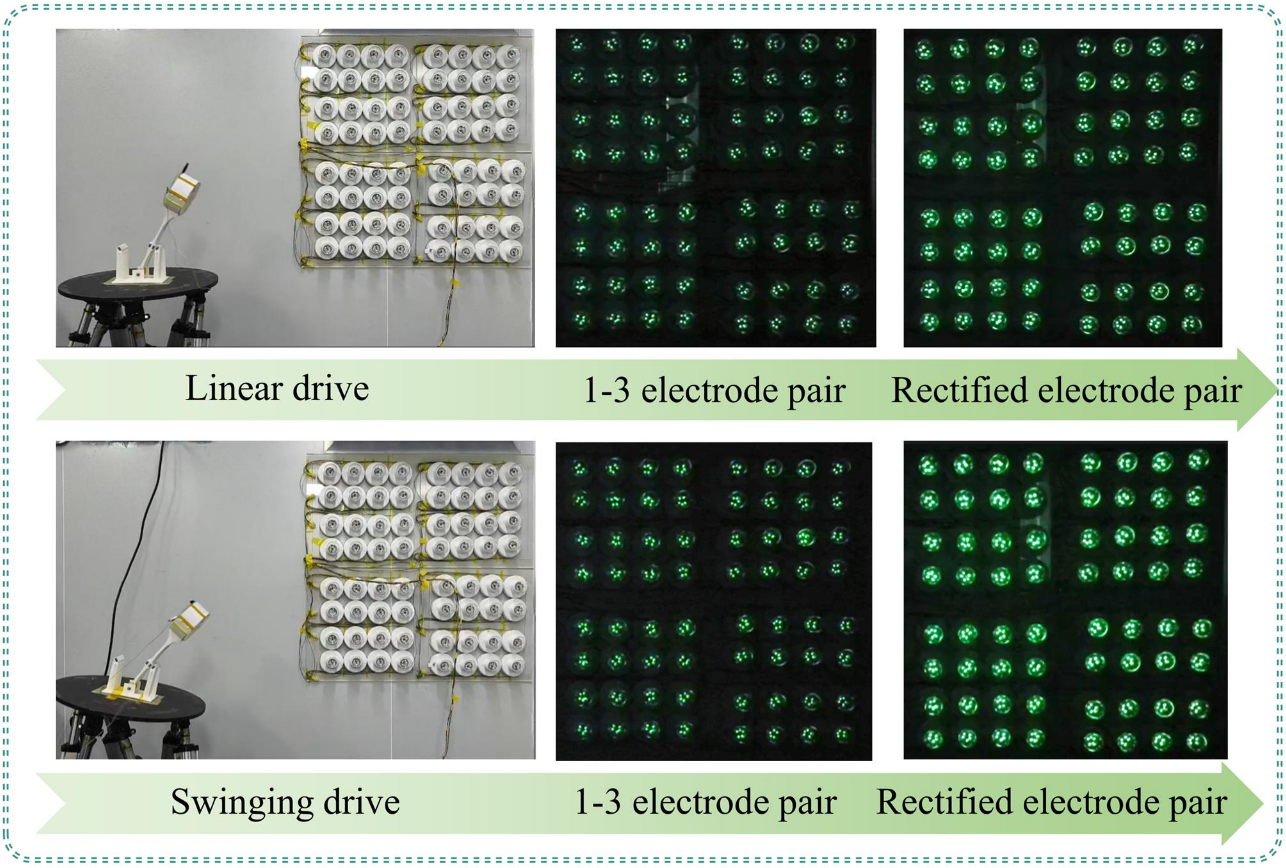

Supplementary Figure 50. Comparison of 1-3 and rectified electrode pair lighting up 64 2 W LED bulbs in linear drive and swinging drive mode of RD-TENG based on the frequency-reducing and amplitude-amplifying magnetic repulsion pendulum.

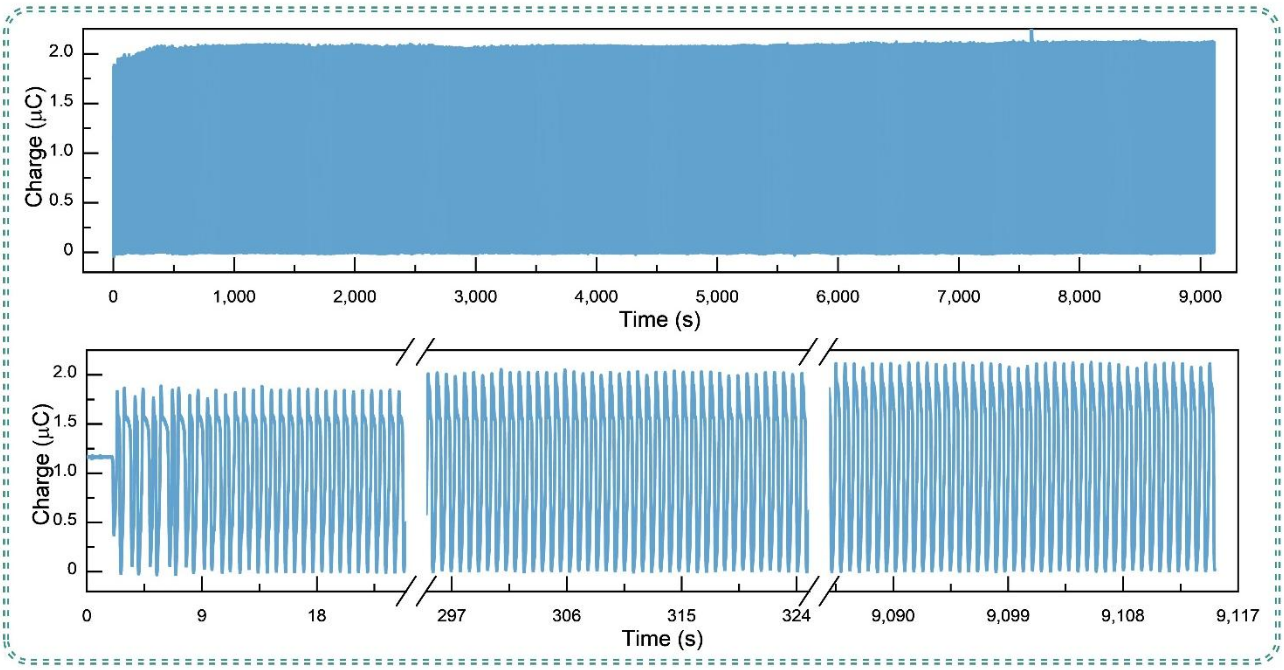

Supplementary Figure 51. Durability test of the 1-3 electrode pair of RD-TENG based on low-frequency and amplitude-amplifier of magnetic repulsive pendulum under linear excitation.

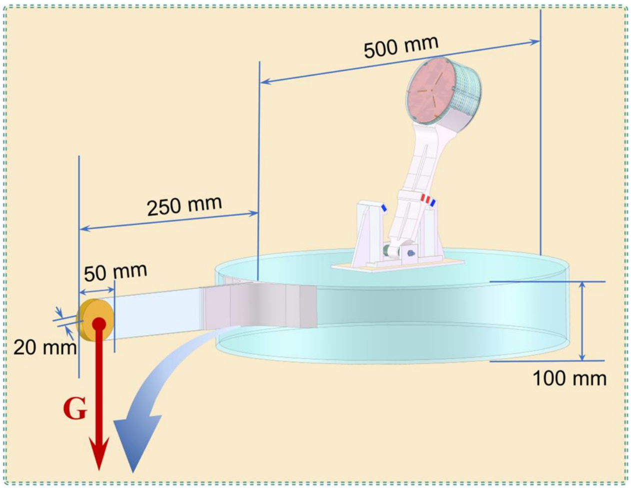

Supplementary Figure 52. Diagram of the unbalanced gravity pendulum set on the edge of a buoyant base.

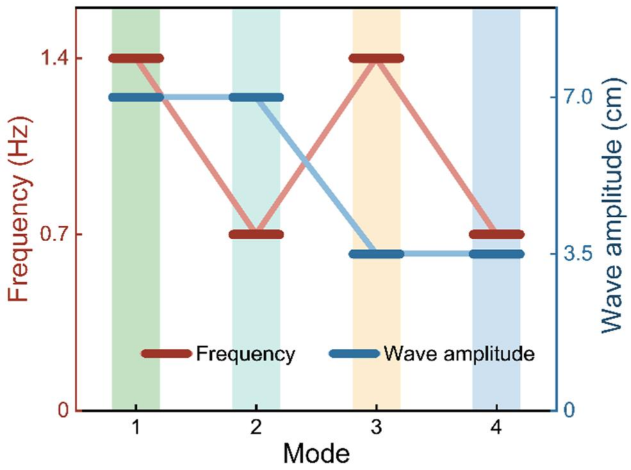

Supplementary Figure 53. Wave amplitudes and frequencies generated by different modes in a wave pool.

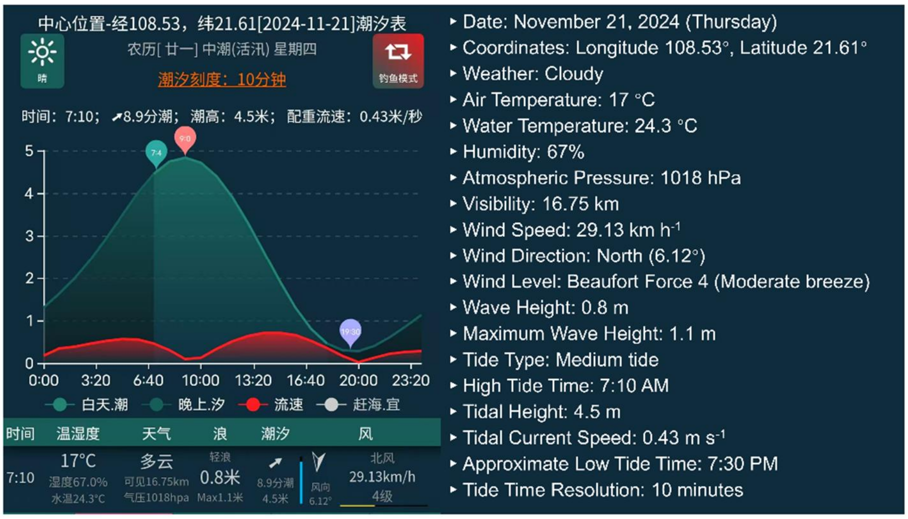

Supplementary Figure 54. Screenshot of meteorological and sea conditions at the beginning of data acquisition during real-sea testing.

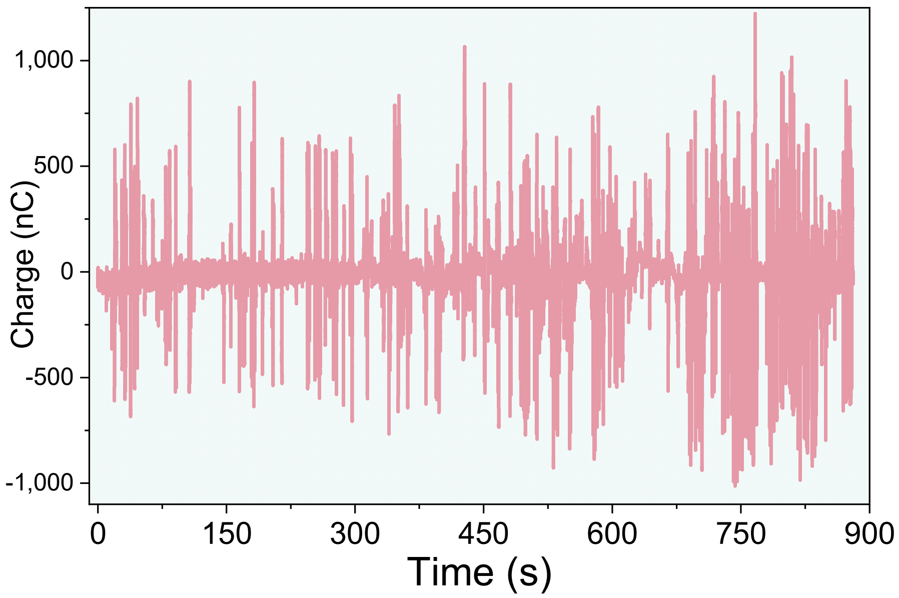

Supplementary Figure 55. Real-sea output performance of the 1-3 electrode pair, showing no structural failure during the 14-minute testing period.

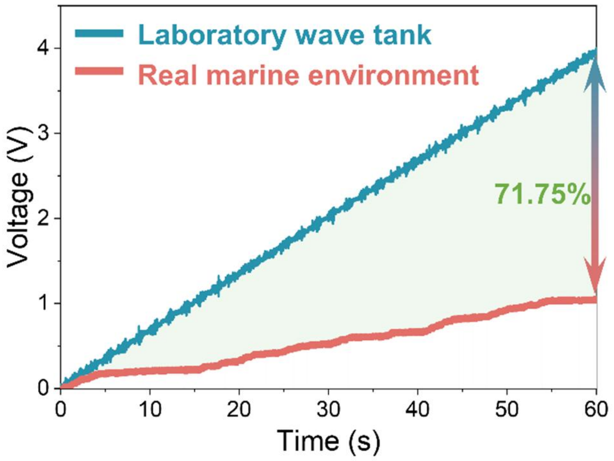

Supplementary Figure 56. Comparison of capacitive energy storage efficiency between real-sea condition and optimal condition on the six-degree-of-freedom simulation platform.

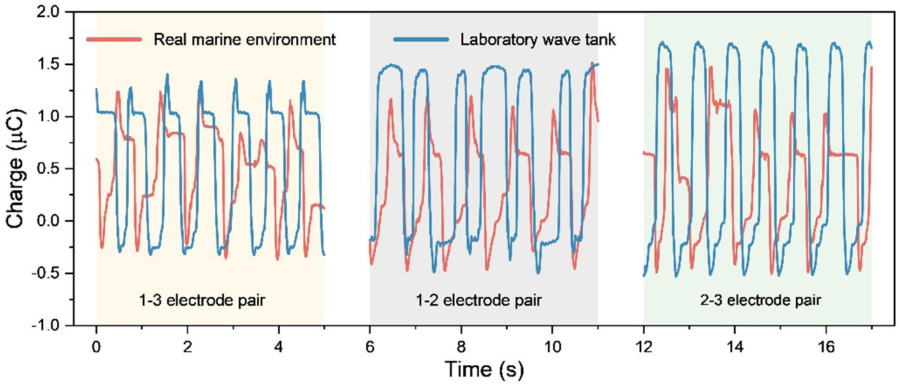

Supplementary Figure 57. Comparison of output data under real-sea conditions and laboratory wave tank mode 1 conditions
