# Supplementary material for: Rattle drum-inspired triboelectric nanogenerator with enhanced output using charge dispatch and magnetic repulsion pendulum
Source: Nat Commun. 2025 Oct 29;16:9539. doi: 10.1038/s41467-025-64575-9 (PMC12572158; doi:10.1038/s41467-025-64575-9)
Supplement: Supplementary file 2 — Description of Additional Supplementary Files [file 41467_2025_64575_MOESM2_ESM.pdf]

Description of Additional Supplementary Files

Supplementary Movie 1: Assembly of the RD-TENG device array connection.

Supplementary Movie 2: The RD-TENG powers two parallel calculators.

Supplementary Movie 3: An array of four-RD-TENG powers two multimeters, one of which measures the voltage of a 1.5 V battery.

Supplementary Movie 4: Comparison of rectified electrode pairs lighting 32 × 2 W LED bulbs after sequentially adding 1 to 4 RD-TENGs in the array.

Supplementary Movie 5: The RD-TENG powers 5 × 2 W LED bulbs as a bicycle night-riding warning light.

Supplementary Movie 6: The RD-TENG system lights up a "GXU" sign composed of 284 LED lights connected in series in a wave tank.

Supplementary Movie 7: The RD-TENG system powers a temperature and humidity sensor in a wave tank.

Supplementary Movie 8: The RD-TENG system operates under real ocean conditions.

Supplementary Movie 9: The RD-TENG system powers two calculators on the sea surface after the ebb tide.
